# Ozone-enabled fatty acid discovery reveals unexpected diversity in the human lipidome

**DOI:** 10.1038/s41467-023-39617-9

**Published:** 2023-07-04

**Authors:** Jan Philipp Menzel, Reuben S. E. Young, Aurélie H. Benfield, Julia S. Scott, Puttandon Wongsomboon, Lukáš Cudlman, Josef Cvačka, Lisa M. Butler, Sónia T. Henriques, Berwyck L. J. Poad, Stephen J. Blanksby

**Affiliations:** 1 School of Chemistry and Physics, Queensland University of Technology, Brisbane, QLD 4000 Australia; 2 Centre for Materials Science, Queensland University of Technology, Brisbane, QLD 4000 Australia; 3 Centre for Data Science, Queensland University of Technology, Brisbane, QLD 4000 Australia; 4 School of Biomedical Sciences, Faculty of Health, Queensland University of Technology, Translational Research Institute, Brisbane, QLD 4102 Australia; 5 South Australian Immunogenomics Cancer Institute and Freemasons Centre for Male Health and Wellbeing, University of Adelaide, Adelaide, SA Australia; 6 South Australian Health and Medical Research Institute, Adelaide, SA Australia; 7 Institute of Organic Chemistry and Biochemistry of the Czech Academy of Sciences, Flemingovo náměstí 542/2, 16600 Prague, Czech Republic; 8 Department of Analytical Chemistry, Faculty of Science, Charles University, Prague 2, Czech Republic; 9 Present Address: Institute of Clinical Chemistry, Inselspital, Bern University Hospital, 3010 Bern, Switzerland; 10 Present Address: Faculty of Science, Medicine and Health, School of Chemistry and Molecular Bioscience, Wollongong, NSW Australia

**Keywords:** Lipidomics, Mass spectrometry, Cheminformatics, Liquid chromatography, Fatty acids

## Abstract

Fatty acid isomers are responsible for an under-reported lipidome diversity across all kingdoms of life. Isomers of unsaturated fatty acids are often masked in contemporary analysis by incomplete separation and the absence of sufficiently diagnostic methods for structure elucidation. Here, we introduce a comprehensive workflow, to discover unsaturated fatty acids through coupling liquid chromatography and mass spectrometry with gas-phase ozonolysis of double bonds. The workflow encompasses semi-automated data analysis and enables de novo identification in complex media including human plasma, cancer cell lines and vernix caseosa. The targeted analysis including ozonolysis enables structural assignment over a dynamic range of five orders of magnitude, even in instances of incomplete chromatographic separation. Thereby we expand the number of identified plasma fatty acids two-fold, including non-methylene-interrupted fatty acids. Detection, without prior knowledge, allows discovery of non-canonical double bond positions. Changes in relative isomer abundances reflect underlying perturbations in lipid metabolism.

## Abstract (web summary)

Fatty acids are fundamental biomolecular building blocks that are characterized by extraordinary structural diversity and present a formidable analytical challenge. Here the authors introduce a discovery workflow for de novo identification that adds more than 100 fatty acids to the human lipidome.

## Introduction

Advances in the molecular-level description of the genome, transcriptome, proteome, lipidome or metabolome over recent decades^1^, have opened new frontiers in medicine, biology, and nutrition^2^. The contemporary challenge of uncovering biomolecular diversity increases in complexity from genetics and proteomics to lipidomics due to the expanding array of molecular building blocks. Conversely, lipidomics thus presents a greater scope for biomolecular discovery due to the vast structural diversity of fatty acid building blocks^3^. Moreover, many fatty acids remain entirely undescribed or widely underreported, due to limitations in chromatographic and mass spectrometric sensitivity and selectivity. Notably, unsaturated fatty acid isomers with varied double bond position and configuration make up a major part of the diversity across the categories of lipids, where they are responsible for potentially thousands of structurally distinct molecular species; many of which are isomers of each other and have not been reported with full structural assignment^4^. Full lipidome coverage is a frontier challenge in lipidomics that can only be met by approaches that include a comprehensive survey of regio- and stereoisomeric fatty acid building blocks^5^.

Contemporary methods for fatty acid analysis are based on the hyphenation of chromatographic separation with mass spectrometric detection^6^. Despite the high peak capacity of modern gas chromatography columns and ultra-performance reversed-phase liquid chromatography, many fatty acids remain incompletely resolved^7^. In hyphenated chromatography-mass spectrometry methods, incomplete chromatographic resolution can be easily overcome when ionized lipids have different mass-to-charge ratios (*m/z*). For lipid isomers however, mass spectral discrimination relies on distinctive fragmentation patterns arising from ionization or ion activation. Unfortunately, the archetype ion activation approaches for fatty acids, namely electron ionization (for GC–MS) and collision-induced dissociation (CID, for LC–MS), often yield indistinguishable mass spectra for isomers of unsaturated fatty acids^8–10^. Methods for mass spectral discrimination of isomeric unsaturated fatty acids can broadly be grouped into two categories.

The first group of ion activation strategies have been optimized to promote wide-ranging fragmentation of carbon-carbon bonds resulting in a rich fragmentation pattern that is interrupted at, or adjacent to, the site(s) of unsaturation. Examples of these approaches include; (i) GC–MS strategies exploiting electron ionization of picolinyl esters (pyridyl carbinol derivatives) and dimethyloxazoline derivatives of fatty acids^11^, and (ii) LC–MS approaches such as electron impact excitation of ions from organics^12^, including methods based on electron activated dissociation with ZenoTOF mass spectrometers^13^, fixed-charge derivatives that promote charge-remote fragmentation^14^ and laser-based radical-directed dissociation methods^15^. These methods generate complex mass spectra and thus typically require full chromatographic separation of each isomer or are reliant on spectral libraries from analytical standards, because the overlay of complex spectra containing common product ions prevents unambiguous identification of individual isomers within such mixtures.

The second group of methods takes advantage of selective fragmentation of ionized fatty acids at, or adjacent to, carbon-carbon double bonds. Examples of this approach in the context of GC–MS include off-line dimethyl disulfide derivatization^16^, and online gas-phase derivatization at the double bond via covalent-adduct chemical ionization mass spectrometry^17,18^. Analogous LC–MS approaches include derivatization at the site(s) of unsaturation via Paternò-Büchi reactions that are often conducted between the chromatograph and the mass spectrometer^19,20^. Once chemically activated by derivatization, carbon–carbon double bonds are cleaved by CID leading to two characteristic product ions demarking each double bond position. Alternatively, atmospheric pressure chemical ionization after LC separation with subsequent CID yield spectra diagnostic for double bond positions^21,22^. Two further methods in this category are direct UV-photodissociation of ionized lipids^23^ and the gas-phase ion-molecule reaction with ozone; so-called ozone-induced dissociation (OzID)^24–30^. The latter is particularly suited for de novo identification of previously unknown regio- and stereoisomers at a high dynamic range, because mass selection of the fatty acid species can be performed prior to the ion activation and mass spectral analysis. In particular, the diagnostic OzID ions of any possible regioisomer are predictable and distinct, resulting in tandem mass spectra that allow de novo structural assignment across a wide dynamic range. Prior applications of OzID to a diverse range of lipids has led to numerous discoveries of lipids, confirming that the current understanding of the natural diversity in fatty acid building blocks is incomplete^31,32^. Several previous studies on OzID-MS are based on direct infusion without the capability to assign double bond stereochemistry, LC-OzID-MS has not been carried out for the purpose of comprehensive fatty acid profiling and no comprehensive automated data analysis pipeline was previously created for LC-OzID-MS.

Systematically surveying all fatty acids in complex lipidomes critically relies on robust data acquisition and automation in data analysis that does not introduce assumptions on possible structural diversity. Several software packages have been developed for lipidomics and metabolomics applications, such as the LC–MS based computational lipid library software LipidBlast^33^, the intact lipid analysis software MS-Dial 4^34^, the lipid class separation based quantification workflow LipidQuant^35^, the shotgun lipidomics based tools ALEX123^36,37^ and LipidXplorer^38,39^ among other tools^40,41^. These tools predominantly focus on the analysis of intact lipids, often only assigning fatty acyl chains at the level of numbers of carbons and the degree of unsaturation. Consequently, in addition to the analytical strategies above there is an urgent need for software tools that are specifically designed for the discovery of regio- and stereoisomers to fully map the lipidome at an increasingly complete structural level^42^.

Herein we introduce the combination of charge-tagged fatty acid analysis via LC-OzID-MS/MS including computationally built target lists for tandem OzID-MS and custom designed software for highly automated data analysis as an end-to-end workflow for the de novo, library independent, discovery of fatty acid isomers for complete fatty acid profiling. Termed ozone-enabled fatty acid discovery, or OzFAD, the analytical pipeline presented here is specifically created to interrogate the position and configuration of double bonds. The data analysis workflow, controlled through a graphic user interface (Supplementary Figs. 3 and S4), is engineered around the Skyline Mass Spectrometry Environment^43–46^ to allow intermittent user input and validation. The OzFAD methods are evaluated against selected aspects of the human lipidome, namely pooled human plasma, lipids from the skin of newborns (vernix caseosa) and human derived cancer cells (cell culture).

## Results

### Ozone-enabled fatty acid discovery (OzFAD) enables de novo identification of fatty acid double bond isomers

**Fig. 1:**
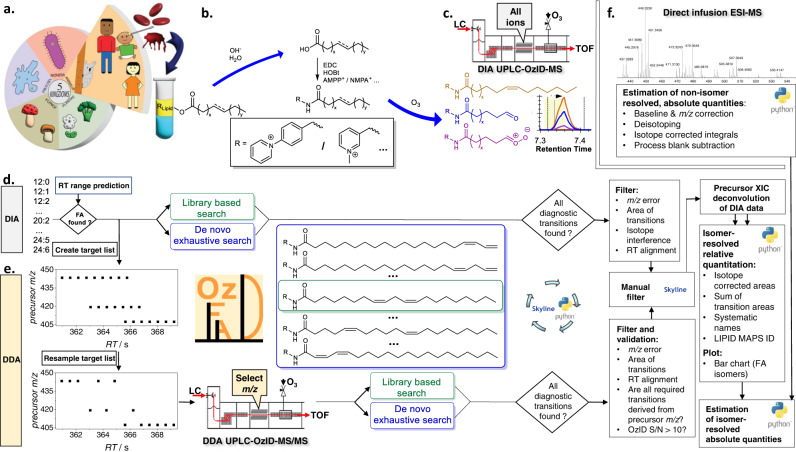
OzFAD: A de novo workflow for semi-automated fatty acid analysis with isomer resolution. **a** Lipids are extracted from human blood plasma, vernix caseosa or cell cultures. **b** After hydrolysis of lipids and addition of internal standards, fatty acids are derivatized with a fixed charge. **c** Liquid chromatography separates derivatized fatty acids, which undergo electrospray ionization (ESI) and are subjected to ozone-induced dissociation (OzID) with subsequent mass analysis (data-independent acquisition: DIA LC-OzID-MS). **d** Analysis of the DIA LC-OzID-MS dataset is initiated by a windows batch file that controls python scripts and instances of Skyline Runner. First, the retention times of precursors (unreacted, derivatized fatty acids) that can be identified in the dataset are saved. **e** Second, a target list (for a separate DDA LC-OzID-MS/MS run) is built based on precursor *m*/*z* values and retention times. After acquisition, an exhaustive search for all possible double bond positions (i.e., OzID product ions) and an automated filtering is carried out. Manual inspection in Skyline enables deletion of remaining false positives. Relative quantification is based on the DIA data including a manual correction of the deconvolution of extracted ion chromatograms. Finally, a python script formats the data, generates systematic names, retrieves LIPID MAPS IDs and common names from the LIPID MAPS database where available and generates a bar chart. The latter visualizes the relative abundance of fatty acid isomers and their double bond positions. **f** Direct infusion ESI-MS allows an estimation of abundance of fatty acid groups (no isomer resolution) and, combined with the relative abundance, an estimate of absolute quantities of each fatty acid.

Here we introduce a significant increase in the knowledge on molecular diversity of unsaturated fatty acids in the human lipidome. Critically, identification, including discoveries, are enabled by a comprehensive workflow (OzFAD) as a discovery platform for fatty acids in biological samples. OzFAD is a sophisticated approach to semi-automated data analysis including target list creation for data dependent acquisition of LC-OzID-MS/MS spectra that offers facile access to assigned spectra and relative quantitation without prior knowledge (Fig. 1).

**Fig. 2:**
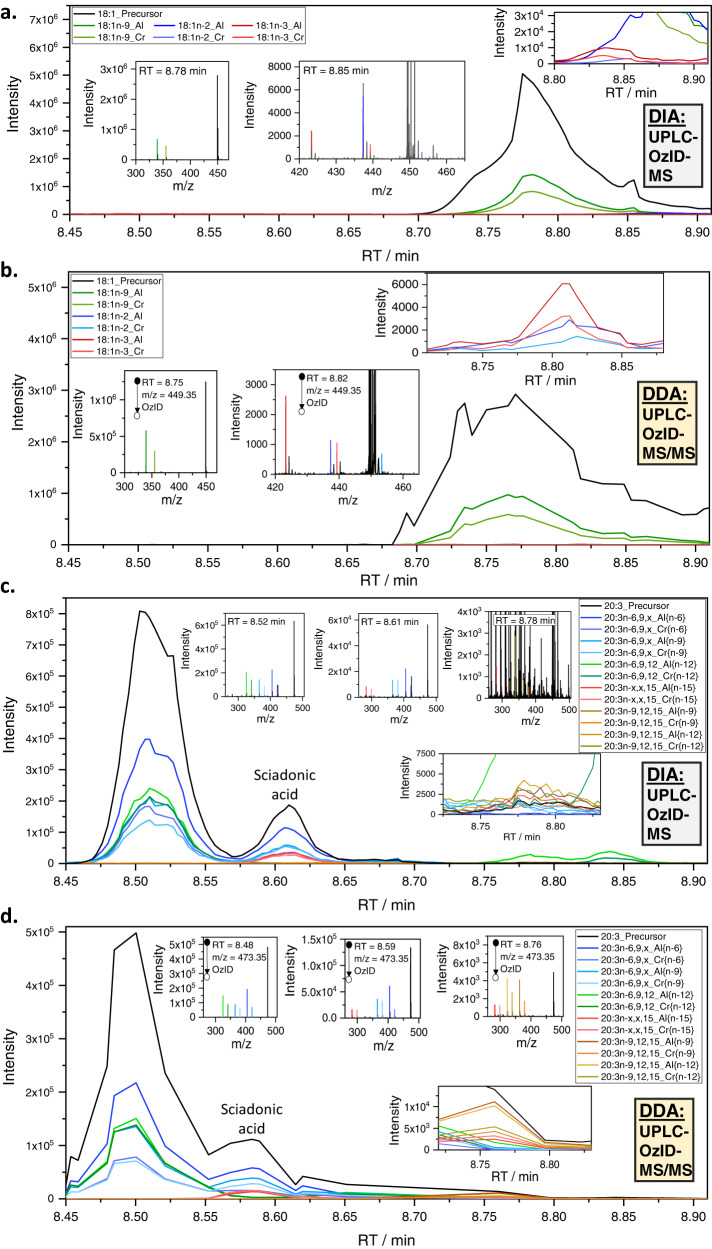
LC-OzID-MS(/MS) data for selected fatty acids in human plasma. Data obtained by DIA UPLC-OzID-MS (**a** and **c**) and the associated DDA UPLC-OzID-MS/MS acquisition (**b** and **d**) from AMPP derivatized fatty acids from hydrolyzed human plasma (NIST 1950 SRM). Shown are extracted ion chromatograms and (tandem) mass spectra consistent with identification of FA 18:1n-9 (oleic acid), FA 18:1*n*−2, FA 18:1*n*−3, FA 20:3*n*−6,9,12 (dihomo-γ-linolenic acid), FA 20:3*n*−6,9,15 (sciadonic acid), and FA 20:3*n*−9,12,15 (Mead acid). The extracted ion chromatograms of the data-dependent acquisition contain only a few points across each chromatographic peak, as multiple precursors are mass selected consecutively according to a highly segmented target list. Source data are provided as a Source Data file.

The workflow is based on the extraction of lipids from a biological matrix following the protocol of Matyash et al.^47^, subsequent hydrolysis and derivatization of fatty acids with a positive fixed charge as described by Bollinger et al.^14^. (Fig. 1a, b). Samples are loaded onto reversed-phase LC and analyzed by a travelling-wave ion-mobility mass spectrometer^27^ modified to deliver ozone to the ion-mobility region to promote efficient gas-phase ion-molecule reactions. No ion-mobility separation is performed herein. In this data-independent acquisition (DIA) there is no mass selection of ionized lipids, but all ions are subjected to ozonolysis and the resulting aldehyde and Criegee product ions arising from unsaturated fatty acids are assigned to precursor ions by retention time alignment (Fig. 1c, d). Conducting identification and relative quantification based only on the DIA approach (*vide infra*) is possible but challenging. In the absence of mass selection of the precursors, OzID product ions could be assigned to more than one coeluting fatty acid. Thus, to enhance confidence in structural assignments and to lower the limits of detection, a data-dependent acquisition (DDA), an LC-OzID-MS/MS experiment, is introduced. To accommodate this, the workflow branches into analysis of the data-independent acquisition (Fig. 1d), and generation of a target list for data-dependent acquisition (Fig. 1e). Applying an initial retention time range prediction for each precursor (the retention time of palmitic and stearic acid are used to predict retention time ranges of all other fatty acids at the sum composition level, Supplementary Note 1; Supplementary Fig. 12), those *m/z*-values with a precursor ion abundance above a user defined threshold and an associated *m/z*-error within another user-defined threshold (e.g., between 10 and 60 ppm, depending on the derivatization agent and instrument parameters), are selected within the respective retention time ranges (precursor analysis step). For each such precursor signal, targets are added to a raw target list (Fig. 1e). The raw target list is resampled, leading to a final target list, which contains one single precursor target at each defined retention time increment, with typically a total of 1000–3000 individual targets (*m/z* value for mass selection with associated retention time). The increments are defined in 0.3 s steps, enabling that for each increment at least one tandem MS measurement including mass selection, OzID of the mass selected precursor and analysis of the precursor and the respective OzID product ions in the time-of-flight analyzer is carried out. The resampling algorithm is designed to retain sufficient targets for each precursor that is detected, irrespective of abundance. This allows detection of low abundant fatty acids (e.g., mead acid, FA 20:3*n*−9,12,15) that are not isomers of co-eluting, highly abundant fatty acids (e.g., oleic acid, FA 18:1*n*−9) in human plasma (Fig. 2). Acquisition of tandem mass-spectra enables explicit assignment of OzID product ions to precursors, thus minimizing assignment errors while the enhanced signal-to-noise ratio (S/N) reduces the limits of detection. After acquisition, an exhaustive search for OzID product ions arising from all possible double bond positions can be conducted with subsequent automated filtering. Within this step, typically 100,000–800,000 instances of putative fatty acid assignments at associated varied retention times are created for processing with Skyline Runner, which are automatically filtered to a drastically reduced number, typically totaling between 150 and 500 instances, depending on the parameters of the analysis. Alternatively, the search can be limited to a subset of defined fatty acid species for cases where replicate or related samples have been processed already. Multiple analysis steps within Skyline runner and custom-written python code are automated and controlled by a windows batch file. The target list generated in the previous step is used to guide the search for matching precursor and product signals at the respective retention times. To prevent loss of correctly identified species within the workflow, the output Skyline file retains duplicate transitions and potentially false positive assignments. Curation of the list of putative assignments at this stage enables removal of duplicates and false-positives based on the following criteria: (i) visual inspection of extracted OzID product ion chromatograms allows deletion of duplicates (multiple transitions assigning the same chromatographic feature), (ii) the signal-to-noise ratio of the OzID product ions in tandem OzID mass spectra are to be determined with a separate python program, allowing all species for which S/N < 3 to be deleted from the Skyline file and those for which 3 < S/N < 10 to be tentatively identified (not subjected to quantification) and (iii) the MS/MS view in Skyline can also be used to visually validate that all OzID product ions are derived from the respective precursor (Supplementary Fig. 5).

An estimation of relative quantities of fatty acids is performed using the DIA dataset, while the DDA dataset aids in validating this process and providing a means to correct the outcome, if required. For example, the ratio of FA 20:3 isomers, particularly the relative quantity of Mead acid would be incorrect, if the DIA dataset was used without further consideration. Comparison with the DDA dataset shows, in which cases the extracted ion chromatograms of the OzID product ions reflect the isomer quantity and in which cases the extracted ion chromatogram in the DIA dataset may show falsely high intensity, that is not exclusively derived from the respective precursor. While extracted ion chromatograms of OzID product ions are used to establish peak positions, a deconvolution of the precursor extracted ion chromatogram is used for relative isomer quantification as this minimizes biases arising from differences in ozonolysis efficiency. The deconvolution is facilitated by the automatic generation of an Excel file with prefilled parameters and graphs for fitting of Gaussians representing each isomer centered on the OzID transitions. For samples originating from higher organisms, which may contain low-abundant coeluting isomers, several such species can only be quantified based on their OzID product ions. Thus, the relative quantity of these fatty acids represents a sophisticated best estimate (upper limit) with a mean accuracy of 4% of the reported value (based on the ratio of isomers present in a standard mixture of 37 fatty acids). Visualizations of relative abundances in bar charts and structural identifiers including systematic names are generated automatically by the python code. The LIPID MAPS® Lipidomics Gateway online database (https://www.lipidmaps.org/) is automatically searched for these systematic names. If an entry is found, the respective LipidMAPS ID and the listed common name are retrieved. This facilitates reporting of the findings and comparison with literature.

**Fig. 3:**
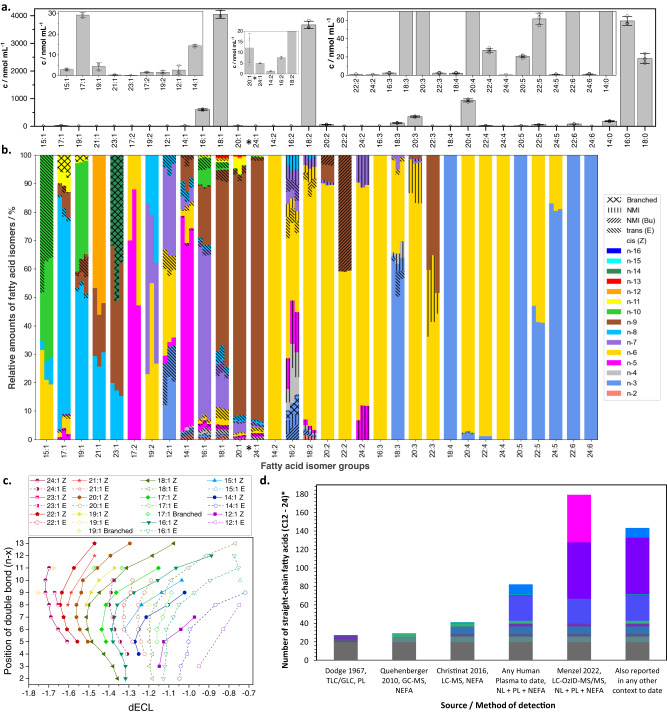
Analysis of fatty acids in human plasma (NIST 1950 standard reference material). **a** Quantification of plasma fatty acids at the sum composition level based on direct infusion ESI-MS. Each bar shows the mean of three technical replicates ± SD, *n* = 3. Selected saturated fatty acid quantities are shown, for the full profile see the Supplementary Note 3, Table S4. **b** Color-coded bar chart showing the different fatty acid isomers and their relative abundance as determined by LC-OzID-MS. Each technical replicate is represented by a vertical segment of each bar. Non-methylene-interrupted (NMI) fatty acids are highlighted by patterns distinguishing between butylene-interrupted (Bu) fatty acids and others. **c** Identified monounsaturated fatty acid species in the NIST 1950 human plasma and their relation between structure and retention time. Each data point represents a fatty acid isomer with double bond position as shown on the y-axis, plotted against dECL, the difference of the observed equivalent chain length (ECL) to the chain length of the associated saturated fatty acid. **d** Comparison of the numbers of straight chain fatty acids (C12–24) that are reported in human plasma, detected by different analytical methods and summarized literature surveys. Colors highlight fatty acid species that are found in multiple contexts or only reported in one of the displayed contexts. Source data are provided as a Source Data file. *Docosenoic acids (FA 22:1) are excluded from the analysis due to unavoidable contamination from plastics additive erucamide (FA 22:1*n*−9).

A best estimate of absolute quantities can finally be generated by Python scripts using direct infusion data (Fig. 1d). While absolute quantification is not a priority of this discovery workflow, it is advantageous to be able to access a measure of the contribution of each isomer to the total fatty acid pool. There are well recognized challenges in quantification in reversed-phase LC-MS of lipids due to changes in ionization efficiencies during gradient elution. To bypass this challenge, a parallel loop injection (direct infusion- or shotgun-ESI experiment without ozone) of 10 μL of the sample is undertaken. Isotope corrections are conducted on these data and the presence of the deuterated palmitic acid internal standard allows quantification of the fatty acid precursors at the sum composition level (i.e., the total number of carbons and degree of saturation, e.g., all FA 18:1 isomers) as illustrated in Fig. 3a. Parallel measurement of all fatty acid groups (without discrimination of isomers) by direct infusion ensures that ionization biases are reduced. These data enable relative quantitation of fatty acids at the sum composition level and reproduces the make-up of the reference mixture of fatty acids (based on reference FAs ranging from 12–24 carbons, Supplementary Fig. 11). For absolute quantitation, a final python script concludes the workflow by drawing on the sum composition quantitation of replicate data against the internal standards, producing the final best estimate including standard deviation and coefficients of variation for each fatty acid.

### Benchmarking OzFAD using a pooled human plasma standard reference material

Human blood plasma is one of the most studied biological samples in biomedical research^48^. To benchmark our discovery workflow, we used NIST 1950 human plasma as a well-studied, commercially available reference. This sample combines blood plasma of 100 American volunteers and is widely accepted as a reference material to standardize the field of lipidomics^49,50^. After processing of three replicates with the OzFAD workflow, comparison of quantities of unsaturated fatty acids observed with certified values reported for this reference material^51^ reveals an accuracy of 63% (mean average deviation) for the OzFAD workflow (Supplementary Fig. 25) versus accuracy (mean average deviation) of participating laboratories in the same interlaboratory study^52^ ranging from 11% to 62%. The range of reference values reflects the challenge of absolute quantification in lipidomics. Further enhancement of the quantification method described herein (e.g., through wider availability of fatty acid standards) is beyond the scope of this study.

Figure 2a, c shows example extracted ion chromatograms from DIA LC-OzID-MS of lipids extracted, hydrolyzed and derivatized from the reference human plasma. As expected, the data readily identify abundant oleic acid (FA 18:1*n*−9) by retention time alignment (8.78 min) of the precursor ion at *m/z* 449.35 and the OzID product ions at *m/z* 339.21 and 355.20. Importantly, the selectivity and dynamic range of the OzFAD analysis reveals many more features corresponding to double bond regioisomers, for example FA 18:1*n*−2 and FA 18:1*n*−3 (where the *n*−x nomenclature indicates the position of the double bond with respect to the methyl terminus), despite being incompletely resolved by chromatography (Fig. 2a inset). These putative identifications are confirmed by the targeted DDA LC-OzID-MS/MS steps that uniquely assign the OzID product ions to the selected precursor ion for each double bond positional isomer. In addition, the greatly improved signal-to-noise ratio in the tandem mass spectrum enables reconstruction of the discrete retention time of each isomer (Fig. 2b). This is further highlighted by the identification of a fatty acid with composition FA 20:3, which co-elutes with the significantly more abundant FA 18:1 isomer family.

While the data-independent acquisition does not produce unambiguous chromatographic or mass spectral evidence of the double bond positions of FA 20:3 at 8.78 min (Fig. 2c, insets), the data-dependent tandem mass spectra unambiguously identify this fatty acid as FA 20:3*n*−9,12,15 (mead acid) (Fig. 2d, insets). Each aldehyde and Criegee diagnostic product ion for each double bond is detected well above noise in the tandem mass spectrum and the diagnostic ions evidently arise from ozonolysis (OzID) of the mass-selected FA 20:3 precursor. In cases of fatty acids that are well-known, abundant and chromatographically resolved from other features, such as FA 20:3*n*−6,9,12 (dihomo-γ-linolenic acid, 8.50 min), the DIA LC-OzID-MS analysis (Fig. 2c, insets) may sufficiently support identification. In contrast, for unexpected features, such as FA 20:3*n*−6,9,15 (sciadonic acid, 8.60 min), confirmation by the characteristic OzID product ions in the DDA tandem MS acquisition is critical for confident identification. We further carried out GC-CACI-MS/MS experiments to demonstrate that sciadonic acid can be detected in the human plasma NIST 1950 SRM by orthogonal methods, see Supplementary Note 3 and Supplementary Fig. 26.

Harnessing the full analytical power of the workflow, we identified a total of 186 fatty acids in the NIST 1950 SRM that meet the stringent criteria for identification, including the retention time range prediction and observation of precursor and diagnostic OzID product ions in the tandem MS acquisition with a signal-to-noise ratio for the OzID product ions above 10 (Fig. 3). Combining the absolute quantification at the sum composition level (Fig. 3a) with the relative quantification of the isomers (Fig. 3b) it is possible to determine the concentration of all fatty acids identified by this workflow (Supplementary Data 1). This analysis establishes the dynamic range of confidently assigned fatty acid species to five orders of magnitude, with oleic acid (FA 18:1*n*−9 *cis*) being detected at 2.5 ± 0.1 μmol mL^−1^, whereas FA 24:1*n*−6 *cis* is observed at 0.028 ± 0.006 nmol mL^−1^. Three replicates of the fatty acid analysis of NIST 1950 SRM pooled human plasma are displayed (Fig. 3b). Each group of fatty acids, such as dodecenoic acids are represented as a segmented bar, showing the relative abundances of replicate 1 on the left and replicate 2 and 3 on the middle and right, respectively, of each bar. Both identification as well as relative quantification are reproducible, as any species found to be present above noise was detected in all three replicates. The visual representation highlights that variations between technical replicates are smaller for fatty acids with a high overall abundance, for example in the case of octadecenoic acids (FA 18:1) a mean coefficient of variation (COV) of the relative isomer abundance of 0.2 is determined, whereas variations are larger for fatty acids with low overall abundance, such as nonadecadienoic acids (FA 19:2) with a mean COV of 0.5.

Configuration of double bonds of monounsaturated fatty acids is assigned here by comparison of retention times with other fatty acids. The retention times of fixed-charge derivatized fatty acid isomers in reversed-phase chromatography are systematically affected by double bond position and configuration (Supplementary Fig. 22). The concept of equivalent chain length (ECL) or equivalent carbon number was introduced to rationalize trends in fatty acid methyl ester retention times in gas chromatography and to compare values between different chromatographic runs that may be affected by retention time shifts. We found that the difference between observed equivalent chain length and chain length of the respective saturated fatty acid (differential equivalent chain length, dECL) is a measure that allows comparison of fatty acid isomer retention behavior across varied chain lengths and double bond positions (Fig. 3c; Supplementary Fig. 23). The trends in dECL values of all monounsaturated fatty acids that we detected in human plasma enable assignment of fatty acid structures through extrapolation and interpolation of dECL values across fatty acids with varied chain lengths and double bond positions. This strategy has limitations when considering the stereochemistry of polyunsaturated fatty acids, although the respective analyses reveal clear trends for several methylene-interrupted fatty acids (Supplementary Figs. 24, 30, 37 and 41). In general, branched chain fatty acids elute earlier than straight chain fatty acids (comparing fatty acids with the same number of carbons overall), while fatty acids with *cis* double bonds (connected by solid lines in Fig. 3c) elute earlier than their *trans* counterparts (connected by dashed lines in Fig. 3c). For example, the configuration of FA 17:1*n*−11 *cis* (6Z-heptadecenoic acid) is confirmed by comparing its dECL value of −1.37 to the dECL values of other detected heptadecenoic acids as well as to hexadecenoic and octadecenoic acids considering their double bond position. Thereby, the other two instances of FA 17:1*n*−11 are identified as branched unsaturated fatty acids, although no assignment of the number and position of the branch points can be made by LC-OzID-MS. The equivalent carbon number concept was recently also employed to annotate lipids in reversed-phase liquid chromatography across multiple lipid classes^53^. We found that *trans* fatty acids make up 6.5% of the total fatty acid content of the reference plasma.

We surveyed the literature to assess whether each of the fatty acids we identified in the NIST 1950 SRM had been identified previously in human plasma, was reported in any other context, or represents a newly discovered fatty acid (Supplementary Table 4 and 5). The outcomes of this survey are shown in detail in Table S4 (Supplementary Note 3) and numbers of detected fatty acids are summarised in Fig. 3d. The bar chart in Fig. 3d displays the numbers of straight-chain fatty acids (C12–24, excluding FA 22:1) that were identified in three selected publications^48,54,55^ compared to the number identified in all publications that were part of our literature survey as well as the ones that were also identified in any biological context to the best of our knowledge. The color-coded areas represent fatty acids that were commonly found in the varied contexts. In addition, we identified five branched chain fatty acids with the limitation of not being able to determine the position of the branch point(s). Further, thirteen docosenoic acids were observed, which are excluded from the comparison due to the presence of erucamide and isomers of this erucic acid derivative in the associated process blank. Finally, an additional 33 di-unsaturated fatty acids are only tentatively identified due to incomplete chromatographic separation. Future work on longer chromatographic gradient methods for LC-OzID-MS is expected to increase the number of confidently assigned fatty acids. Given that some prior investigations of human plasma had focused on non-esterified fatty acids (NEFA), we carried out a derivatization using a non-hydrolyzed lipid extract from the same pooled human plasma reference material (Supplementary Fig. 26 and Supplementary Data 2). This analysis led to a similar profile of fatty acids, with only one additional isomer (FA 23:1*n*−6, very low abundance) being detected in NEFA, that was not found in the total hydrolyzed lipid extract. This is likely the result of a “missing target” in the target list for the respective retention time in the analysis of hydrolyzed fatty acids. However, several low-abundant species that were identified in the hydrolyzed lipid extract were not detected above the signal-to-noise threshold in the NEFA analysis. Thus, analysis of hydrolyzed lipid extracts with OzFAD provides an excellent foundation to help establish overall lipid diversity (Supplementary Note 3 and 4). Considering the analysis of total fatty acids, our workflow led to the discovery of 51 fatty acid isomers (including FA 18:1*n*−2 *cis* and 29 *trans* fatty acids) that, to the best of our knowledge, have previously not been reported, while an additional 59 fatty acids have not been reported in human plasma, but have instead been identified in other biological sources (including FA 18:1*n*−3 *cis*). Among the identified fatty acids, twelve are non-methylene-interrupted fatty acids including sciadonic acid (FA 20:3*n*−6,9,15 at 47 ± 8 nmol mL^−1^) and keteleeronic acid (FA 20:2*n*−9,15 at 2.4 ± 0.4 nmol mL^−1^). Polyunsaturated fatty acids that exhibit a pattern of unsaturation other than the canonical methylene-interrupted or conjugated sequences of double bonds have not previously been reported in human plasma but have been observed in other biological systems including pine nuts^56^, sea urchins^18^, mangos^57^, marine food webs^58^, dairy^59^, seed oils^22^ as well as human breast milk^60^. Prior identification of non-methylene-interrupted fatty acids in foodstuffs may point to these being present in reference plasma as a result of dietary intake. However, the co-detection of unsaturated fatty acids that would serve as potential intermediates in the biosynthesis of these species suggests the intriguing possibility that human lipid metabolism may also be capable of generating them. Therefore, to investigate the diversity of human lipid metabolism, we demonstrate the capability of the OzFAD workflow in lipids derived from vernix caseosa and human-derived cell lines.

### OzFAD uncovers non-canonical fatty acids in vernix caseosa

Vernix caseosa, the white waxy layer that newborns are covered in at birth, is known for complexity and perversity of fatty acid structures with up to 167 chromatographic features previously attributed to distinct fatty acids but without complete structural assignment^61,62^. The vernix caseosa fatty acid profile is characterized by very long chain fatty acids, extensive chain branching and unusual positions of unsaturation including high abundance of sapienic acid (FA 16:1*n*−10 *cis*)^63,64^. Analysis of vernix caseosa was here conducted using a total lipid extract from a pooled sample from 10 female and 10 male newborns, delivered at full term, according to the workflow described herein with the 4-iodo-AMPP derivatization agent utilized in place of AMPP (Supplementary Fig. 31 and Table S6)^63^. De novo assignment of sites of unsaturation was conducted as previously described, with fatty acids assigned as branched or straight chains based on an analysis of retention time indices (Supplementary Figs. 28–30). While assignment of the explicit site of methyl chain branching (e.g., *iso* or *anteiso*) is beyond the scope of this method, unsaturated fatty acids with straight chain and branched chain variants were well-resolved by chromatography allowing ready classification. For example, a branched fatty acid with 17 carbon atoms in total, a methyl branch in the antepenultimate position (*anteiso*) and a double bond in the Δ7 position (FA 14Me-16:1*n*−9 *cis*; (7Z)−14Me-hexadecenoic acid) would be classified by OzFAD as FA 17:1*n*−10 and due to the retention time alignment and differential equivalent chain length (dECL) analysis it would be assigned as branched. As both chain branching and double bond positions influence retention times, the unequivocal assignment of double bond positions will aid in the full structural identification of all fatty acids in samples that contain more than 150 individual fatty acids, such as vernix caseosa by future extensions of this workflow incorporating photodissociation of the photolabile 4-iodo-AMPP derivatization agent^63^.

**Fig. 4:**
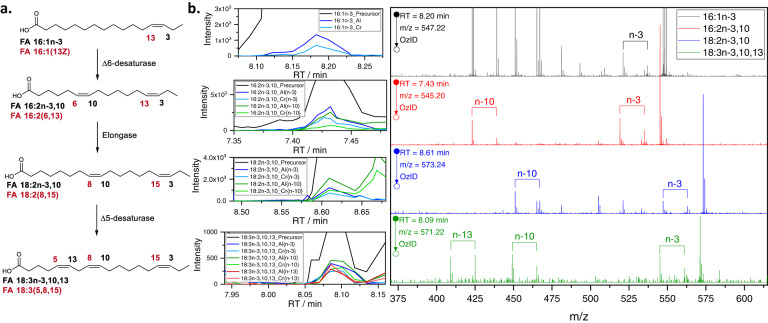
Discovery of non-methylene-interrupted ω-3 fatty acids in vernix caseosa. **a** A proposed biosynthetic pathway rationalizing the discovery of the never previously reported fatty acids FA 16:2*n*−3,10 (6,13-hexadecadienoic acid); FA 18:2*n*−3,10 (8,15-octadecadienoic acid) and FA 18:3*n*−3,10,13 (5,8,15-octadecatrienoic acid). The double bond configuration is tentatively assigned as *cis* in each case. **b** Associated extracted ion chromatograms and OzID MS/MS spectra of the 4-I-AMPP derivatized fatty acids. For the complete fatty acid profile, refer to the Supplementary Note 5, Supplementary Fig. 31 and Supplementary Data 3. Source data are provided as a Source Data file.

Similar to the scale of discoveries in human plasma, our workflow revealed an unexpected diversity of double bond positions and patterns, including 18 straight chain saturated, 116 straight chain monounsaturated, 32 straight chain polyunsaturated, 30 branched saturated and 42 branched monounsaturated fatty acid species out of a total number of 238 fatty acids (Fig. 4 and Supplementary Note 5) of which 40 have not been reported previously in any context including the analysis of human plasma within this work (Supplementary Table 6). Further, trends in relative abundances of monounsaturated fatty acids of increasing chain lengths (16–30 or 17-29 carbons, respectively) were observed, with *n*−5 to *n*−7 monounsaturates making up an increasing percentage of the respective isomer groups, whereas the proportion of *n*−9 to *n*−12 monounsaturates decreases accordingly. Such observations may be indicative of differential elongation rates depending on double bond positions (Supplementary Fig. 31). In addition to monounsaturates, the sensitivity of the OzFAD approach enabled identification of several never-previously- reported non-methylene-interrupted polyunsaturated fatty acids, which could represent intermediates in a yet-to-be-described metabolic pathway (Fig. 4 and Supplementary Fig. 31). For example, Fig. 4 presents mass spectral evidence for the presence of FA 16:1*n*−3*cis* that could plausibly undergo Δ6 desaturation (e.g., via FADS2) to yield FA 16:2*n*−3,10. Subsequent elongation could yield FA 18:2*n*−3,10 followed by Δ5 desaturation (e.g., via FADS1) to give FA 18:3*n*−3,10,13. We additionally performed LC-UVPD-MS/MS to demonstrate that these three newly discovered ω−3 polyunsaturated fatty acids can also be detected by orthogonal methods, see Supplementary Note 5 and Supplementary Figs. 32 and 33. While mechanistic studies are required to demonstrate the proposed biosynthetic pathway unequivocally, the unambiguous detection of all intermediates in this pathway provides a strong foundation for the hypothesis that human (or, alternatively, human microbiome^65^) lipid metabolism is capable of the biosynthesis of these ω−3 non-methylene-interrupted fatty acids. Overall, the OzFAD workflow critically aids the discovery and investigation of unexpected double bond patterns that occur in low abundant fatty acids and co-elute with known isomeric structures.

### OzFAD for quantifying changes in lipid metabolism of cancer cell lines

The role of lipids and fatty acids in cancer metabolism is gaining increased interest^66–71^. Here, we applied the OzFAD pipeline to characterize the total fatty acid profiles of three cancer cell lines (i.e., MCF7, LNCaP and LNCaP_SCD1*i*; SCD-1 inhibition: A939572) and investigate the effect of enzyme expression and inhibition of stearyl-CoA desaturase-1 (SCD-1/Δ9-desaturase) (Supplementary Note 7).

**Fig. 5:**
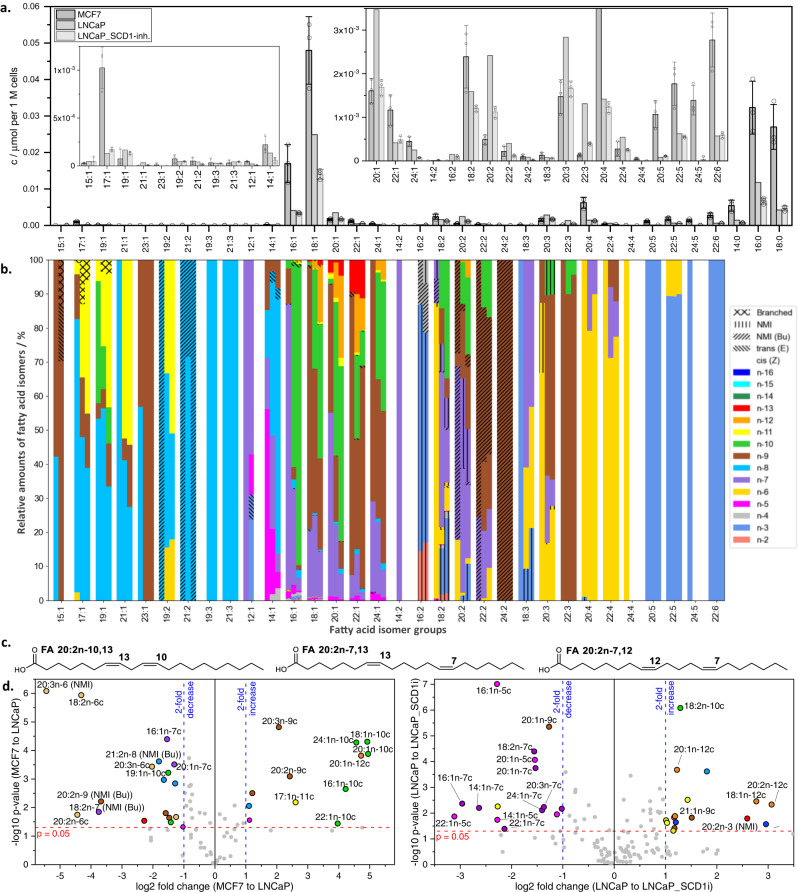
Fatty acid profiles of breast (MCF7) and prostate (LNCaP) cancer cell lines, also featuring inhibition of SCD1. **a** Estimated fatty acid quantities by direct infusion ESI-MS. Shown are mean values ± SD of three biological replicates (*n* = 3). Due to interference of ion suppressing contaminants in two replicates of LNCaP, only one is shown here, instead of the mean of three replicates. **b** Relative quantification of fatty acid isomers in the three cell lines by LC-OzID-MS and LC-OzID-MS/MS. For each isomer group, a segmented bar is shown, where the segments on the left represent fatty acids in MCF7 cancer cell line extracts, the segments in the middle of each bar represent fatty acids in LNCaP cancer cell line extracts and segments on the right, respectively, represent LNCaP_SCD−1*i* cancer cell line extracts. Shown are mean values of relative abundances of three biological replicates of each cell line. For individual values and standard deviations, refer to the Supplementary Information. **c** Example eicosadienoic acids in either MCF7 or the LNCaP cell lines. **d** Volcano plots visualizing the fold changes (and associated *p*-values based on two-sided Welsh *t*-tests) in relative fatty acid isomer abundances as compared between MCF7 and LNCaP (left volcano plot) and between LNCaP and LNCaP_SCD1*i* (volcano plot on the right), see also Supplementary Figs. 43 and 44 and statistical details in Supplementary Data 8 and 9. Selected isomers are labelled to show examples of large changes (exceeding twofold changes, blue dashed lines) in relative isomer abundances within the respective fatty acid isomer groups with an associated *p*-value above 0.05 (red dashed line). Source data are provided as a Source Data file.

The MCF7 cell line originates from human breast cancer and has high expression of SCD-1, whereas LNCaP originates from a metastatic human prostate cancer that has previously been shown to exhibit non-canonical pathways of desaturation and elongation. Findings from the application of the OzFAD protocol to these cell lines are summarized in Fig. 5.

While the direct infusion data, shown in Fig. 5a, indicates differences in the abundance of fatty acid isomer groups, only the isomer-resolved LC-OzID-MS analysis (Fig. 5b and Supplementary Data 5, 6 and 7) reveals significant underlying changes in the presence and abundance of distinct molecular species (Supplementary Figs. 38, 39 and 42). Further, a comparison of the number of octadecenoic acids in LNCaP cancer cell lines identified in our previous work based on high-resolution direct-infusion OzID-MS to the LC-based OzFAD workflow indicates an increase from 7 species^31^ to 17 fatty acids (Supplementary Data 6 and Supplementary Fig. 39). The effect of lowered and inhibited Δ9-desaturation corresponds to the relative amounts of several species, as demonstrated by the volcano plots (Fig. 5c). Inhibition of SCD-1 activity shows the metabolic shift from *n*−5, *n*−7 and *n*−9 monounsaturates (e.g., FA 14:1*n*−5, FA 16:1*n*−7) to the *n*−10 and *n*−12 counterparts (FADS2 Δ6-desaturation products) and subsequent desaturation and elongation products (from sapienic acid FA 16:1*n*−10 to FA 22:3*n*−10,13,16), in line with previous findings (Supplementary Data 7)^31,72^. Interestingly, the pathway to FA 22:4*n*−7,10,13,16 appears significant in LNCaP cells, but not in MCF7 cells.

Some metabolic shifts may be masked by conventional analysis of fatty acids, whereas the wide dynamic range of the OzFAD workflow reveals even trace fatty acids in the media for cell culture. Here we analyzed fetal bovine serum (Supplementary Fig. 34 and Supplementary Data 4), to establish the fatty acid profile of the culture media. While bovine and human fatty acid profiles are known to be different^73^, OzFAD analysis again uncovers a diverse set of isomers (Supplementary Table 7). Comparison of fatty acid profiles of the cells and the culture media (fetal bovine serum) indicates that, for example, the *n*−7,13 butylene-interrupted pattern observed in FA 18:2, 20:2 and 22:2 is present in MCF7 cell extracts, but not in the media or in the LNCaP cells, revealing a distinct metabolic pathway (Supplementary Fig. 42). We submit the hypothesis that a biosynthetic route via Δ5-desaturation (FADS1) of FA 18:1*n*−7 yields FA 18:2*n*−7,13 and likewise FA 20:1*n*−9 leads to FA 20:2*n*−9,15 (Supplementary Note 7, Supplementary Fig. 35). Elongation of these leads to chain lengths up to 22 and 24 carbons, respectively. The same mechanism for the formation of butylene-interrupted fatty acids could apply to odd chain fatty acids FA 19:1*n*−8,14 and FA 21:1*n*−8,14. All non-methylene-interrupted fatty acids that are derived from Δ5-desaturation, including sciadonic acid (FA 20:3*n*−6,9,15), are present in significantly higher ratios in MCF7 cells compared to LNCaP cells. On the other hand, the non-methylene-interrupted fatty acid FA 20:2*n*−7,12 is only found in LNCaP cells, but not in the culture media or in MCF7 cells, indicating the possibility of a Δ6 desaturation pathway from FA 20:1*n*−7. Discovery of fatty acids is critical to reveal substrate specificity of desaturases and elongases and to complete human lipid profiles including their biological drivers^74–77^.

## Discussion

Human-derived lipids from blood plasma, vernix caseosa and cancer cells contain a significantly larger number of unsaturated fatty acids than previously reported. The discoveries presented here are enabled by the herein introduced end-to-end workflow OzFAD for the de novo identification of fatty acids from complex biological samples. Compared to other strategies to elucidate double bonds, the OzFAD workflow features two distinct advantages. Firstly, the de novo data analysis relies on no assumptions of which fatty acids may be present in the sample, allowing the detection of unexpected sites of unsaturation and making the workflow a true discovery tool. The analysis proceeds *via* a stepwise process involving an automated, exhaustive isomer search and filtering, augmented by intermittent user input for quality control. Thereby, limitations of the workflow, such as a small degree of over-oxidation during OzID can be accounted for. Secondly, computationally-generated targets for targeted acquisition allows mass-selection of all precursors that are present without direct user intervention. Carrying out OzID on the mass-selected precursor ions yields confident assignments of all unsaturated fatty acids across five orders of magnitude with signal-to-noise values for the ozonolysis products above ten. This analysis, based upon the specific and predictable OzID product ion generation, enables tracking a multitude of co-eluting fatty acid isomers even across vastly different concentrations, alleviating the need for complete chromatographic separation or for reference mass spectra. We aimed to combine the best of automation and human intelligence to streamline the discovery process, avoiding false positive or negative identification.

The application of retention time analysis on large pools of fatty acid isomers has enabled augmentation of the fatty acid structural assignment beyond double bond location to include double bond configuration and the identification of methyl branching. Currently, the method cannot explicitly assign sites of chain branching but this can be overcome in the future by the introduction of parallel analysis of samples using radical-directed dissociation^15^ or similar methods. Importantly, the effective application of the I-AMPP derivatization as well as the archetype AMPP derivative indicates that these methods can be run in parallel on the same derivatized extracts. While the workflow unmasks structural fatty acid diversity to a level not seen before, future work will focus on improving quantitation accuracy across the vast number of fatty acids that can now be identified as well as improving chromatographic separation. Central to these advances will be improved availability of reference standards, both standards for many of the fatty acids that have been discovered herein, as well as isotopically labelled standards for each isomer group. Further, the workflow can be applied to the analysis of lysophophatidylcholine (LPC) and lysophosphatidylethanolamine (LPE) and cholesteryl esters (CE). Further extension of the logic of this workflow to complex lipids with two (or more) acyl chains will require a means to selectively remove and modify each acyl chain prior to introduction to this pipeline^78^. Conceivably, this could be achieved off-line by incorporating selective enzyme hydrolysis with multi-stage fractionation or in advanced multistage mass spectrometry modalities incorporating multiple selective ion-chemistries (e.g., emerging multistage ion-ion workflows)^79^.

Application of the OzFAD tool has already underpinned the discovery of 107 fatty acids, not previously reported in human plasma. Of these, 51 fatty acids are, to the best of our knowledge, discovered here, as no previous reports could be found. All but one species that were identified in non-esterified fatty acids in human plasma were also found in the total fatty acid fraction (hydrolyzed lipids), indicating that the analysis of hydrolyzed lipids by this method can inform future in-depth studies of intact lipids by LC-OzID-MS/MS. Thereby, we anticipate an expansion of lipidome coverage on a similar scale compared to the discoveries shown herein. Many of the discoveries revealed here correspond to *trans* monounsaturates, whereas some non-methylene-interrupted species were detected. Interestingly, sciadonic acid is found at significant concentrations in the human plasma standard reference material NIST 1950. The discovery of such non-canonical patterns of unsaturation in vernix caseosa and human cell lines indicates that hitherto undescribed lipid metabolism may be responsible for the synthesis or modification of lipids leading to production of such species. The depth of the analysis shown here sets standards for the identification of fatty acid double bond isomers. Our findings and future work are expected to have far-reaching consequences for lipidomics and metabolomics studies involving cell culture, healthy and diseased human tissues, cosmetics, supplements, the human diet as well as plant, microorganism, and animal metabolism. The OzFAD workflow is a tool to tackle the challenge of lipidome coverage at a deeper level than previously achieved, adding to the toolkit of methods to study highly complex interactions, such as those between the human microbiome(s) and the human metabolism.

## Methods

### Ethics declarations

Vernix caseosa was collected by the group of Josef Cvačka, Institute of Organic Chemistry and Biochemistry, Academy of Sciences of the Czech Republic, Prague, Czech Republic, with written informed parental consent and the work was approved by the Ethics Committee of the General University Hospital, Prague (910/09 S-IV) and was conducted in accordance with the principles of the Declaration of Helsinki. Material collected from 10 female and 10 male newborns (delivered at full term) was pooled to create a reference sample for vernix caseosa.

### Nomenclature

Fatty acids are described within this work using either systematic names (sciadonic acid is 5Z,11Z,14Z-eicosatrienoic acid; indicating the double bond positions from the carboxyl end of the fatty acid) or accepted shorthand notations using the *n*-nomenclature (sciadonic acid is either FA 20:3*n*−6,9,15 or briefly 20:3*n*−6,9,15; indicating double bond positions from the methyl terminus). Configuration of carbon-carbon double bonds is denoted using either E/Z, *trans/cis* or briefly t/c, such that oleic acid is either systematically 9Z-octadecenoic acid, or FA 18:1*n*−9 *cis* (or briefly 18:1n-9c).

### Cell culture and lipid extraction

Human prostate cancer cell line LNCaP (clone FGC) was obtained from the American Type Culture Collection (ATCC CRL-1740; RRID:CVCL_1379) and authenticated by short-tandem repeat profiling at Cell Bank Australia (NSW, Australia) in July 2020. Human breast cancer cell line MCF7 was obtained from the American Type Culture Collection (ATCC HTB-22; RRID:CVCL_0031) and authenticated by short-tandem repeat profiling at QUT Genomics Research Center as a 100% match in July 2019. Cells were cultured in RPMI-1640 medium (Life Technologies) containing 10% (v/v) fetal bovine serum (FBS) and 2 mmol L^−1^ L-glutamine (Life Technologies) in 5% CO_2_ in a humidified atmosphere at 37 °C and subjected to regular mycoplasma testing. For SCD-1*i* treatment, cells were seeded in triplicate at 4.5 × 10^5^ cells/well in 6-well plates overnight, before being treated with 1 μM A939572 (Tocris) using dimethylsulfoxide as a vehicle and cultured for 72 h, then collected for lipid extraction. Human breast cancer MCF7 cells, obtained from the American Type Culture Collection, were grown in Dulbecco’s modified eagle medium (DMEM) medium complemented with 10% (v/v) fetal bovine serum (FBS; heat inactivated for 30 min at 56 °C) and 1% (v/v) penicillin/streptomycin in an incubator set to 37 °C with 5% CO_2_. The cell culture was profiled via short tandem repeat profiling and regularly tested for mycoplasma.

Lipids were extracted following a protocol by Matyash et al.^47^ and modified by Young et al.^31^ Cells were trypsinized and counted with an automated TC-20 cell counter (BioRad) and 1 × 10^6^ (in the case of LNCaP and LNCaP_SCD1i: 5 × 10^6^ and subsequently adjusted solvent volumes) cells were pelleted in microfuge tubes (in triplicate). The cell pellets were washed twice with Dulbecco’s phosphate-buffered saline (DPBS) and stored at −80 °C. Lipids were extracted in 2 mL glass vials and a stock solvent mixture containing 370 μL methyl tert-butyl ether (MTBE) with 0.01% butylated hydroxytoluene (BHT). Cell pellets were dispersed in 110 μL methanol and 390 μL solvent mixture and vortex mixed for 20 s with subsequent continuous agitation for 1 h. After addition of 100 μL ammonium acetate (150 mM) and vortex mixing for 20 s, the phases were separated by centrifugation for 5 min at 2000 × *g*. The organic phase was collected and stored at −80 °C.

### Lipid extraction from NIST1950 SRM (pooled human plasma)

After thawing and sonication for 5 min, the NIST Human Plasma was found to be a mixture of solids dispersed in the liquid phase. To reliably assess the total fatty acid content of the human plasma sample, 800 μL of the total mixture were collected and divided equally into four vials. As internal standard, FA 16:0d31 (perdeuterated palmitic acid) was added to a final concentration of 1.348 μmol L^−1^. Lipids were extracted as per the procedure based on Matyash et al.^47^. The lipid extract was then pooled and equally divided over four individual vials for further treatment, hydrolysis and subsequent derivatization. Three aliquots were taken from the vials for further analysis. Thus, the standard deviation of each of the three technical replicates of the NIST Human Plasma incorporates the statistical experimental error of hydrolysis, derivatization, instrumental analysis, and data analysis.

### Hydrolysis, fatty acid extraction and fixed-charge derivatization

The sensitivity of fatty acid detection by mass spectrometric methods is reported to increase 10 – 20 fold by derivatization with a fixed positive charge, such as 1-(4-(aminomethyl)phenyl)pyridinium (AMPP) compared to analysis in negative mode^80,81^. Thus, either AMPP or the iodo-substituted derivatization agent 4-I-AMPP^63^ were used in this study to investigate the total fatty acid content of the samples.

To the vial containing the residue of the lipid extraction 0.2 mL methanol and 0.2 mL aqueous tetrabutylammonium hydroxide solution (40% w/w) were added, and the mixture was heated at 75 °C for two hours. Subsequently, 1.5 mL pentane, 1.5 mL water and 0.3 mL concentrated hydrochloric acid were added. The mixture was vortex stirred for 1 min. After allowing the phases to separate, the organic layer (top layer) was collected. 1.5 mL pentane were added to the residual aqueous phase and extraction was repeated. The organic phases were combined and 3 μL *N,N*-diisopropylethylamine, and 0.15 mL acetonitrile/dimethylformamide 4:1 (v/v) was added. The biphasic mixture was vortex stirred for 1 min, before the top layer was evaporated by a stream of nitrogen. To the vial containing the residual liquid from the hydrolysis procedure 10 μL of a freshly prepared 1 M aqueous solution of *N-*ethyl*-N*′-(dimethylaminopropyl)carbodiimide hydrochloride was added, before 40 μL of a 30 mM solution of 1-hydroxybenzotriazole nonahydrate in acetonitrile was added. The solution was vortex stirred for one min. 0.1 mL of a 20 mM solution of AMP^+^ (Cayman Chemical) in acetonitrile/dimethylformamide 4:1 (v/v) was added, and the mixture was vortex stirred for 1 min and heated to 65 °C for 30 min. After allowing the solution to cool to ambient temperature, 1 mL methanol was added. The solution was diluted six-fold and all volatiles were removed by evaporation under a stream of nitrogen. The residue was dissolved in 1 mL methanol and the sample was queued for measurement.

### Instrumentation and details of acquisitions

Analysis was performed using a Waters Acquity (UPLC i-Class; CSH, C18 reversed phase column, 2.1 × 100 mm, Particle size 1.7 μm) liquid chromatography system coupled with a Waters SYNAPT G2S*i* (Z-Spray, T-Wave Ion Mobility; TOF) mass spectrometer, previously modified to allow for ozonolysis reactions to be undertaken in the traveling-wave ion-mobility cell of the instrument^27^. Samples were prepared in 1.5 mL LC-MS vials with septum and kept in the autosampler of the LC system at 10 °C immediately prior to analysis. The column was kept at 60 °C during analysis. Liquid chromatography was performed with a linear gradient at a flow rate of 0.4 mL min^−1^. Mobile phase A is water with 0.1 % formic acid; mobile phase B is acetonitrile with 0.1% formic acid. The injection volume varies depending on the sample from 1 μL to 10 μL. Initially, the mobile phase was kept for 0.5 min at 20% B (80% A), then linearly increased to 90% B (16 min) and subsequently linearly increased to 100% B (17.35 min), held at 100% B until 20.35 min and finally reduced to 20% B until 21 min. The mass spectrometer was operated in positive mode, sensitivity mode, MS-mode and IMS-mode. The source temperature was set to 120 °C and the desolvation temperature to 550 °C. A capillary voltage of +2.5 kV was applied, with a sampling cone of 40 V and source offset set to 40 V. Cone gas was set to 100 L h^−1^, desolvation gas 900 L h^−1^ and the nebulizer was set to 6.5 bar. Gas controls were set to: trap 2.0 mL min^−1^, He-cell 180 mL min^−1^ and IMS 10 mL min^−1^. For data-independent acquisition, the quadrupole is operated in RF-only mode (MS method: MSe Continuum). For data-dependent acquisition, mass-selection was set to the defined *m/z* values (as per respective target list) with a tolerance of less than 4 Da (MS method: HD-DDA/Mobility Fast DDA; LM resolution: 5.0; HM resolution: 15.0; peak selection from “Include File List” (target list) within ±150 mDa and ± 0.15 s). For ozonolysis, the IMS travelling wave velocity and height were 650 ms^−1^ and 28 V, while the transfer travelling wave velocity and height were 1000 ms^−1^ and 2 V, respectively. The ion mobility cell is filled with a mixture of ozone, oxygen and nitrogen, instead of just nitrogen. Oxygen (10 psi) is converted to ozone using a high-concentration ozone generator (Ozone solutions, TG-40), generating 200–250 gm^−3^ of ozone in oxygen at a flow of 400 mL min^−1^. A portion of the ozone in oxygen mixture is then introduced into the IMS gas flow using a needle valve, such that the pressure in the IMS region is ~3 mbar. Excess ozone is catalytically destroyed using an unheated ozone destruct catalyst before being exhausted from the laboratory. Both ambient and in-situ produced ozone concentrations are monitored each by an ozone monitor (106-L and 106-H, respectively; 2B Technologies). A solution of 200 pg μL^−1^ leucine enkephalin in a mixture of 50% acetonitrile and 50% water with 0.1% formic acid is used as a Lockmass solution. Lockmass correction is performed upon loading of the raw LC–MS dataset into Skyline. Data is acquired at a scan time of 0.1 s. For acquisition of direct infusion data, the column was removed from the chromatography system and each sample was injected by the autosampler into a 50:50 mixture of acetonitrile/water with a 0.1% formic acid content (loop injection) at a flow rate of 0.4 mL min^−1^. Data is acquired for 2 min. After each injection, a needle wash was performed.

### General data analysis

Data were processed and visualized with custom-written python source code, Skyline, MassLynx, Microsoft Excel and OriginPro 9.1G. For the automated data analysis using the workflow presented here, template files and settings files provided within the associated git-hub repository are required^82^. An installation of Skyline MS (Skyline or Skyline-Daily, 20.2; 64-bit), Skyline Runner, as well as a current version of python (e.g., python 3.9.2; 64-bit; IDE: e.g., Visual Studio Code), including pandas, openpyxl, scipy, numpy, matplotlib, brainpy, requests, bs4, subprocess, statistics, csv and datetime on a Windows PC were used herein. For both the analysis of direct infusion ESI-MS and LC-OzID-MS(/MS) data the same four-letter-codes are used to describe the derivatization agent (charged head group). An overview of available abbreviations and derivatization agents is shown in Supplementary Fig. 1. The workflow can be applied to other derivatization agents without modification. In this case, a custom chosen four-letter-code and the sum formula of the associated head group needs to be entered. An overview of the stages of the analysis and acquisition steps as well as a detailed step-by-step protocol is shown in Supplementary Fig. 2 and the Supplementary Methods.

### Data analysis protocol for direct infusion by loop injection

The simultaneous detection of a mixture of positive charge-tagged fatty acids prevents ionization biases during electrospray ionization. The measurement, carried out without fragmentation or ozone-induced dissociation (OzID), reveals ratios of fatty acid isomer groups in a commercial standard mixture (Supplementary Note 1 and Supplementary Fig. 9). We introduce a python script for the automated analysis of the direct infusion MS data, which includes isotope corrections to account for varied numbers of ^13^C and other isotopes, as well as isobaric overlap of unsaturated fatty acids that differ by 2 Da in *m/z*, e.g., DPA (22:5) and DHA (22:6). The script performs a baseline subtraction, corrects *m/z* values and allows for subtraction of a process blank. Prior to running the script to quantify fatty acids, the acquired data is averaged over the full width at half maximum of the eluted peak of the loop injection. The resulting mass spectrum is copied into the respective input excel file. This is carried out analogously for the respective Process Blank. Upon starting the python script, the four-letter-code of the derivatization agent is requested, before values within the defined *m/z* range of the mass spectrum is read from the file into lists. First, the baseline is numerically calculated and subtracted from the spectrum. Any value that is negative after baseline correction is set to zero. Second, an *m/z* correction is performed: The peaks associated to the derivatized palmitic acid (16:0) and stearic acid (18:0) are located and the average deviation of the *m/z* value of their peak maxima from the theoretical *m/z* is calculated. The *m/z* values of the entire spectrum are corrected by the resulting shift. After isotopic corrections, the output excel file is written containing both original and processed spectra and relative quantities, from which absolute quantities are calculated using the internal standard.

### Data analysis protocol for data independent acquisition; UPLC-OzID-MS

The analysis of each LC-OzID-MS and LC-OzID-MS/MS dataset is controlled by windows batch files, which invoke instances of Skyline runner and custom python source code. The automated and user interactive steps in this procedure are described in detail in the Supplementary methods and Supplementary Fig. 2. Isomeric fixed-charge derivatized fatty acids elute at similar retention times in reversed-phase liquid chromatography, allowing predicted retention time ranges based on known fatty acid standards to be used as a criterion for identification. The first step in the automated data analysis workflow establishes such retention time ranges employing the observed retention time of palmitic and stearic acid. These saturated fatty acids are present in practically all biological samples, as they also constitute the main components of any process blank sample. The prediction is empirically based on observed retention times of analytical standards, but fatty acid structures are highly predictably correlated to their retention times, allowing extrapolation from observations using analytical standards to all related structures.

### Data analysis protocol for data dependent acquisition; UPLC-OzID-MS/MS

The analysis steps that follow the precursor analysis step (performed on the DIA dataset) are highly similar for both analysis of DIA and DDA datasets. The user is prompted to select one of three options. (I) Library based search, which triggers a search for double bond positions that are specified in an excel file; (II) de novo exhaustive search, which triggers a search for any double bond position that is chemically feasible, including conjugated fatty acids, but excluding allylic double bonds (i.e., chemically unstable allenes) or (III) streamlined analysis, which invokes a de novo exhaustive search for all fatty acids up to and including three double bonds, but carries out a library based search for all fatty acids with more than three double bonds. The library-based modes allow faster analysis either when the identity of fatty acid isomers in the sample is known, or when the dataset is to be probed for a defined set of fatty acid isomers. On the contrary, the analysis of highly complex fatty acid profiles with many unknown species is easier *via* the slower but more thorough de novo exhaustive search. Criteria for filtering the data are defined by the user through the Skyline template file (instrument mass resolving power) and through input requested by the workflow, such as intensity thresholds. Further criteria for retaining transitions are co-elution of OzID product ions and precursor in case of DIA analysis. Only relying on data-independent acquisition can lead to false positive and false negative identifications. Especially in the case of biological samples from mammalian metabolism, fatty acids with different *m/z* values may co-elute and produce OzID product ions that can theoretically arise from either precursor. Secondly, the extracted ion chromatogram of a potential OzID product ion in case of a low abundant species may only show an elevated baseline, instead of a clear chromatographic peak. These problems are overcome by the tandem MS acquisition, as mass selection of each precursor ensures that associated OzID product ions arise exclusively from the former and the signal-to-noise (*S*/*N*) ratio within the tandem mass spectrum is significantly improved over the data independent acquisition (Supplementary Figs. 16 and 17).

We further introduce a separate algorithm for the calculation of *S/N* ratios from mass spectral data extracted from Skyline (Supplementary Figs. 9 and 10). This algorithm can combine multiple individual spectra or use one and determines the number and average of OzID product peak intensities as well as the number and average peak intensity of noise peaks. Every peak *m/z* that is not within close proximity (defined *m/z* tolerance) of the precursor isotopic pattern or any *m/z* value that could arise from a possible OzID product is considered an eligible noise peak. The algorithm reads an excel input file that can contain hundreds of MS spectral data exported from Skyline (DDA), detects, which spectra to combine and which to compute separately and writes the *S*/*N* values for each species in an output excel file. Each eligible noise peak and each OzID product peak is numerically integrated, and the *S/N* value is calculated from the integrals and numbers of peaks according to Eq. 1. Signal is calculated as the sum of the numerical integrals of all OzID product peaks of the fatty acid in question, divided by the number of the OzID product peaks (two in case of a monounsaturated fatty acid, twelve in case of a hexaunsaturated acid). Noise is calculated as the sum of the numerical integrals of all eligible noise peaks divided by the number of the eligible noise peaks. Note that the precursor peak integral is not contained in this calculation as its intensity could be derived from a different regioisomer and only the OzID product ions in the data-dependent acquisition are directly diagnostic of the specific double bond isomer.

1 $$\frac{{S}}{{N}}=\frac{\sum \int ({{{{{\rm{OzID}}}}}}\; {{{{{\rm{peak}}}}}})/{n}_{{{{{{{\rm{OzID}}}}}}\; {{{{{\rm{peak}}}}}}}}}{\sum \int ({{{{{\rm{eligible}}}}}}\; {{{{{\rm{noise}}}}}}\; {{{{{\rm{peak}}}}}})/{n}_{{{{{{{\rm{eligible}}}}}}\; {{{{{\rm{noise}}}}}}\; {{{{{\rm{peak}}}}}}}}}$$

Relative quantification of isomeric fatty acids would be trivial if all isomers were completely separated from each other. Yet, reversed-phase UPLC does not fully resolve some isomeric species, which is often also true for gas-chromatographic methods. The conversion of double bonds to the characteristic product ions in in-situ OzID depends on the position of the double bond^26^. Therefore, the OzFAD workflow automatically creates an excel file for the deconvolution of the precursor chromatogram of each fatty acid group (Supplementary Fig. 6). The deconvolution parameters can easily be adjusted, and the saved document is the input for the following step of the automated analysis (Supplementary Fig. 7). Finally, the workflow makes tentative assignments of double bond configuration based on the order of elution and generates systematic names, fatty acid descriptors and retrieves LIPID MAPS IDs and common names from the LIPID MAPS database (Supplementary Table 1). The relative quantification is summarized both in a table and as a bar chart (Supplementary Fig. 8). A segmented bar chart showing a comparison of three replicates, or three fatty acid profiles can be generated with a separate python script from the output of the main workflow.

Fatty acid isomers are excluded from the analysis, if the feature is found to arise from over-oxidation of the isomer with the respective double bond in a position of one carbon closer to the carboxyl end of the fatty acid (Supplementary Note 1). The 37 mix fatty acid standard analysis (Supplementary Table 2) informs the degree of over-oxidation that leads to artefacts at the same retention time for the *n*−(*x* + 1) position at max. 1.5% for *cis* double bonds and max. 3% for *trans* double bonds (Supplementary Figs. 13–15). However, retention time shifts can ascertain that some features are not overoxidation artefacts (Supplementary Figs. 18–20). Fatty acids are also excluded from the analysis, if an amount exceeding 75% of the fatty acid in the sample is found in the associated Process Blank (Supplementary Note 2). Using the internal standard per-deuterated palmitic acid, the relative quantities of isomers were corrected by the amounts found in the associated Process Blank, where appropriate (In cases of between 1 and 75% of the fatty acid isomer that is present in the sample is also found in the Process Blank) (Supplementary Fig. 21).

## Supplementary information

Supplementary Information

Peer Review File

Description of Additional Supplementary Files

Supplementary Dataset 1

Supplementary Dataset 2

Supplementary Dataset 3

Supplementary Dataset 4

Supplementary Dataset 5

Supplementary Dataset 6

Supplementary Dataset 7

Supplementary Dataset 8

Supplementary Dataset 9

## Data Availability

The data that support the findings of this study (raw data and transition lists for viewing and analysis with Skyline version 21.1.0.278) are publicly available from QUT research data finder: 10.25912/RDF_1667992514317^83^. Source data are provided with this paper.

## Source data

Source Data

## Code availability

The OzFAD executable, python source code, windows batch files and associated files, including selected files and data for testing of individual steps of the workflow, are publicly available at: github.com/jphmenzel/jpmlipidomics. The latest release is also available via 10.5281/zenodo.7930133^82^.
