# Supplementary material for: Ozone-enabled fatty acid discovery reveals unexpected diversity in the human lipidome
Source: Nat Commun. 2023 Jul 4;14:3940. doi: 10.1038/s41467-023-39617-9 (PMC10319862; doi:10.1038/s41467-023-39617-9)
Supplement: Supplementary file 1 — Supplementary Information [file 41467_2023_39617_MOESM1_ESM.pdf]

Supplementary Information

for

Ozone-enabled fatty acid discovery reveals unexpected diversity in the human lipidome

Jan Philipp Menzel,[1,2,3,*] Reuben Young,[1,2,¢] Aurélie H. Benfield,[4] Julia S. Scott,[5,6] Puttandon Wongsomboon, [1,2] Lukáš Cudlman,[7,8] Josef Cvačka,[7,8] Lisa M. Butler,[5,6] Sónia T. Henriques,[4] Berwyck Poad,[1,2] Stephen Blanksby[1,2*]

[1]School of Chemistry and Physics, Queensland University of Technology, Brisbane, QLD 4000, Australia

[2]Centre for Materials Science, Queensland University of Technology, Brisbane, QLD 4000, Australia

[3]Centre for Data Science, Queensland University of Technology, Brisbane, QLD 4000, Australia

[4]School of Biomedical Sciences, Faculty of Health, Queensland University of Technology, Translational Research Institute, Brisbane, QLD 4102, Australia

[5]South Australian Immunogenomics Cancer Institute and Freemasons Centre for Male Health and Wellbeing, University of Adelaide, Adelaide, Australia

[6]South Australian Health and Medical Research Institute, Adelaide, Australia.

[7]Institute of Organic Chemistry and Biochemistry of the Czech Academy of Sciences, Flemingovo náměstí 542/2, 16600 Prague, Czech Republic.

[8]Department of Analytical Chemistry, Faculty of Science, Charles University, Prague 2, Czech Republic.

[*]Present address: Institute of Clinical Chemistry, Inselspital, Bern University Hospital, 3010 Bern, Switzerland.

[¢]Present address: Faculty of Science, Medicine and Health, School of Chemistry and Molecular Bioscience, Wollongong, Australia.

Table of contents

1. Supplementary methods ... 3

1.1 Materials ... 3

1.2 Data analysis with OzFAD ... 3

2.1 Details on semi-automated data analysis with OzFAD ... 4

2.2 Practical instructions on setting up the data analysis workflow OzFAD ... 5

2.3 Detailed step-by-step guide to data analysis with the OzFAD workflow ... 6

2.4 Algorithm for the determination of signal-to-noise values from OzID-MS/MS tandem mass spectra ... 13

2.5 Algorithm for the analysis of direct infusion mass spectra ... 14

2.6 Algorithm for the calculation of best estimates of absolute quantities based on LC-OzID-MS and direct infusion results ... 14

2.7 Algorithm for the visualization of replicate bar charts of relative abundances of isomers ... 14

2.8 Algorithm for the processing of data for calculation of P values and visualization as volcano plots and heatmaps ... 15

2.9 Algorithm for the creation of a Venn-Diagram inspired bar chart ... 15

3. Supplementary Notes ... 16

Supplementary Note 1 ... 16

3.1.1 Estimation of absolute quantities by direct infusion ESI-MS ... 17

3.1.2 LC-OzID-MS and LC-OzID-MS/MS analysis of the 37mix fatty acid standards ... 18

3.2 Supplementary Note 2 ... 23

3.3 Supplementary Note 3 ... 25

3.3.2 Orthogonal validation by GC-CACI-MS/MS ... 30

3.4 Supplementary Note 4 ... 31

3.5 Supplementary Note 5 ... 32

3.5.2 Orthogonal validation by LC-UVPD-MS/MS ... 35

3.6 Supplementary Note 6 ... 38

3.7 Supplementary Note 7 ... 39

3.7.1 MCF7 Breast Cancer - Human breast adenocarcinoma, metastatic ... 40

3.7.2 LNCaP Lymph node carcinoma of the Prostate - Human Prostatic Cancer Cells ... 41

3.7.3 LNCaP_SCD-1i Cancer of the Prostate - Human Prostatic Cancer Cells, SCD-1 inhibited ... 43

3.7.4 Comparison of relative abundance of fatty acid isomers between cancer cell lines and associated P values ... 44

3.7.5 Comparison of relative abundance of eicosa- docosa- and tetracosadienoic acids ... 46

4 Supplementary references ... 47

1. Supplementary methods

1.1 Materials

Acetonitrile (ACN, Fisher Chemical, Optima LC/MS suitable for UHPLC-UV), 0.1% formic acid in acetonitrile (Fisher Chemical, Optima LC/MS), *N,N*-dimethylformamide (DMF, Sigma-Aldrich, for HPLC, >99.9%), *N,N*-diisopropylethylamine (DIPEA, Sigma Aldrich, >99%), *N*-(3-dimethylaminopropyl)-*N'*-ethylcarbodiimide hydrochloride (EDC*HCl; Sigma Aldrich, 98%), 1-hydroxybenzotriazole hydrate (HOBt; Sigma Aldrich, >97.0%), n-pentane (Sigma-Aldrich, for HPLC, >99.0%), Metabolites in Frozen Human Plasma (Standard Reference Material 1950, National Institutes of Standards and Technology, NIST), methanol (MeOH, Fisher Chemical, Optima LC/MS), methyl *t*-butyl ether (MTBE, RCI Labscan, HPLC), tetrabutylammonium hydroxide solution (Sigma-Aldrich, 40 wt. % in H_2O), sodium chloride (NaCl, Sigma-Aldrich, SigmaUltra minimum 99.5%), Supelco 37 Component FAME Mix (certified reference material, TraceCERT in dichloromethane, varied conc., Sigma-Aldrich), and 0.1% formic acid in water (Fisher Chemical, Optima LC/MS) were used as received.

1.2 Data analysis with OzFAD

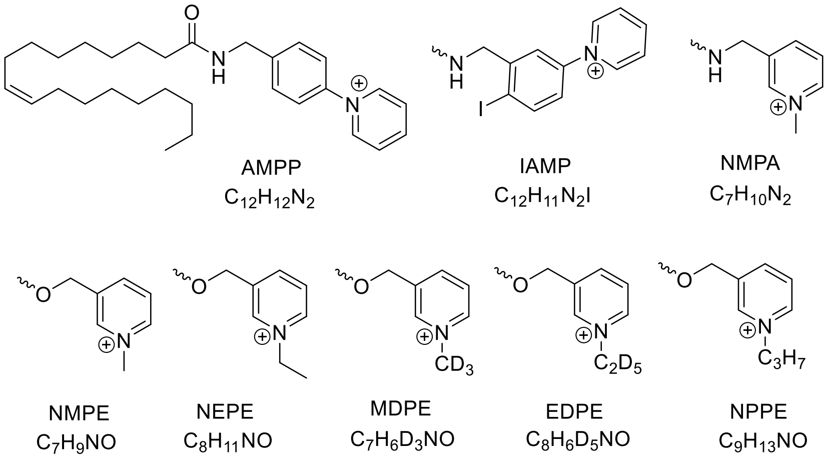

Supplementary Figure 1: Four-letter-codes of derivatization agents and sum formula of the respective head groups (bound to the fatty acid either via an amide or ester bond) shown as derivatized oleic acid (C18:1n-9). When either of the derivatization agents is used in the workflow, only the so-called "four-letter-code" shown here is required for running the computational data analysis, otherwise the sum formula of the head group is required. The selection of derivatization agents shown here is larger than the number used in the current work, as future studies may be carried out with different derivatization agents.[1-4] Each derivatization agent has some advantages ranging from commercial availability (AMPP) and opportunities for orthogonal photodissociation (UVPD) to analysis of deuterated derivatization agents for improved methods of relative quantification.

2.1 Details on semi-automated data analysis with OzFAD

A general overview of the workflow is displayed in Supplementary Figure 2.

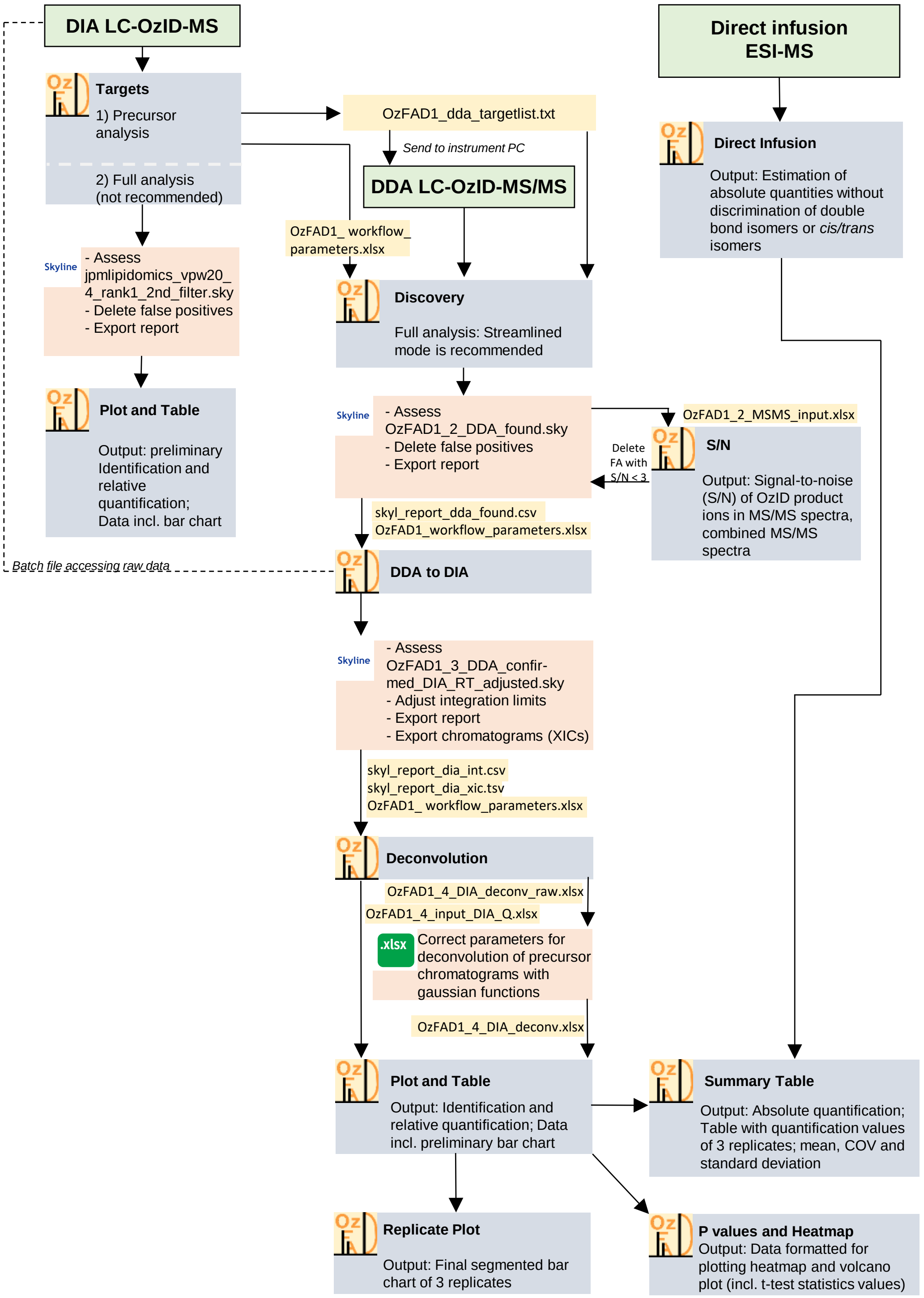

Supplementary Figure 2: Overview of the analysis workflow including instrumental analysis and all steps that require user intervention. Instrumental analysis steps are shown in green boxes, scripts that perform automated analysis steps are shown in grey boxes. Symbols indicate whether all steps within the respective analysis step are automated through the graphic user interface using batch and python scripts (which in turn control analysis steps using Skyline Runner), whether the user is required to carry out an assessment step in a Skyline file or whether the user is required to carry out manual review and adjustments in an excel file that was generated in the previous step.

2.2 Practical instructions on setting up the data analysis workflow OzFAD

The software for the data analysis workflow is publicly available on git-hub *via* github.com/jphmenzel/jpmlipidomics. All scripts and programs comprising OzFAD are accessible *via* a graphic user interface. This can be opened by two ways. First, the executable (latest release on git-hub (OzFAD1v3), the newest .exe file, e.g., OzFAD1v3_GUI_5.exe) starts the graphic user interface. Second, the python program OzFAD1v3_GUI_5.py starts the same graphic user interface. The workflow is written for use on a windows computer. Either way of accessing the software relies on Skyline being installed.

The folder structure of the workflow is defined within the git-hub repository, enabling quick setup. To analyze data with the OzFAD workflow, sufficient disk space is required locally (>10 GB). No instance of Skyline should be opened to prevent interference with Skyline Runner and raw files / folders are to be placed in the appropriate directories. The DIA LC-OzID-MS raw data file to be analyzed has to be copied into the folder *'DIA_current_LCMS_dataset'* and the DDA LC-OzID-MS/MS raw data file in the folder *'DDA_current_LCMS_dataset'*. There should at any time only be one dataset each in these folders, respectively.

In case of accessing the graphic user interface via the python program, several common python packages are required (pandas, openpyxl, scipy, brainpy, numpy, matplotlib, bs4, requests, csv, statistics, subprocess). These can be installed in *Visual Studio code* using pip with commands such as: 'pip install pandas', if not available within the local python version already. The Skyline runner executable is also available from the Skyline webpages. Log files, which are automatically generated in the folder OzFAD1.3/OzFAD1_black_box/workflow_log_files can be used to trace back processes executed by Skyline (Runner).

2.3 Detailed step-by-step guide to data analysis with the OzFAD workflow

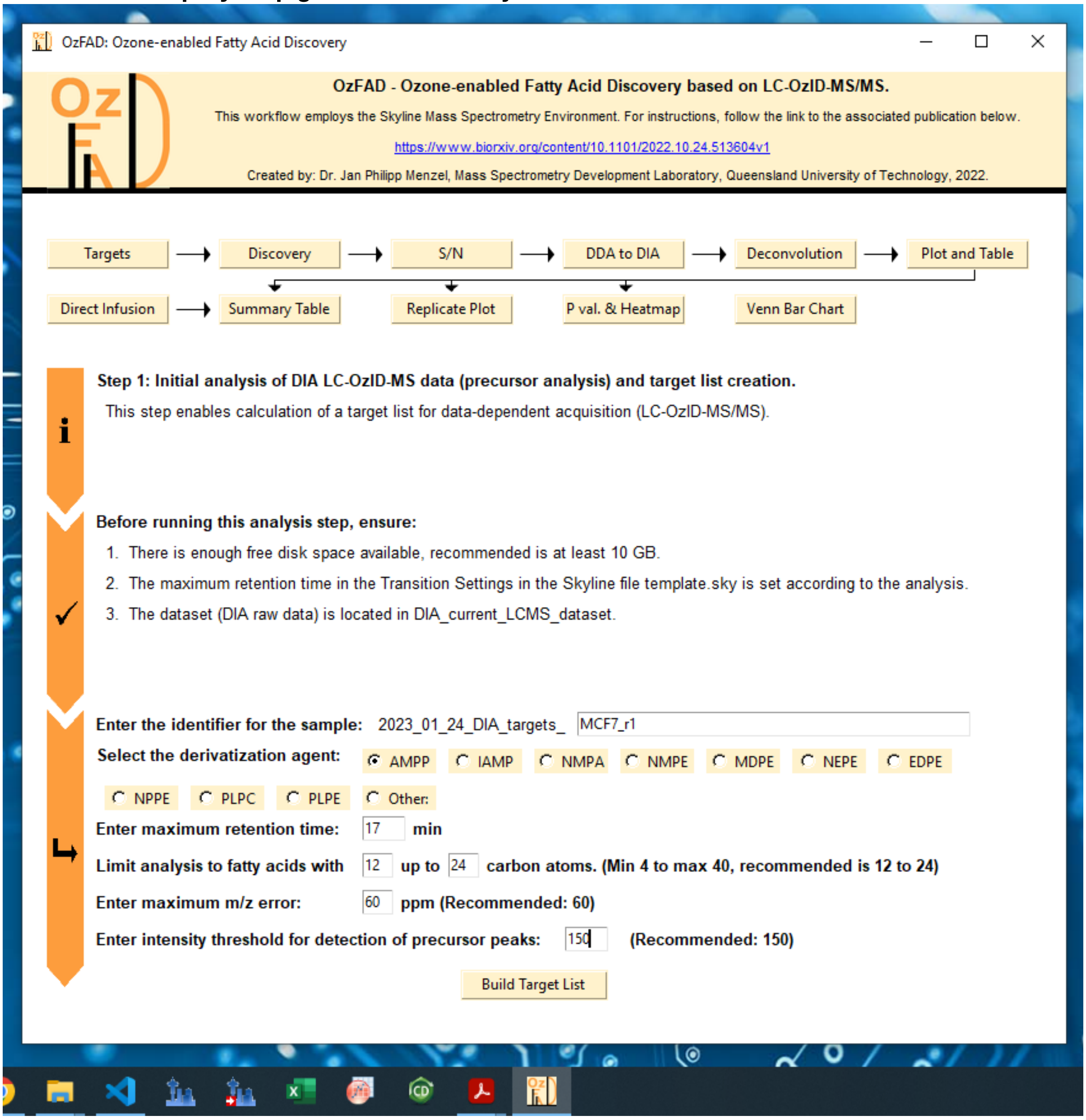

Supplementary Figure 3: Graphical user interface of OzFAD1.3 (screenshot). Upon starting the program, only the top part (all above black line as well as the eleven buttons and arrows connecting them) is visible. Selecting a button displays the instructions and input requests for each step of the workflow, respectively. If input of the derivatization agent is required, this can be selected via the radiobuttons shown. Selection of "Other:" prompts the user to enter a new four letter code as well as the sum formula of the new derivatization agent. The requirements (input files and raw data) are for each step of the workflow outlined in the graphical user interface. Each automated data analysis step is started by the button on the bottom of the page, respectively. During the run, a command window may pop up, displaying information on the progress of the workflow. Once completed, this command window will read "Press any key to continue…", indicating that this step of the workflow is complete, and the command window and the graphic user interface can be closed. The output of each step will appear in an automatically created folder in OzFAD1.3/OzFAD1_results/, which carries the folder name according to the identifier of the run of the workflow. The identifier in case of target list generation (similar to later steps involving Skyline) is the current date (updated by the workflow automatically), a description of the step of the workflow and the user input that identifies the sample being analysed. Each step can be carried out independently, provided that input files as requested are entered in the respective folder location.

1. To ensure that fatty acid isomers are reliably identified and do not originate from background (glassware or pipette tips), always analyze a Process Blank associated with each set of

samples in addition to the samples with the workflow. This step-by-step guide uses the datasets of the MCF7 cell line extract, replicate 1 to demonstrate the practical steps of the workflow.

2. Start the step “Targets” and enter input parameters as requested, see Supplementary Figure 5.
3. Rename target list and send to instrument PC. The separate raw target list is not to be used but serves as a reference to judge the target resampling algorithm if required.
4. Perform data-dependent acquisition (LC-OzID-MS/MS).
5. Copy DDA LC-OzID-MS/MS dataset into the respective folder.
6. Copy the target list generated in the previous step, named jpmlipidomics_dda_targetlist.txt into the folder OzFAD1. (For the purposes of demonstrating the steps in the workflow, a changed target list that is limited to fatty acids with chain length of 16 to 18 carbon atoms is used here.)
7. Copy the workflow parameters file generated in the previous step, named OzFAD1_workflow_parameters.xlsx into the folder OzFAD1. (An adapted file is used here with the same limitation (FA C16 - 18.)
8. Start the “Discovery” step and choose workflow analysis mode. Three options are available: Full discovery, streamlined and library based. The library-based search can be used, when a selected number of defined isomers should be identified quickly (e.g., analysis of a replicate sample of one that has previously been characterized with the full or streamlined discovery workflow). The streamlined analysis is significantly faster than the full discovery mode, as it limits the search for polyunsaturated fatty acids with four or more double bonds to the ones that are listed in the fatty acid library. This reflects the diversity of natural fatty acid samples as there are more likely unexpected novel mono-, bis- and tris unsaturated fatty acids than tetra-, penta- or hexaunsaturated fatty acid isomers.

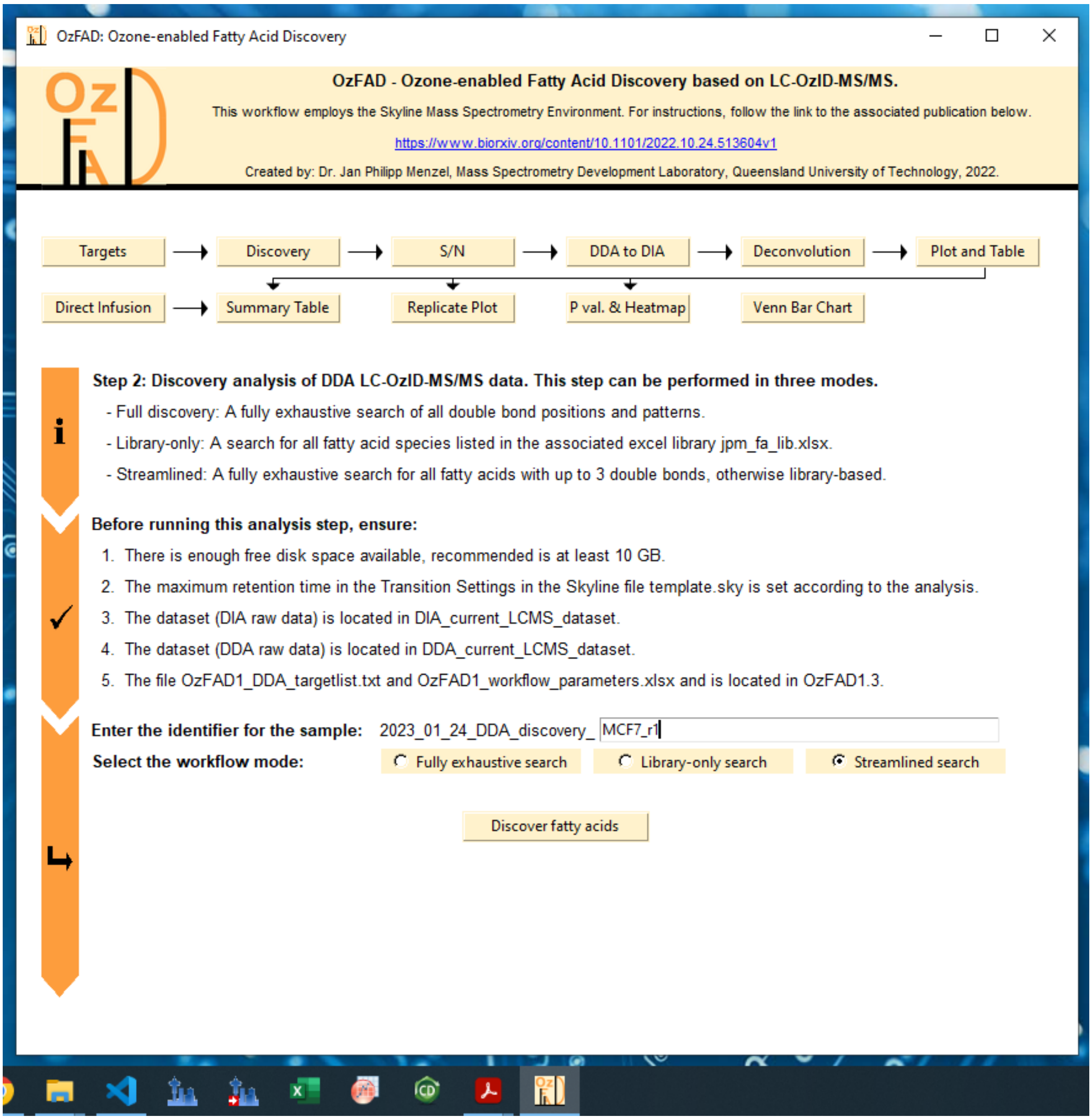

Supplementary Figure 4: Graphical user interface of OzFAD upon selection of the "Discovery" step of the workflow.

9. Open the Skyline file jpmlipidomics_dda_found.sky in the folder that was created with the name of the identifier as entered above within the folder OzFAD1_results.
10. Manually filter the list of fatty acid species and delete all that do not clearly correspond to an isomer identifiable by the presence of the precursor fatty acid *m/z* and the respective product *m/z* values. The view of the extracted ion chromatograms and the MS/MS spectra at the relevant retention times in Skyline helps to quickly identify, which species to keep and which to delete, see below. Additionally, delete those features that arise from over-oxidation. These features are identified by co-eluting OzID product ions of a double bond position one carbon closer to the methyl terminus that are not larger than 3% of the intensity of the latter.

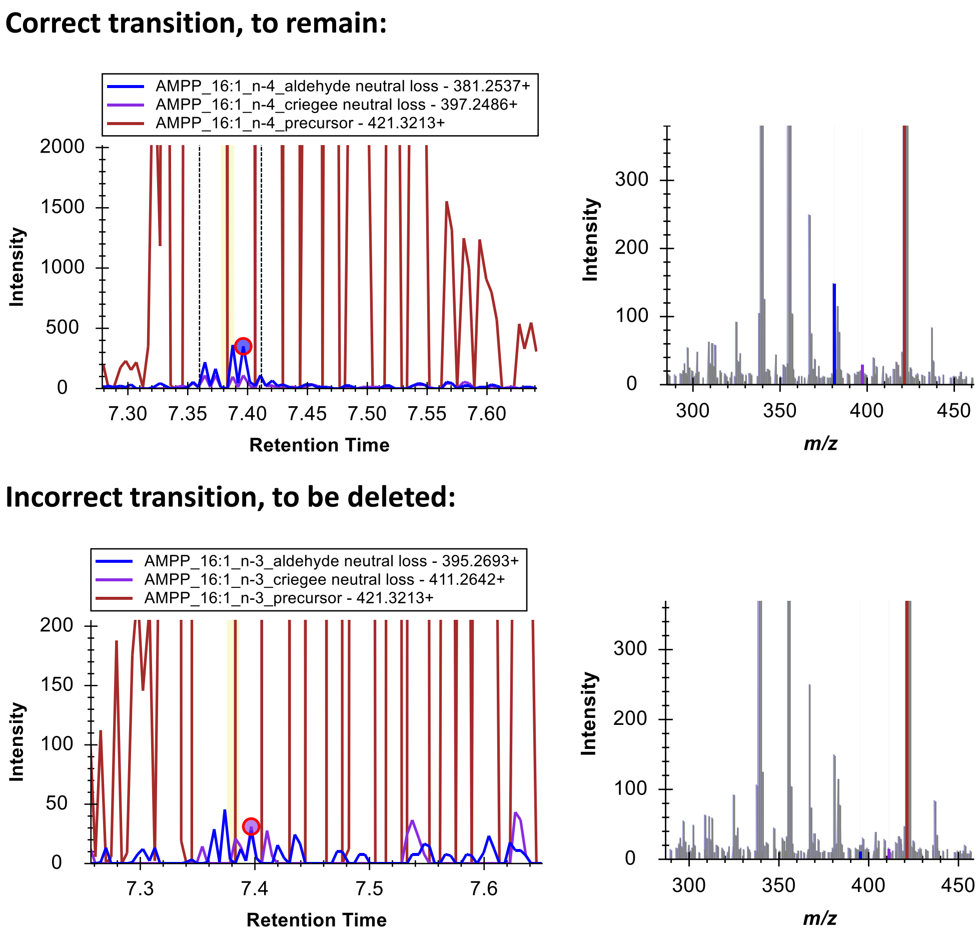

Supplementary Figure 5: Chromatograms (data-dependent acquisition) and tandem OzID mass spectra of a transition with OzID product ions above noise in comparison to a feature not significantly above noise.

11. For isomers that are low in abundance, export tandem mass spectra (OzID MS/MS; at least one spectrum, three recommended) from Skyline (from MS/MS view) as a Table and enter each into the input excel file for the S/N calculation step of the workflow. The signal-to-noise ratios for each species are then calculated. Keep only those species in Skyline with S/N > 10 (and S/N > 3 for those species that are to be tentatively identified, but not quantified). The results within this demonstration shows that FA 16:1*n*-3 is indeed not present above noise in MCF7 cell extracts as also seen in the Skyline view shown above. For this demonstration, all species other than hexadecenoic acids were deleted from the Skyline file. Export a Skyline report with the report template provided with this publication, the report csv file needs to be named skyl_report_dda_found.csv to enter the next stage of the workflow. The report can be changed into a transition list for reuse with Skyline on replicate datasets by changing the top row to the descriptions used in any other Skyline transition list generated by the workflow as well as deleting the last few columns while keeping explicit retention times and defining retention time windows.

12. Copy the previous file OzFAD1_workflow_parameter.xlsx and the file skyl_report_dda_found.csv into the folder OzFAD1 and carry out the step “DDA to DIA”.
13. Open the Skyline file jpmlipidomics_vpw20_6_DDA_confirmed_DIA_rt_shifted.sky in the folder associated with this run of the workflow (in OzFAD1_results). Adjust the integration borders of each isomer. Ideally, the center of the OzID chromatograms would align with the center between the integration limits and the integration limits would capture the entire OzID chromatogram peak width. Export a report (same report template as used earlier) into the file skyl_report_dia_int.csv and export chromatograms into the file skyl_report_dia_xic.tsv. Further,

carry out the analysis of the Process Blank equally to this step to identify, if some species need to be deleted from the sample due to their presence in both Sample and associated Process Blank in similar amounts.

14. Transfer the reports generated in step 14 as well as the file OzFAD1_workflow_parameters.xlsx into the folder OzFAD1 and carry out the “Deconvolution” step.
15. Open the file OzFAD1_4_DIA_deconv_raw.xlsx, go to sheet DC_16_1 (respectively all other sheets for each isomer group) and adjust the parameters for the fitting of gaussians to the precursor chromatogram (column B). The algorithm initially sets the ratios of the integrals of the gaussians according to the ratios of the OzID product ions (integrals shown in column E) as detected in the data-independent acquisition. The width of each gaussian within one isomer group is set to be equal, field B4 can be used to adjust these. Column G indicates, which isomer feature corresponds to the one with maximum intensity. The retention time position of each gaussian is set according to the retention times of each feature in Skyline. To conclude this analysis step, some height and position parameters for some gaussians need to be adjusted to match the sum of the gaussians to the precursor chromatograms. Often there are many features that coelute too closely and are present at widely differing intensities. Consequently, deconvolution can then not be carried out and the ratio of the OzID Integrals needs to be used to provide the best estimate of the contribution of the respective isomer to the precursor chromatogram. The graphs below represent the initially automatically generated plot and the graph with manually refined values. In this example, the deconvolution can only be done based on the gaussian fit for the species FA 16:1*n*-7, FA 16:1*n*-9 and FA 16:1*n*-10. Save the file as OzFAD1_4_DIA_deconv.xlsx.

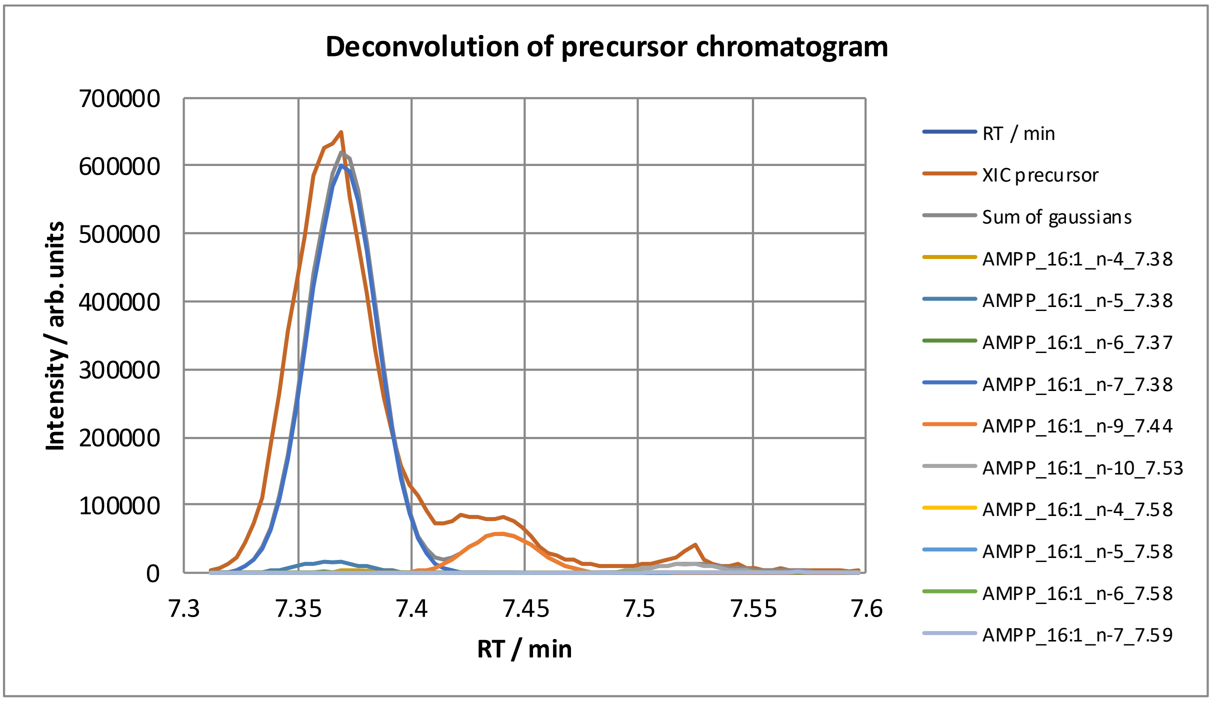

Supplementary Figure 6: Computationally generated suggested start values for deconvolution of an example precursor chromatogram (FA 16:1 in MCF7, replicate 1) with gaussian expressions representing the contribution of each fatty acid isomer.

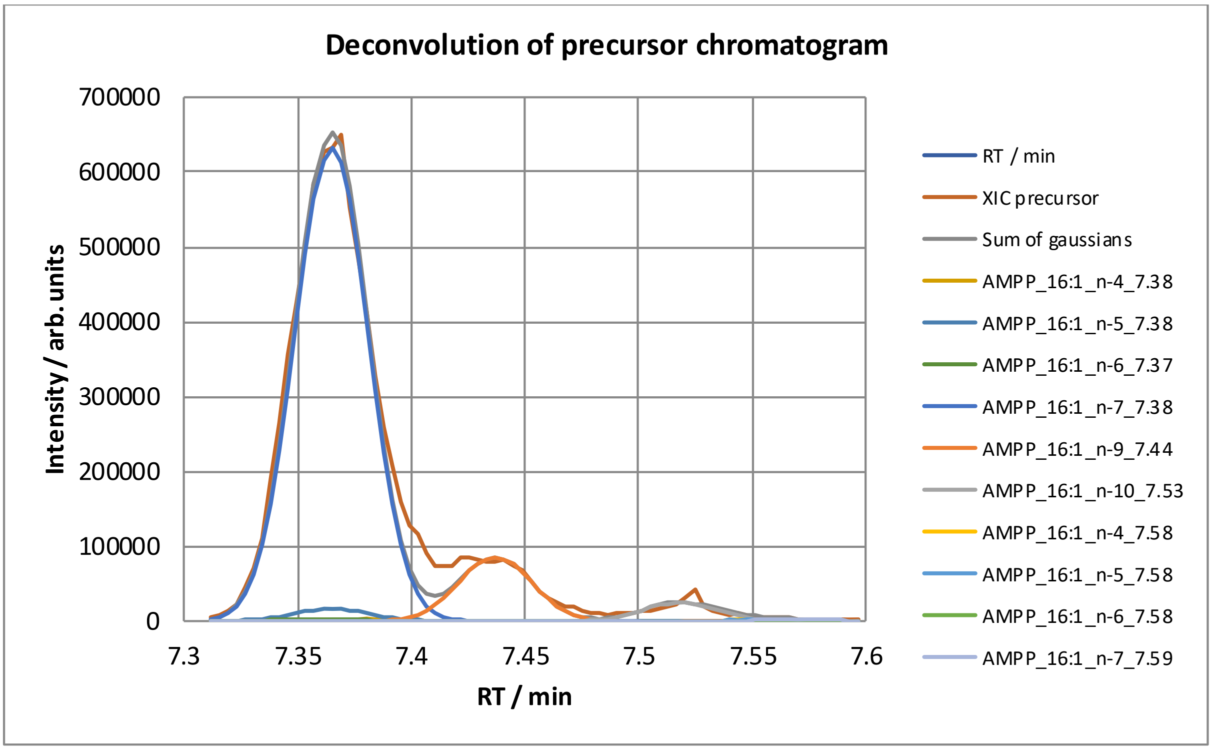

Supplementary Figure 7: Deconvolution of an example precursor chromatogram (FA 16:1 in MCF7, replicate 1) after manual refining of parameters with gaussians representing the contribution of each fatty acid isomer.

16. Transfer the files OzFAD1_4_DIA_deconv.xlsx (with adjusted parameters) and OzFAD1_4_input_DIA_Q.xlsx to the folder OzFAD1.2/OzFAD1_black_box/OzFAD1_py and run the "Plot and Table" step. Systematic names are automatically generated and the LipidMaps online database (LMSD) is searched for these systematic names. If an entry is found, the LipidMaps ID and common name of the fatty acid is retrieved. This database search is aided by the automated curation of an excel file "LIPID_MAPS_local_data.xlsx", which contains ID's of repeatedly identified fatty acids. If a fatty acid is not found in the local excel based database, the online database is searched. Fatty acids that are found in the workflow and are present in LipidMaps are automatically added to the local database if applicable.

The excel file that is produced contains the following bar chart and Table based on the analysis of hexadecenoic acids in MCF7 cells, replicate 1. The values are to be corrected for the Process Blank in case an isomer is present in the Process Blank in significant amounts. Sapienic acid is for example often detected in a Process Blank but may be present at much higher concentration in the sample under investigation.

Supplementary Table 1: Results summary of hexadecenoic acids in MCF7 cells, replicate 1.

Fatty acid (n-x)	FA shorthand	Systematic name	LipidMAPS ID	Common Name	RT / min	Relative isomer abundance / %
C16:1n-4	16:1(12Z)	12Z-hexadecenoic acid	Not found in LIPID MAPS.	_	7.37	0.89
C16:1n-5	16:1(11Z)	11Z-hexadecenoic acid	LMFA01030262	cis-Palmitvaccenic acid	7.37	2.1
C16:1n-6	16:1(10Z)	10Z-hexadecenoic acid	LMFA01030058	cis-10-palmitoleic acid	7.37	0.3
C16:1n-7	16:1(9Z)	9Z-hexadecenoic acid	LMFA01030056	cis-9-palmitoleic acid	7.37	82
C16:1n-9	16:1(7Z)	7Z-hexadecenoic acid	LMFA01030055	7Z-palmitoleic acid	7.39	11
C16:1n-10	16:1(6Z)	6Z-hexadecenoic acid	LMFA01030267	Sapienic acid	7.52	3
C16:1n-4_(E)	16:1(12E)	12E-hexadecenoic acid	Not found in LIPID MAPS.	_	7.52	0.17
C16:1n-5_(E)	16:1(11E)	11E-hexadecenoic acid	LMFA01030261	Lycopodic acid	7.52	0.13
C16:1n-6_(E)	16:1(10E)	10E-hexadecenoic acid	Not found in LIPID MAPS.	_	7.52	0.1
C16:1n-7_(E)	16:1(9E)	9E-hexadecenoic acid	LMFA01030057	trans-9-palmitoleic acid	7.54	0.33

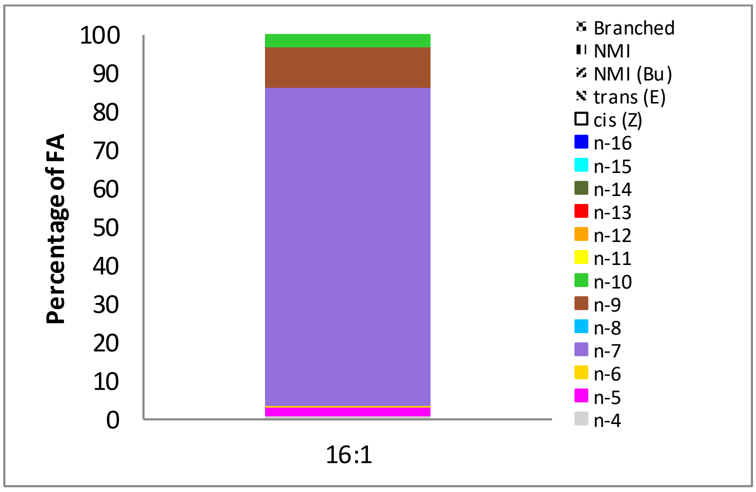

Supplementary Figure 8: Bar chart for relative isomer abundance of hexadecenoic acids in MCF7 cells, replicate 1. The abundance of *trans* fatty acids is so low that the respective segments in the bar appear not clearly visible.

2.4 Algorithm for the determination of signal-to-noise values from OzID-MS/MS tandem mass spectra

The process to determine S/N values from OzID-MS/MS spectra is visualized below using the example of AMPP derivatized 9Z,15Z-tetracosadienoic acid (C24:2n-9,15) in MCF7 cells.

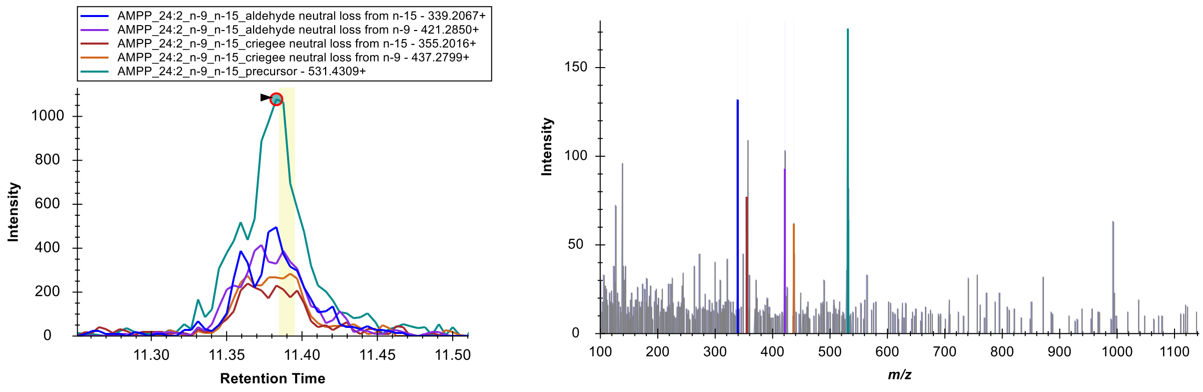

Supplementary Figure 9: Left: Extracted ion chromatogram of FA 24:2*n*-9,15 from the data-dependent acquisition (LC-OzID-MS/MS) of AMPP derivatized fatty acids in lipid extracts of MCF7 cells, replicate 1. Right: Associated OzID-MS/MS tandem mass spectrum at RT = 11.38 min. Displayed is each the visualization shown in Skyline, the legend and color coding from the chromatograms on the left applies to the mass spectrum on the right.

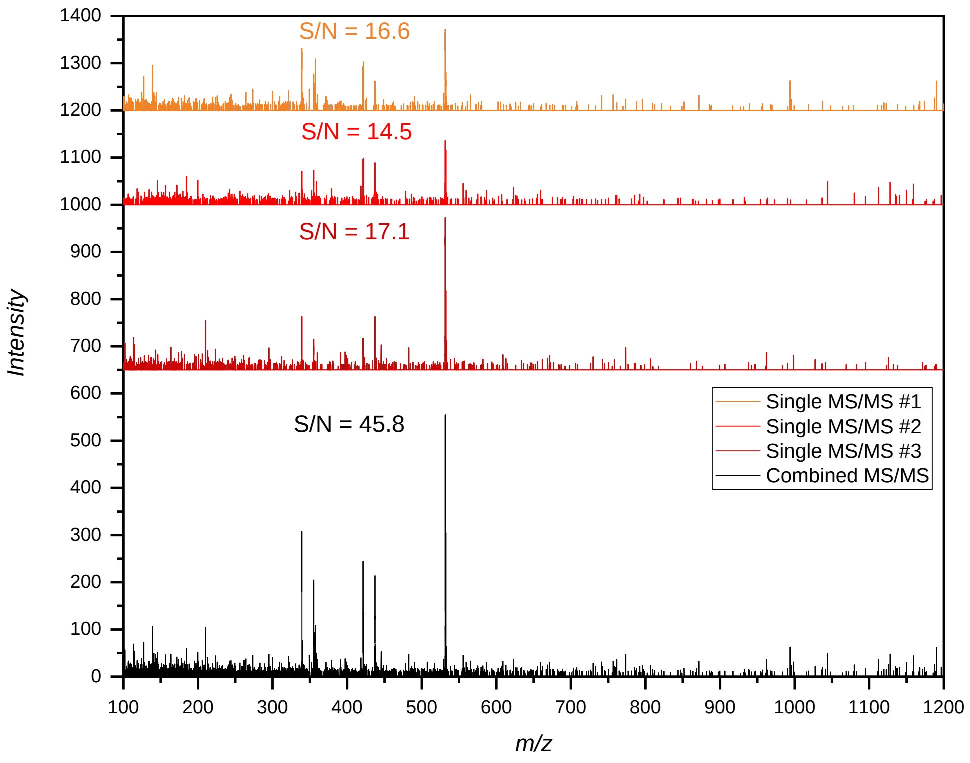

Supplementary Figure 10: To demonstrate the procedure for the calculation of S/N values from OzID-MS/MS spectra, three MS/MS spectra from the chromatographic peak shown in Supplementary Figure 4 were extracted and are displayed above the combined spectrum and S/N values for the fatty acid (C24:2n-9,15) are shown above each spectrum, respectively. The algorithm that calculates the S/N values reads an excel file containing the data of selected MS/MS spectra (data copied directly from Skyline to clipboard and into the excel input file). Multiple MS/MS spectra are combined by the algorithm and the combined spectra for each species are analyzed.

2.5 Algorithm for the analysis of direct infusion mass spectra
The structure of this algorithm is described in the methods section of this paper.

Initially, the spectrum is read from the input excel file. The background subtraction is performed by estimating the background / baseline as a hundred-point average curve of all datapoints of the spectrum, which are below an intensity of 100000. The baseline spectrum is then subtracted from the initial spectrum, with negative values set to zero. Next, the type of derivatization agent is used for the calculation of *m/z* values of palmitic and stearic acid. These two fatty acids are always present, even in a process blank. The respective peaks and their experimental *m/z* values are detected, allowing an *m/z* correction to be carried out based on the two *m/z* values. The FWHM of each principal ion of the two fatty acids is temporarily saved.

After background subtraction and *m/z* correction, the theoretical *m/z* values of the principal ions of all possible fatty acids (sum composition level) within the range of interest, e.g., 12:1, 12:2 …22:6 are calculated. For each, the peak of the principal ion is detected and, if found, integrated (numerical peak integration from neighboring minimum or zero-value before to the one after the detected peak). In case of a peak being found to be in close proximity to another peak, the integration of the peak area is limited to the *m/z* range spanning the theoretical *m/z* value ± the previously saved FWHM. This prevents falsely large peak integrals in cases of interference of contaminants with similar *m/z* to the analyte.

Subsequently, the integral values are corrected by the contribution of the +2 isotope of the respectively higher unsaturated fatty acid. Therefore, the theoretical fraction of the integral of the +2 ion from the isotopic pattern is calculated for all fatty acids with 6 double bonds. As no fatty acids with more than 6 double bonds are expected, the calculation begins with these. The explicit sum formula of each fatty acid with the derivatization agent is considered in each case. The integral of any determined peak representing a fatty acid with 6 double bonds is used to calculate the contribution of this isotopic pattern to the principal ion of any fatty acid with the same number of carbon atoms but containing one double bond less. The integral of the latter is then corrected by the calculated fraction. For example, the respective fraction of the +2 ion of the isotopic pattern of 22:6 is subtracted from the integral of the principal ion of 22:5. The corrections are iteratively applied, until the integrals of saturated fatty acids are corrected.

Finally, the corrected integral of principal ions are used to calculate the associated sum of the entire isotopic pattern representing the derivatized fatty acid species. The algorithm offers the option to compare the determined integral values for the fatty acids to the expected molar ratios of fatty acids in the FAME 37mix. The respective values can be displayed in bar charts to validate the accuracy of the quantification method including hydrolysis, derivatization and acquisition by direct infusion and data analysis with this algorithm. The results of this comparison are shown in Supplementary Figure 11.

2.6 Algorithm for the calculation of best estimates of absolute quantities based on LC-OzID-MS and direct infusion results
This algorithm serves to bring together the results of the LC-OzID-MS analysis and the direct infusion experiments of three replicates each. The results from the three direct infusion replicates are assembled in one excel file according to the template provided on git-hub. All input excel files are read by the program and the mean values, standard deviations and coefficients of variation for both the relative quantification based on LC-OzID-MS and the combined absolute quantities as means including standard deviation and coefficient of variation are determined and written into the output excel file (as well as individual values of all replicates). Values in column O (Rel. isomer quantity - mean and standard deviation) and P (Abs. isomer quantity – mean and standard deviation) are rounded according to the determined standard deviation. For example, if the raw values for the relative quantity of a species are 58.3078 ± 1.1283, this is displayed as 58 ± 1. Likewise, 0.02637 ± 0.00062 is displayed 0.026 ± 0.0006.

2.7 Algorithm for the visualization of replicate bar charts of relative abundances of isomers
This algorithm creates a segmented, stacked bar chart enabling the visualization of three instances (replicates or (means of) different samples) of relative quantities of fatty acids sorted by odd and even fatty acids. The chart is created with matplotlib in two steps. First the main chart is created, with the legend being created in a second step. The two components are merged to form the final graph. The size of axis labels can be controlled by editing line 14 of the associated python program

OzFAD1_6_Replicate_plot.py by changing fsize='14' to the desired value. The colours associated to the double bond positions can be changed by editing the list colourschemebarchart=['salmon', 'cornflowerblue', 'silver', 'magenta', 'gold', 'mediumpurple', 'deepskyblue', 'sienna', 'limegreen', 'yellow', 'orange', 'red', 'seagreen', 'cyan', 'blue'] in line 20.

2.8 Algorithm for the processing of data for calculation of P values and visualization as volcano plots and heatmaps

This algorithm reads the edited excel files of six samples (comparison of two types of sample with three replicates each) and calculates P values and fold changes. P values are calculated with the python package scipy ("t, p=stats.ttest_ind(cd1list, cd2list, equal_var=False)" with the respective values in the two lists). The final values are displayed in an excel output file in a way that they can be plotted as a volcano plot without further modification. The data are sorted by double bond positions to allow associating a colour to the groups of fatty acids as shown in Fig. 5.

2.9 Algorithm for the creation of a Venn-Diagram inspired bar chart

Venn diagrams are commonly used to visualize and compare quantities that may have several fractions of each quantity in common, for example the identification of fatty acids in human plasma by various methods. Several species are identified by all, some or only one method. A Venn diagram may be used to visualize performance of a method.

However, exact quantities are usually not proportional to the area of the Venn diagram that indicates common quantities. If a small number of cases or methods are being compared, an area-proportional Venn diagram can be constructed. In case of comparing more than 4 methods, the visualization can become crowded and tedious to decipher, if designed in an area-proportional way. Further, the human eye is naturally better at comparing the length of a bar, than the area of irregularly shaped patterns.

Thus, we present an algorithm that turns easy-to-assemble input data from an excel file into a Venn-diagram inspired bar chart, see Fig. 3d in the main paper. The algorithm builds a segmented bar chart by preserving key elements of a Venn diagram. The colours carry information on the identity of fatty acids across the different reports mentioned.

As an example: the grey bar at the bottom signifies the number of fatty acids that were found in each case. The blue-grey bar above the grey bar is present in all cases, except the study by Dodge et al., indicating that this number of fatty acids was identified in all cases except by this study. Likewise, colours are assigned to other groups of fatty acids, with the pink bar indicating that the respective number of fatty acids has only been identified herein, but not in any of the other contexts (which includes all literature and databases surveyed here).

As a consequence, segments in the bar chart with the same colour also have the same height, as they represent the same number of identified fatty acids. Colour coding is assigned automatically by the algorithm, leading to the most grey-tinted colours for the segments that are the most common across the methods and assigning the most vibrant colours to the segments that are common to the smallest number of methods or are unique to a method, highlighting differences between methods.

3. Supplementary Notes

Supplementary Note 1

The analytical standard SUPELCO FAME 37mix was used to benchmark the discovery workflow including the estimation of absolute quantities and relative quantities of fatty acids. 15 µL of the FAME 37mix solution dichloromethane were subjected to hydrolysis and AMPP derivatization equivalent to the other samples described in this work after evaporation of the dichloromethane under ambient conditions.

In general, *m/z* values of diagnostic OzID product ions are not displayed within this Supplementary Information but are available *via* the Skyline transition lists (column J) that can be accessed through research data finder together with the raw data supporting the findings of this study (https://doi.org/10.25912/RDF_1667992514317).

Supplementary Table 2: Absolute and relative quantities of fatty acids derived from the respective fatty acid methyl esters (FAME) in the 15 µL of the analytical standard solution.

Fatty acid species	Systematic Name	LIPID MAPS ID	n [nmol]
FA 14:1n-5	9Z-tetradecenoic acid	LMFA01030051	12.48
FA 15:1n-5	10Z-pentadecenoic acid	LMFA01030259	11.79
FA 16:1n-7	9Z-hexadecenoic acid	LMFA01030056	11.18
FA 17:1n-7	10Z-heptadecenoic acid	LMFA01030283	10.62
FA 18:1n-9	9Z-octadecenoic acid	LMFA01030002	20.24
FA 18:1n-9t	9E-octadecenoic acid	LMFA01030073	10.12
FA 20:1n-9	11Z-eicosenoic acid	LMFA01030085	9.24
FA 22:1n-9	13Z-docosenoic acid	LMFA01030089	8.51
FA 24:1n-9	15Z-tetracosenoic acid	LMFA01030092	7.88
FA 18:2n-6,9	9Z,12Z-octadecadienoic acid	LMFA01030120	10.19
FA 18:2n-6t,9t	9E,12E-octadecadienoic acid	LMFA01030123	10.19
FA 20:2n-6,9	11Z,14Z-eicosadienoic acid	LMFA01031043	9.30
FA 22:2n-6,9	13Z,16Z-docosadienoic acid	LMFA01030405	8.56
FA 18:3n-3,6,9	9Z,12Z,15Z-octadecatrienoic acid	LMFA01030152	10.26
FA 18:3n-6,9,12	6Z,9Z,12Z-octadecatrienoic acid	LMFA01030141	10.26
FA 20:3n-3,6,9	11Z,14Z,17Z-eicosatrienoic acid	LMFA01030378	9.36
FA 20:3n-6,9,12	8Z,11Z,14Z-eicosatrienoic acid	LMFA01030387	9.36
FA 20:4n-6,9,12,15	5Z,8Z,11Z,14Z-eicosatetraenoic acid	LMFA01030001	9.42
FA 20:5n-3,6,9,12,15	5Z,8Z,11Z,14Z,17Z-eicosapentaenoic acid	LMFA01030759	9.48
FA 22:6n-3,6,9,12,15,18	4Z,7Z,10Z,13Z,16Z,19Z-docosahexaenoic acid	LMFA01030185	8.76
FA 4:0	butyric acid	LMFA01010004	58.75
FA 6:0	hexanoic acid	LMFA01010006	46.09
FA 8:0	octanoic acid	LMFA01010008	37.92
FA 10:0	decanoic acid	LMFA01010010	32.21
FA 11:0	undecanoic acid	LMFA01010011	14.98
FA 12:0	dodecanoic acid	LMFA01010012	27.99
FA 13:0	tridecanoic acid	LMFA01010013	13.14
FA 14:0	tetradecanoic acid	LMFA01010014	24.75
FA 15:0	pentadecanoic acid	LMFA01010015	11.70
FA 16:0	hexadecanoic acid	LMFA01010001	33.28
FA 17:0	heptadecanoic acid	LMFA01010017	10.55
FA 18:0	octadecanoic acid	LMFA01010041	20.10
FA 20:0	eicosanoic acid	LMFA01010020	18.37
FA 21:0	heneicosanoic acid	LMFA01010021	8.81
FA 22:0	docosanoic acid	LMFA01010022	16.92
FA 23:0	tricosanoic acid	LMFA01010023	8.14
FA 24:0	tetracosanoic acid	LMFA01010024	15.68

3.1.1 Estimation of absolute quantities by direct infusion ESI-MS

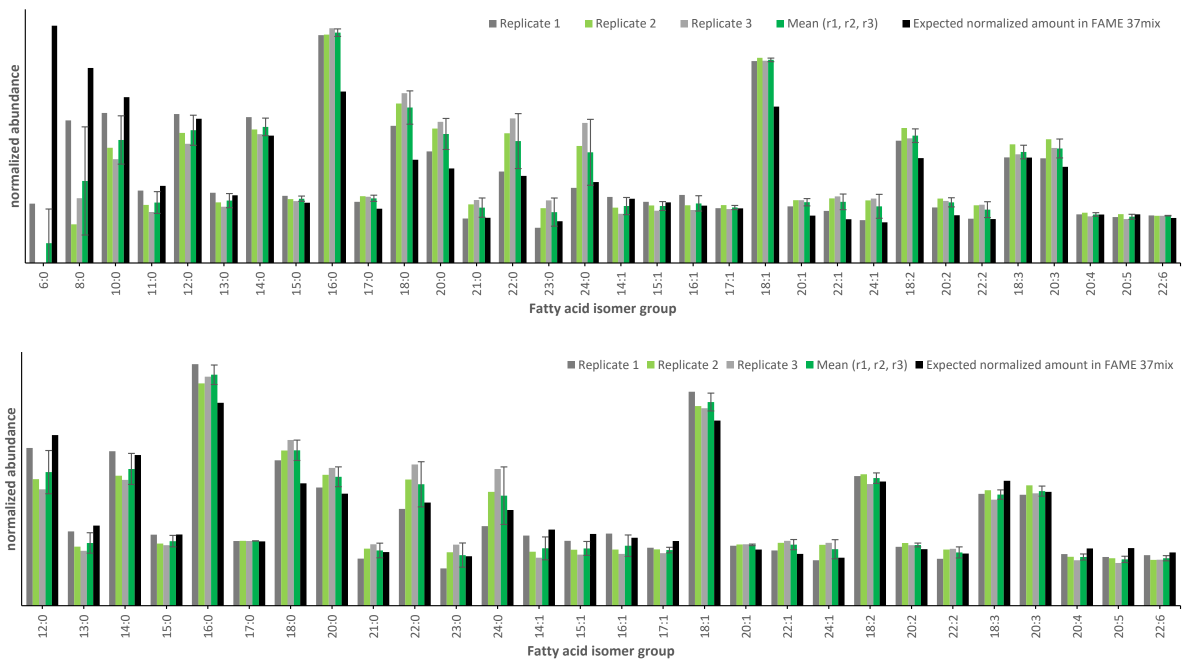

Supplementary Figure 11: Comparison of observed relative quantities of fatty acid isomer groups by direct-infusion ESI-MS to the expected quantities of the FAME 37mix standard fatty acid solution. Each distribution was normalized to match the sum of the fatty acids of the distributions. Note that short chain fatty acids are underrepresented in the detected values likely due to partial evaporation during sample preparation and the hydrolysis and derivatization procedure. For each graph, the individual values of the n=3 technical replicates as well as their mean (error bars represent standard deviation) and the expected normalized amounts are shown. Above: All fatty acids included in the FAME 37mix standard sample are shown. Below: Fatty acids shorter than 12 carbon atoms are excluded from the normalization.

The mean of the coefficient of variation of the absolute quantities observed for fatty acids in the 37mix standard from C12 to C24 (Supplementary Figure 10, lower bar chart) is 0.10 ± 0.07 (precision of quantitation – comparison of different fatty acid isomer groups – on average 10% of the determined value). The mean accuracy of quantification (mean absolute deviation between observed and expected normalized quantity divided by the expected normalized quantity) for the same fatty acid isomer groups is 15% ± 7%. Thus, quantities reported herein can serve as an approximate measure of the ratios and absolute values of fatty acid isomer groups present in the sample.

3.1.2 LC-OzID-MS and LC-OzID-MS/MS analysis of the 37mix fatty acid standards

In reversed-phase liquid chromatography, retention times are highly indicative of the structure of the lipophilic portion of a lipid or derivatized fatty acid. Retention time prediction has been used to improve lipidomics data analysis workflows[5] and is also a key initial step in the OzFAD workflow.

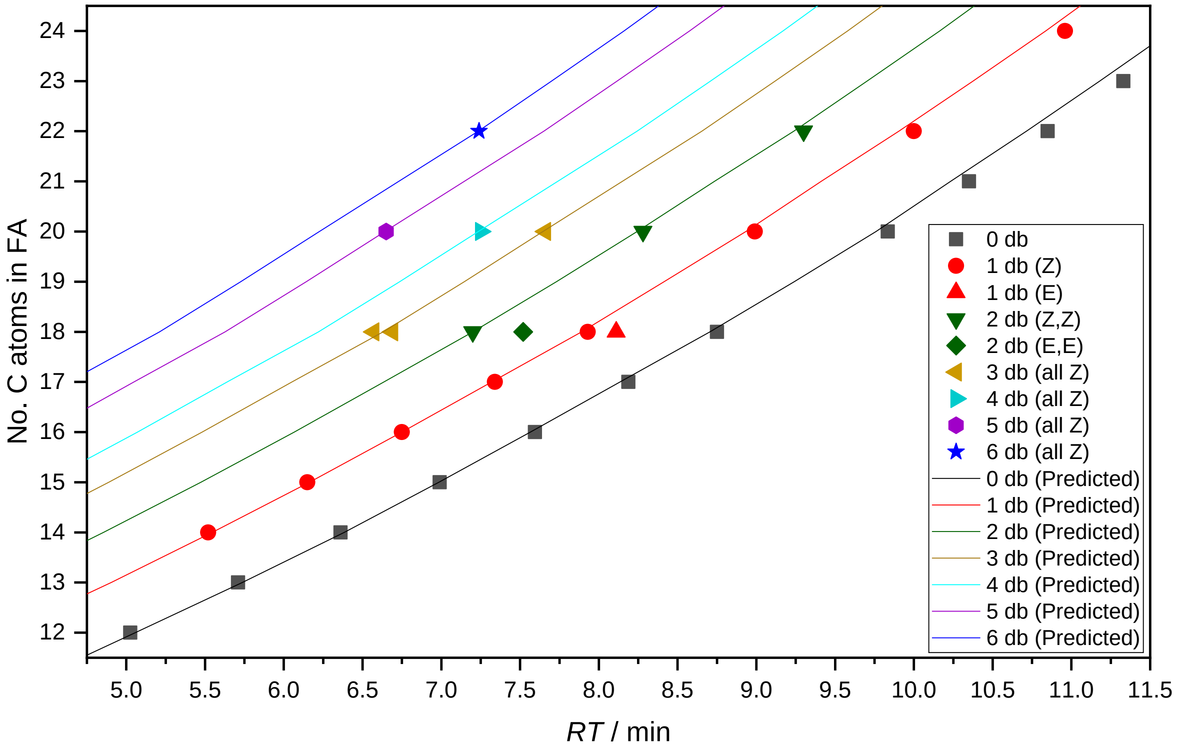

Supplementary Figure 12: Retention times of AMPP derivatized fatty acids of the 37mix standard sample and predicted / extrapolated retention times of fatty acids with varied equivalent chain lengths and number of double bonds. The prediction / extrapolation method is empirically based on the elution of the fatty acids in this 37mix sample but applies as a general retention time range prediction with an estimated error <15% for all fatty acids. Generally, the retention time of palmitic and stearic acid are the input values for the retention time range prediction that is used within the workflow to limit the search for chromatographic features that represent fatty acid isomers. The tolerance of ± 15% of the RT value or ± 1 min, whichever is larger, is used to allow detection of branched and trans fatty acid isomers, which are chromatographically shifted.

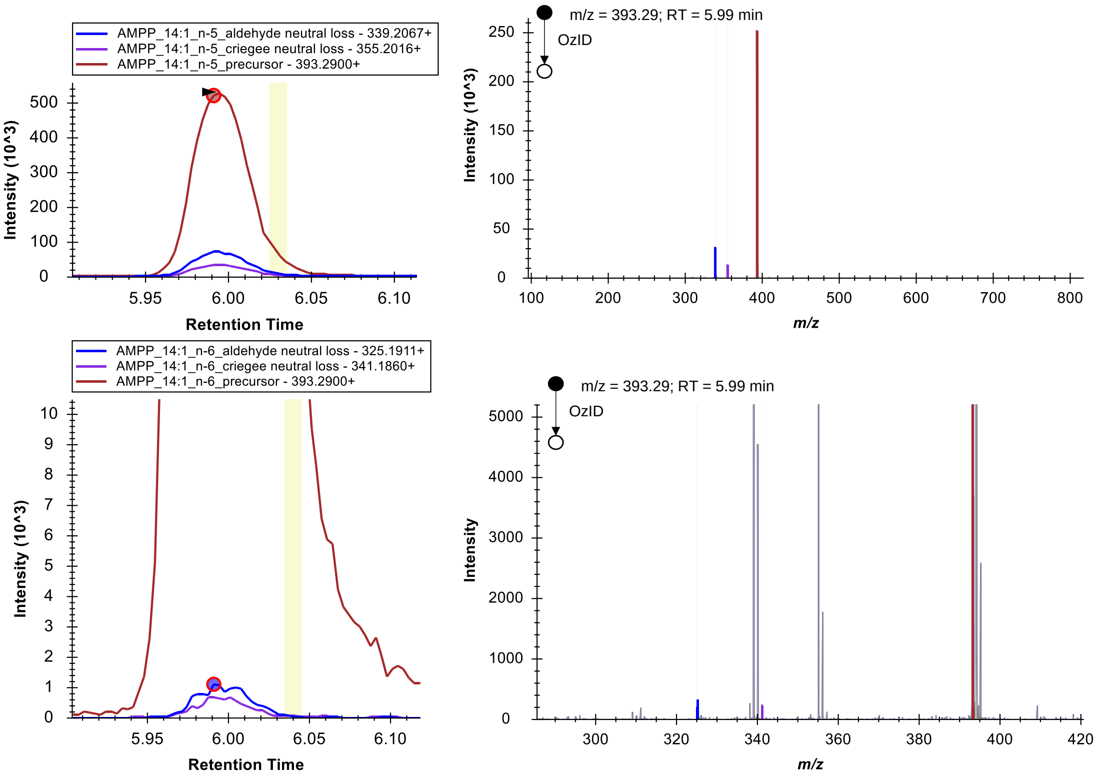

Supplementary Figure 13: Chromatograms and mass spectra of the data-dependent acquisition of the 37mix standard. Apart from the expected OzID product ions for FA 14:1*n*-5, a small degree of over-oxidation is observable as product ions with equal masses to the OzID product ions of the *n*-6 position.

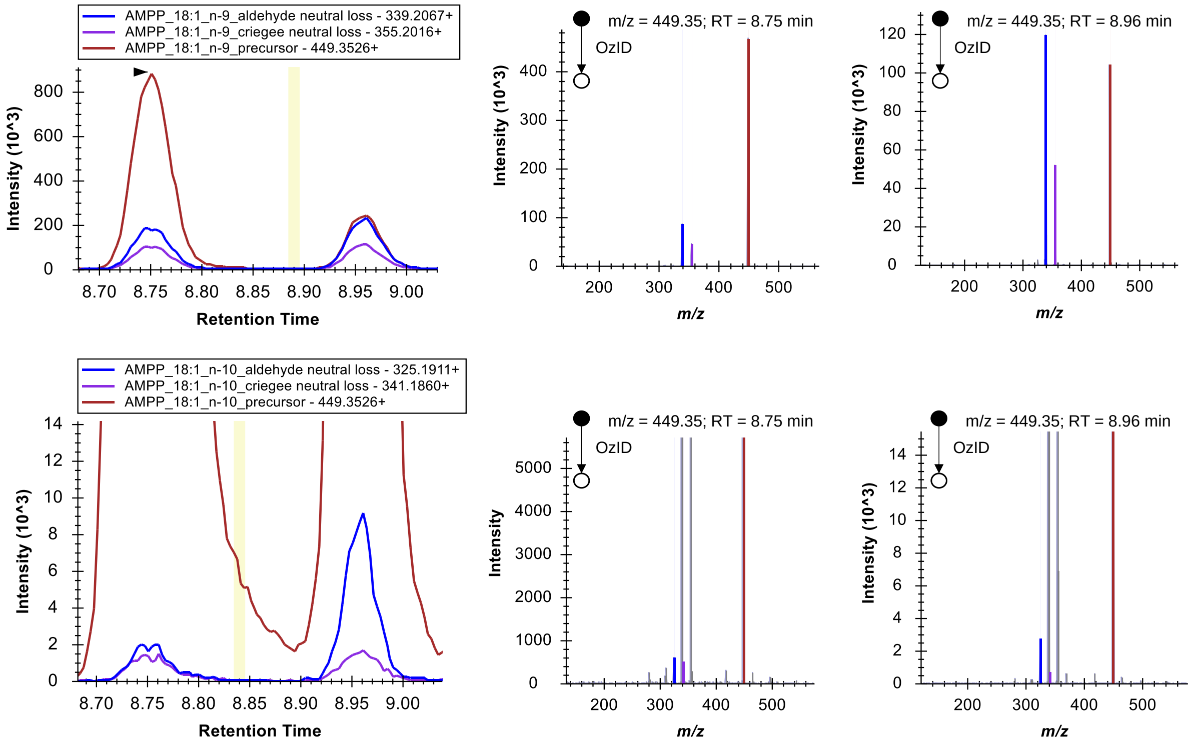

Supplementary Figure 14: Chromatograms and mass spectra of the data-dependent acquisition of the 37mix standard. Apart from the expected OzID product ions for FA 18:1*n*-9, a small degree of over-oxidation is observable as product ions with equal masses to the OzID product ions of the *n*-10 position.

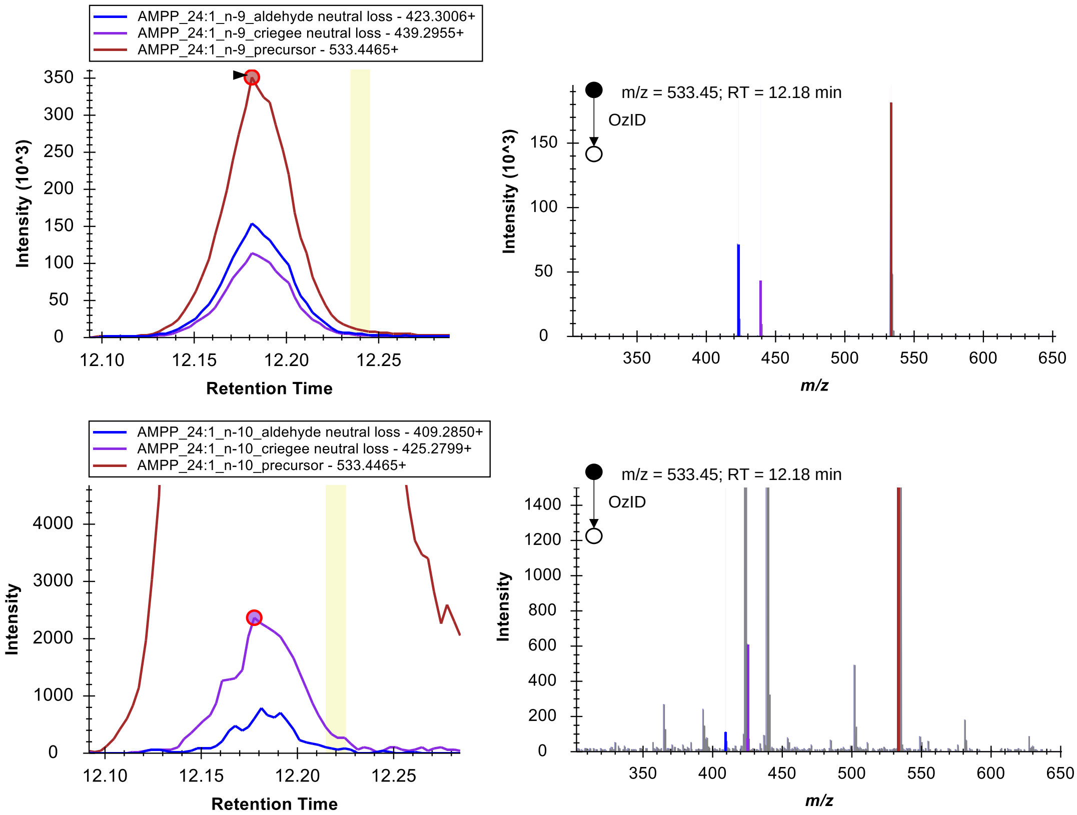

Supplementary Figure 15: Chromatograms and mass spectra of the data-dependent acquisition of the 37mix standard. Apart from the expected OzID product ions for FA 24:1*n*-9, a small degree of over-oxidation is observable as product ions with equal masses to the OzID product ions of the *n*-10 position. Note that due to the higher number of carbon atoms the +2 Isotope of the aldehyde product ion arising from ozonolysis on the n-9 position is isobaric with the criegee ion arising from over-oxidation.

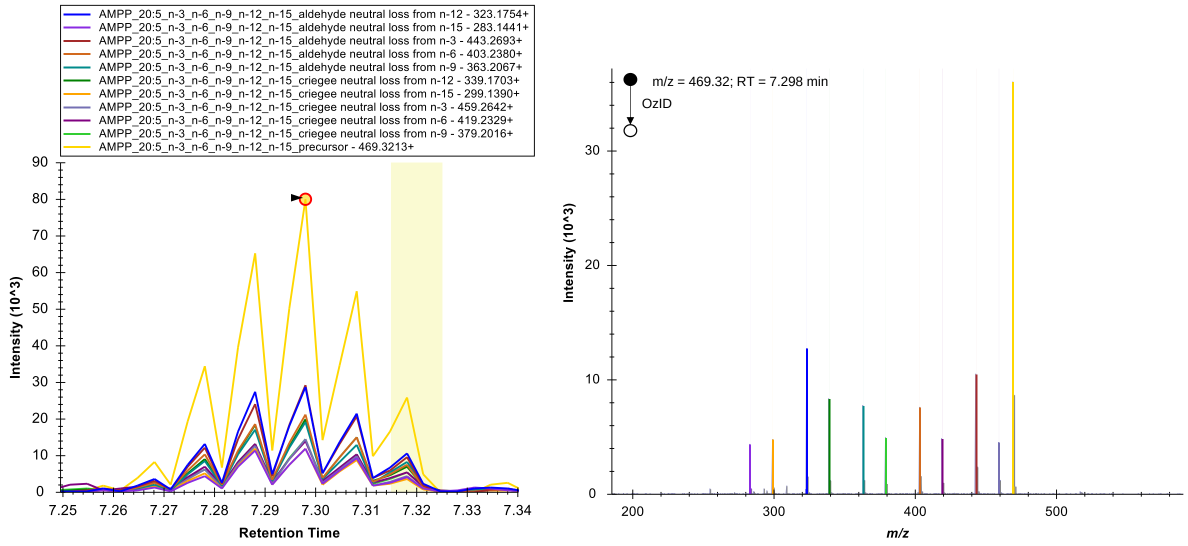

Supplementary Figure 16: Chromatograms and mass spectra of the data-dependent acquisition of the 37mix standard, showing FA 20:5*n*-3,6,9,12,15 (EPA).

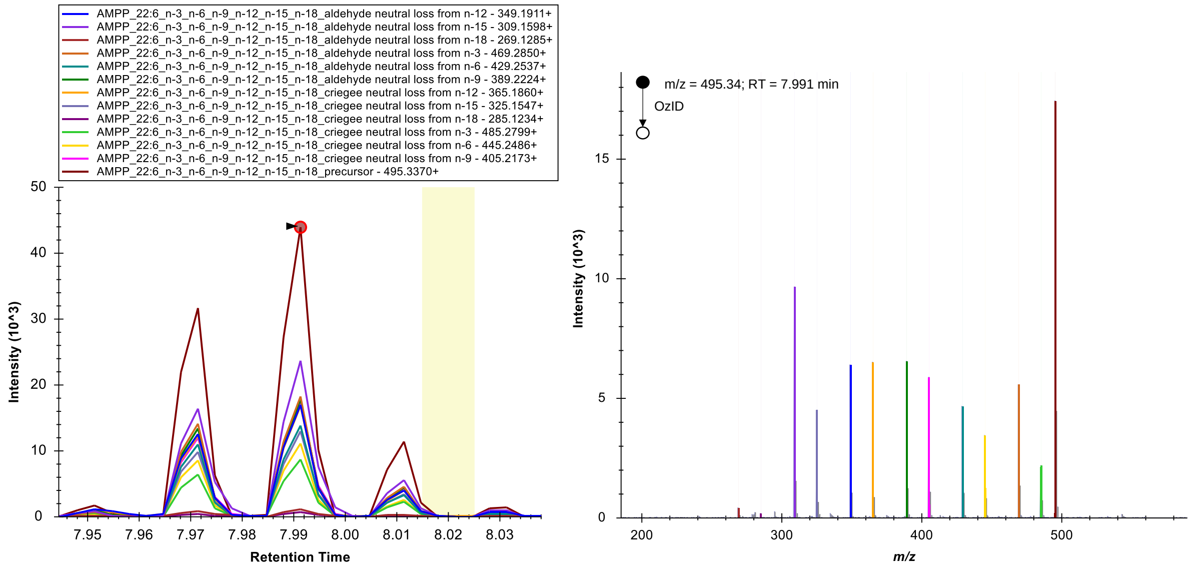

Supplementary Figure 17: Chromatograms and mass spectra of the data-dependent acquisition of the 37mix standard, showing FA 22:6*n*-3,6,9,12,15,18 (DHA). Note that ozone-induced dissociation from the Δ4 position (*n*-18) of the AMPP derivatized fatty acid appears to be drastically reduced in comparison to the other double bond positions.

Supplementary Table 3: Degree of over-oxidation for monounsaturated fatty acids in the 37mix standard. The mean percentage of over-oxidation for monounsaturated *cis* fatty acids is 1.24, while the degree of over-oxidation is 2.84 for *trans* FA 18:1*n*-9. Values were calculated from the sums of OzID product ion peak integrals from the data-dependent acquisition. Only fatty acid isomers were selected that featured an uninterrupted acquisition of the full peak.

FA	Over-oxidation / %
FA 14:1*n*-5	1.51
FA 15:1*n*-5	1.44
FA 18:1*n*-9	1.22
FA 18:1*n*-9t	2.84
FA 20:1*n*-9	1.16
FA 22:1*n*-9	0.99
FA 24:1*n*-9	1.13

All fatty acid isomer identifications in this work were validated to not be false positive discoveries based on the observed over-oxidation effect. For example, Supplementary Figure 17 shows the retention time shift between FA 19:1*n*-10 and FA 19:1*n*-11 in pooled human plasma, refer to Fig. 3 in the main paper, which evidences, apart from the observation of the ratio of the two fatty acids ($I_{C19:1n-1}$)/($I_{C19:1n-11}$+$I_{C19:1n-10}$) = 2%) that FA 19:1*n*-11 is a correctly identified isomer. Features that were excluded from the analysis as potential over-oxidation artefacts include FA 16:1*n*-8 *cis,*[6] FA 20:1*n*-10 *cis*,[7] and FA 24:1*n*-10 *cis* in NIST 1950 human plasma.

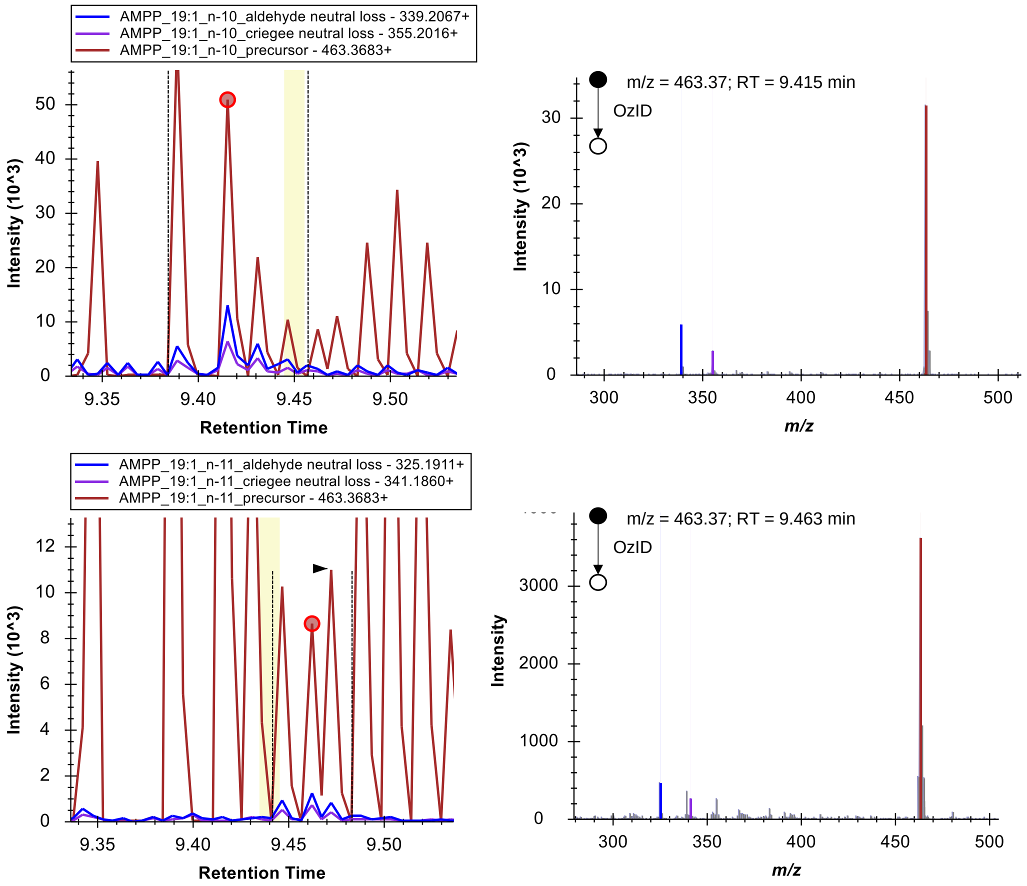

Supplementary Figure 18: Extracted ion chromatograms (data-dependent acquisition) and OzID-MS/MS spectra of FA 19:1*n*-10 (top chromatograms and associated MS/MS spectrum) and FA 19:1*n*-11 (bottom spectra) in NIST 1950 standard reference material (pooled human plasma).

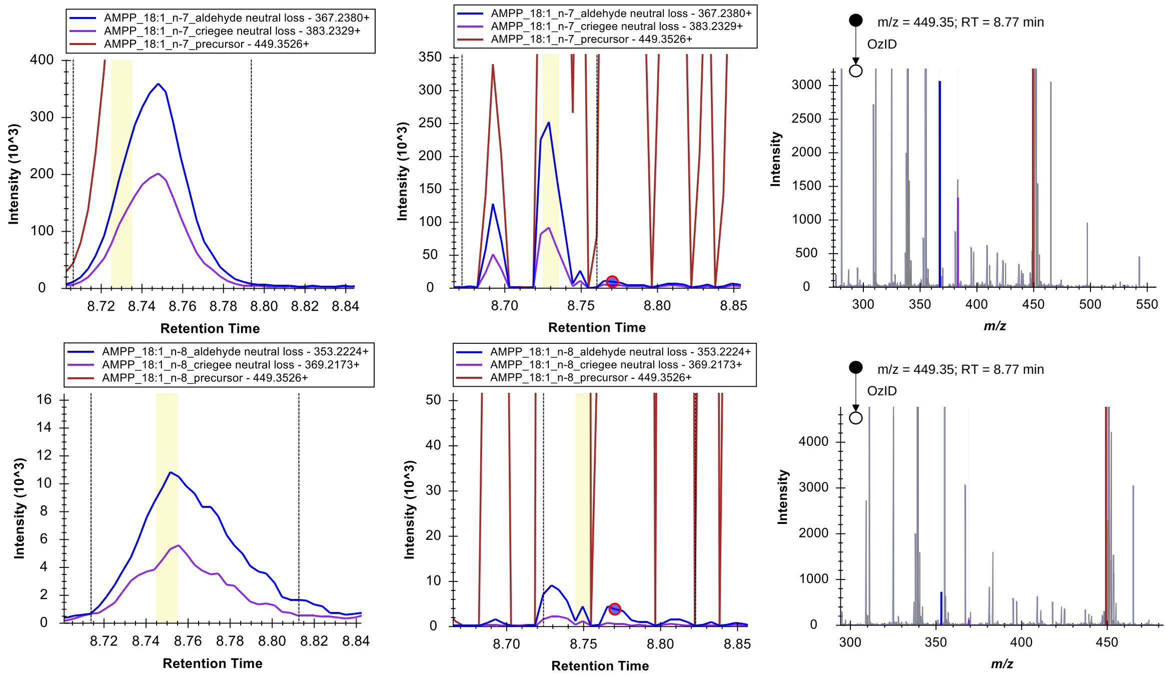

Supplementary Figure 19: Extracted ion chromatograms (left: data-independent acquisition; middle and right: data-dependent acquisition) and OzID-MS/MS spectra of FA 18:1*n*-7 *cis* (top chromatograms and associated MS/MS spectrum) and FA 18:1*n*-8 *cis* (bottom spectra) in NIST 1950 standard reference material (pooled human plasma). The retention time shift and the ratio of OzID product ion abundance of FA 18:1*n*-8 *cis* and FA 18:1*n*-7

cis at 8.77 min show that the feature representing FA 18:1*n*-8 *cis* is only partly caused by over-oxidation of FA 18:1*n*-7 *cis*.

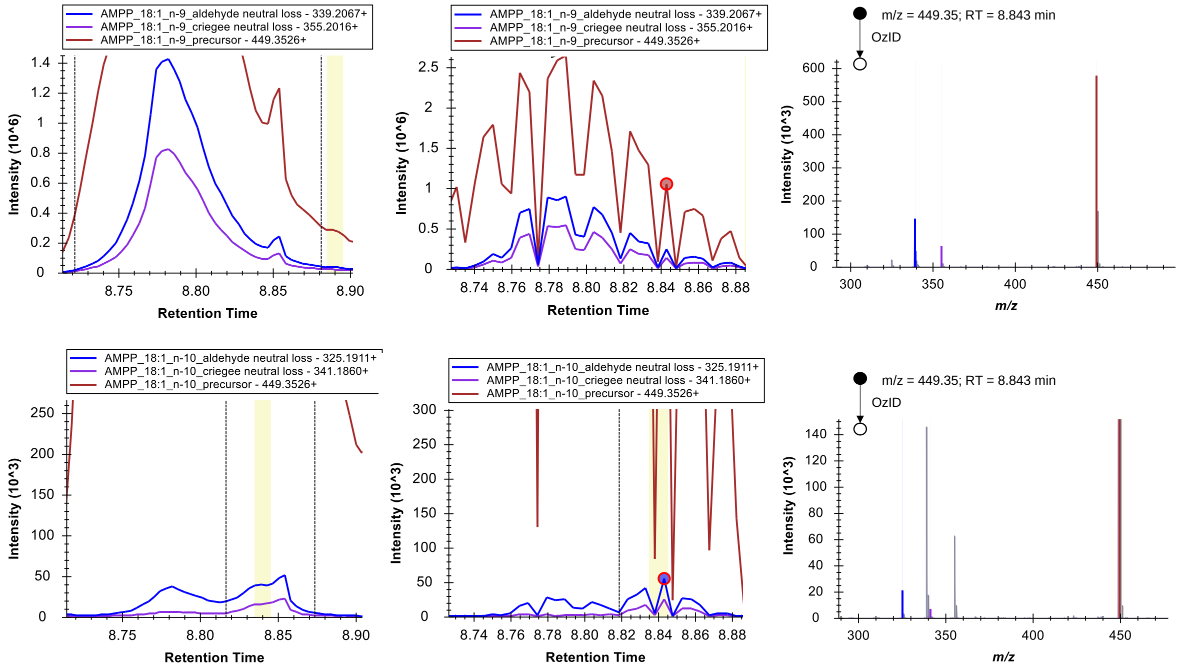

Supplementary Figure 20: Extracted ion chromatograms (left: data-independent acquisition; middle and right: data-dependent acquisition) and OzID-MS/MS spectra of FA 18:1n-9 *cis* (top chromatograms and associated MS/MS spectrum) and FA 18:1*n*-10 *cis* (bottom spectra) in NIST 1950 standard reference material (pooled human plasma). The retention time shift and the ratio of OzID product ion abundance of FA 18:1*n*-9 *cis* and FA 18:1*n*-10 *cis* at 8.843 min show that the feature at RT = 8.85 min representing FA 18:1*n*-10 *cis* is not caused by over-oxidation of FA 18:1*n*-9 *cis*.

3.2 Supplementary Note 2

Each lipid extraction and derivatization was accompanied by a process blank, a blank sample containing an equal amount of internal standard as the biological samples. The process blank is treated equally to the samples being taken through the workflow. In each process blank we observed a subset of small amounts of fatty acids, usually palmitic acid, stearic acid, erucic and oleic acid, among very minor amounts of other fatty acid species. We found that no method of cleaning glassware and tools could completely suppress the detection of remaining minor amounts of such fatty acids. Therefore, the respective Process blank is always acquired alongside the sample and a subtraction of the amounts of the species that are present at notable amounts relative to the biological sample is performed. One possible source of erucic acid appears to be the plastics additive erucamide,[8] possibly introduced into the workflow through the use of plastic tips of Eppendorf pipettes. A characteristic peak at the *m/z* value of erucamide is observed in the Process Blank of for example human plasma, as well as a significant amount of the derivatized erucic acid. The latter is observed in the direct infusion, the LC-OzID-MS and LC-OzID-MS/MS acquisitions.

Regarding the analysis of human plasma, all docosenoic acids (22:1) were therefore excluded from the analysis. Further, the fatty acids FA 14:1*n*-8 *cis*[9] and FA 15:1*n*-9 *cis*[9] were excluded from the analysis, as similar amounts of these species were observed in the associated Process Blank. The relative amounts of several other species was adjusted by Process Blank subtraction. Example spectra for the Process Blank subtraction of Sapienic acid FA 16:1*n*-10 *cis* are shown in Supplementary Figure 20.

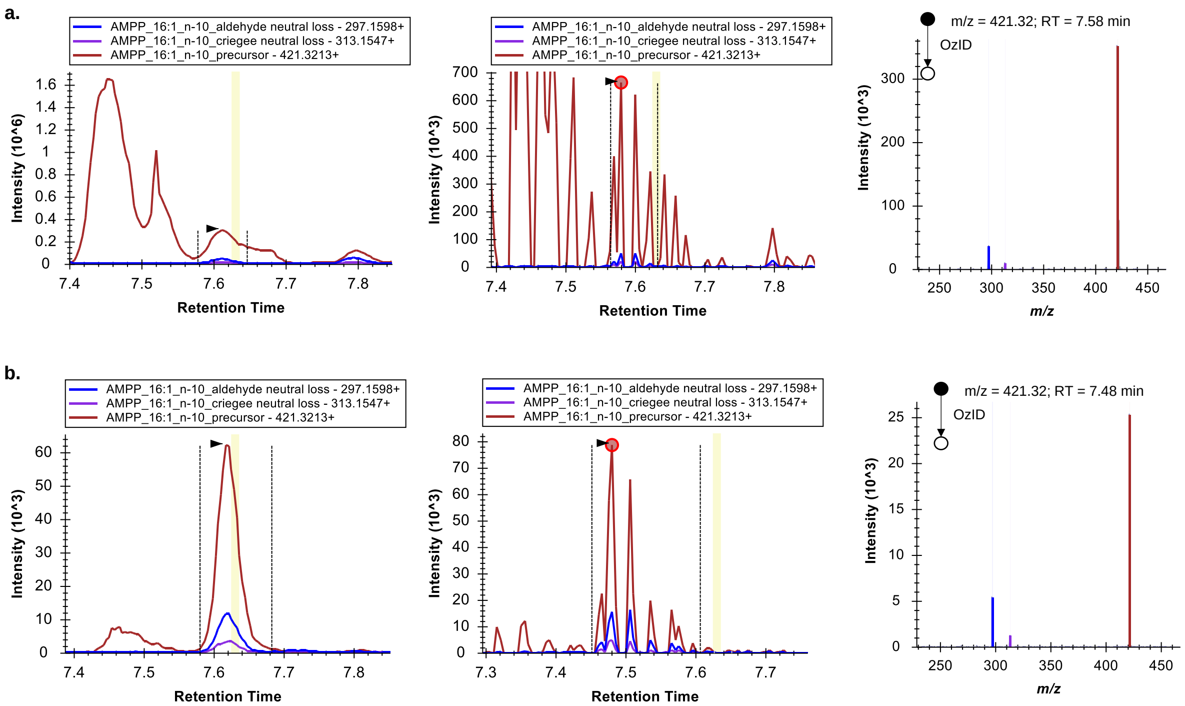

Supplementary Figure 21: Comparison of Sapienic acid in (**a.**) pooled human plasma (NIST 1950 Standard Reference Material) and (**b.**) the associated Process Blank. For each sample, the chromatograms of the data-independent acquisition are shown on the right, chromatograms of the data-dependent acquisition in the middle and an associated MS/MS spectrum on the right (the red dot in the middle indicates the associated retention time). The intensity scale clearly indicates that the amount of hexadecenoic acids in the human plasma sample is far higher than in the Process Blank, whereas the intensity of the internal standard is nearly equal (omitted for clarity).

3.3 Supplementary Note 3

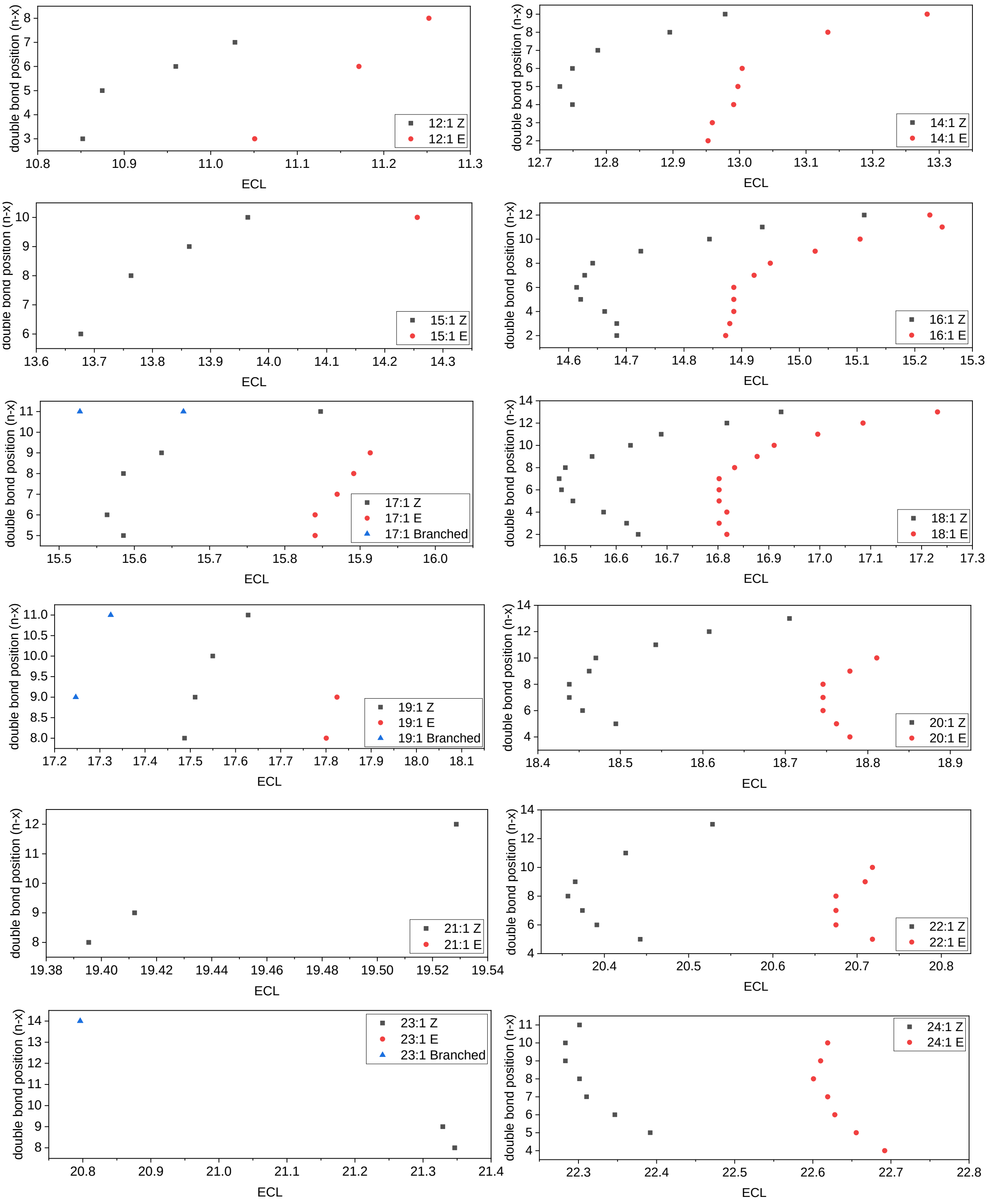

Supplementary Figure 22: Equivalent chain lengths (ECL) of monounsaturated fatty acids detected by data-dependent acquisition in NIST 1950 Standard Reference Material (Pooled Human Plasma). ECLs are calculated from observed retention times of the respective chromatographic peak of the OzID product chromatograms in the UPLC-OzID-MS/MS data. An expression was determined as a polynomial fit to the plot of the chain lengths of saturated fatty acids vs. their observed retention time in the same dataset as ECL = 0.0436 * $(RT)^2$ + 0.7393 * RT + 6.6932. For saturated fatty acids the equivalent chain length is defined as the number of carbon atoms in the fatty acyl chain, see also Supplementary Figure 22. The equivalent chain lengths of monounsaturated fatty acids are used here to assign E/Z conformation and to assign branched vs. straight chain fatty acids.

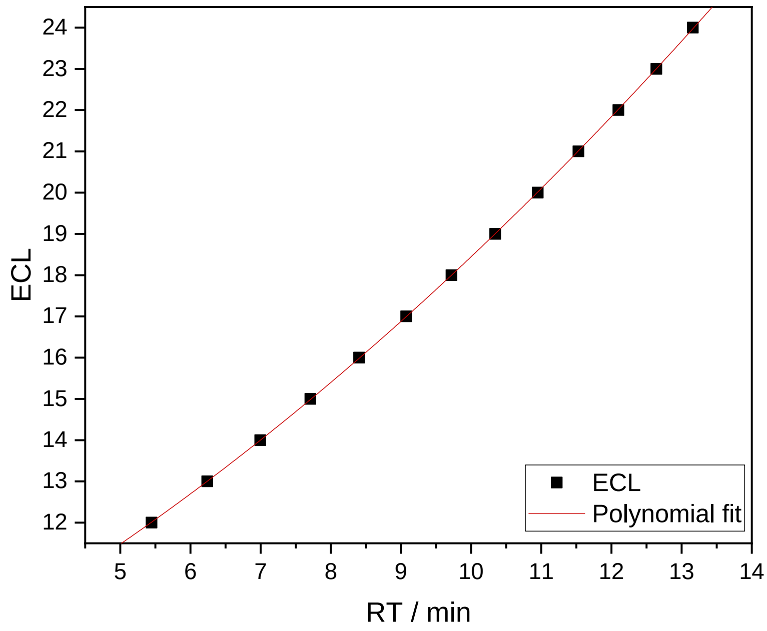

Supplementary Figure 23: Polynomial fit of equivalent chain lengths vs. retention times of saturated fatty acids (straight chain) in NIST 1950 Standard Reference Material (Pooled Human Plasma), technical replicate 1 (ECL = 0.0436 * (RT)^2 + 0.7393 * RT + 6.6932).

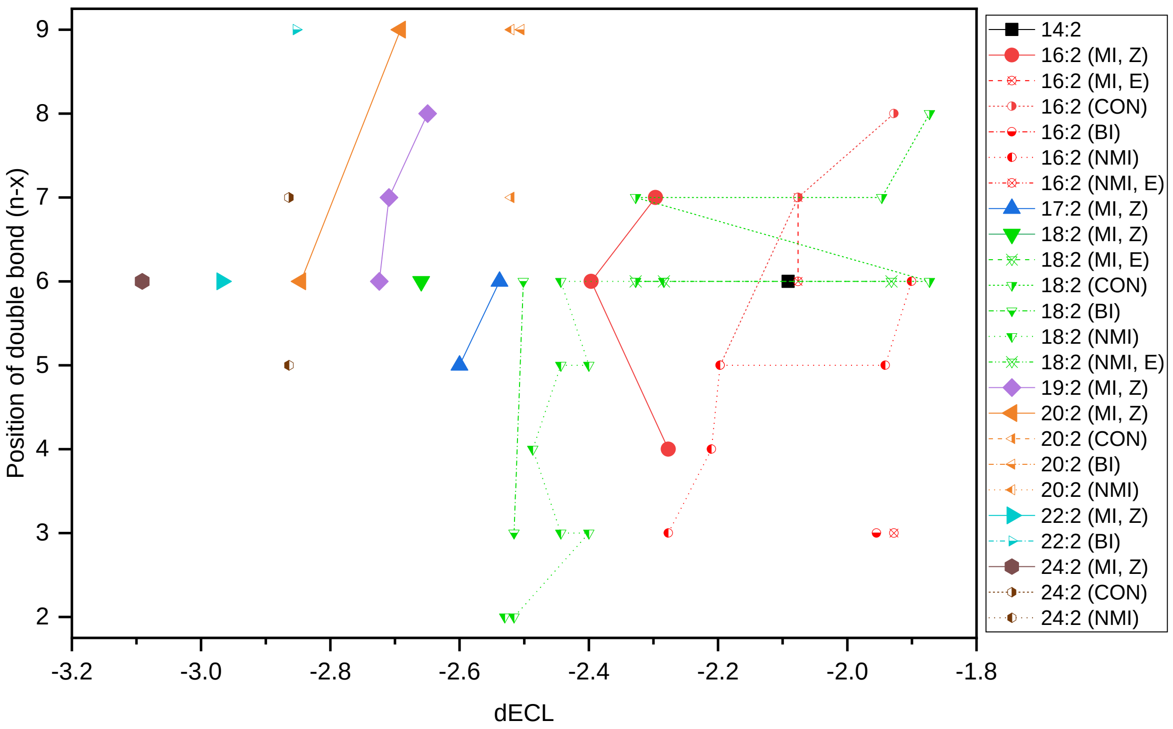

Supplementary Figure 24: Analysis of differential equivalent chain lengths (dECL) for bisunsaturated fatty acids in NIST 1950 SRM. Canonical methylene-interrupted species (MI) are highlighted with larger symbols. Non-methylene-interrupted fatty acids are assigned as conjugated (CON), butylene-interrupted (BI) or other non-methylene-interrupted fatty acids (NMI). Double bond configurations cannot be established unequivocally for all fatty acid species. Note that some identifications of FA 18:2 and FA 16:2 isomers are made only tentatively. For determination of ECL and dECL values, the same equation that was used for monounsaturated fatty acids was employed here (ECL = 0.0436 * $(RT)^2$ + 0.7393 * RT + 6.6932).

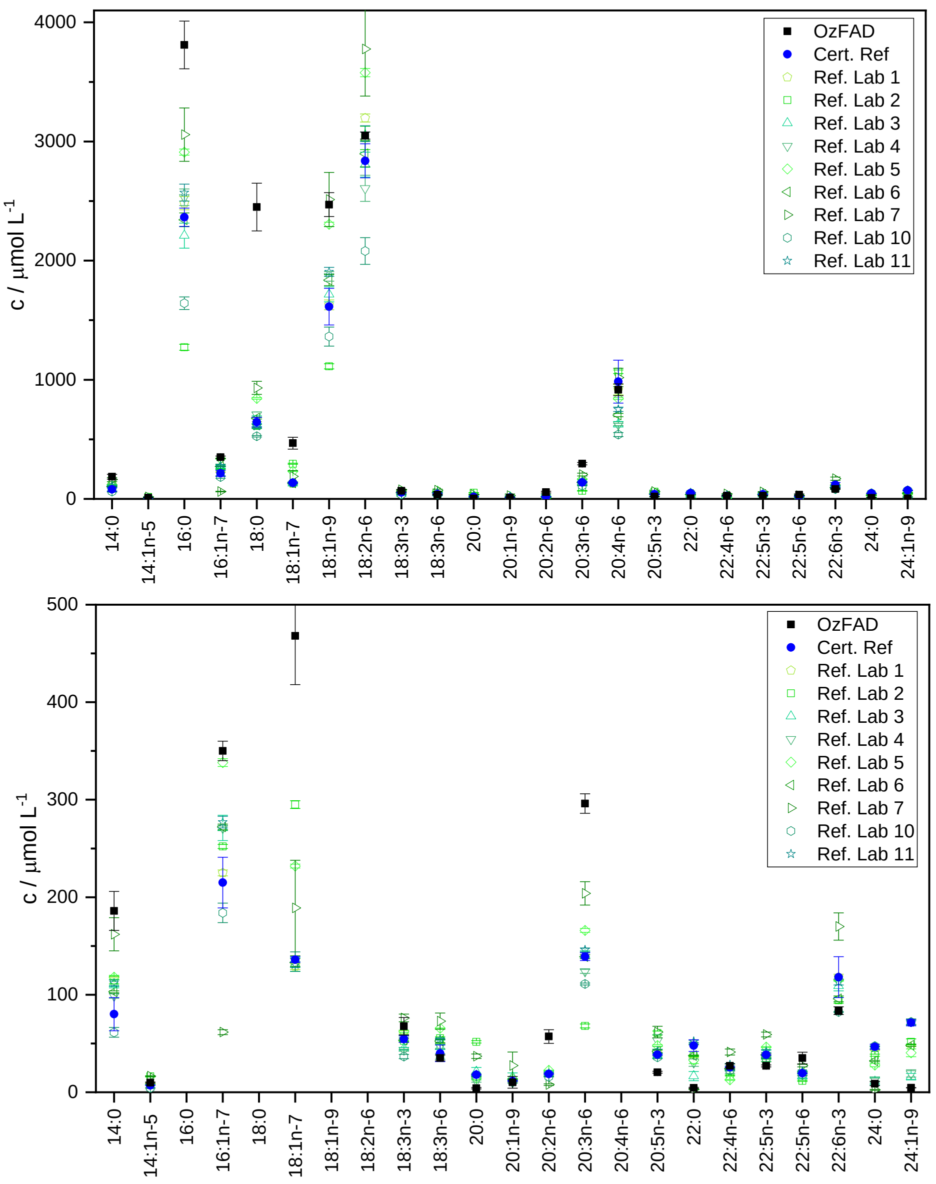

Supplementary Figure 25: Comparison of quantities of fatty acids (total fatty acid content) in the NIST 1950 SRM quantified by the OzFAD workflow (data shown are mean values of n=3 technical replicates and the error bars represent the standard deviation) and values reported by the U.S. Centre for Disease Control and Prevention (CDC) based on an international interlaboratory analytical comparison study (data are mean values of n=3 technical replicates and the error bars represent the standard deviation).[10] The certified reference value is shown as blue filled circles and individual data from the participating laboratories in the interlaboratory study are shown as green open symbols. The two graphs show the same data scaled differently to allow visualization of fatty acids at high abundance (top) and low abundance (bottom).

The mean average accuracy of the fatty acids shown in Supplementary Figure 24 (mean of the percentage of the absolute deviation of the obtained quantity from the certified reference value) is 78% (63% for the unsaturated fatty acids shown). The same calculation for the reference laboratories within the study (mean average of the mean of the percentage of the absolute deviation of the reported quantity from the certified reference value) is 27% (25% for the unsaturated fatty acids shown). This shows that the data obtained through the OzFAD workflow can – without requiring libraries or reference standards – provide best estimates of fatty acid quantities, with the limitation of less accurate quantities as the ones that may be provided by laboratories specializing on the quantification of fatty acids (using fatty acid standards to perform quantification). Highly accurate quantification is outside the scope of the current work, which is focused on the discovery of novel isomers and completing fatty acid profiles in complex samples.

Supplementary Table 4: Discoveries of *cis* unsaturated fatty acids in human plasma NIST 1950 SRM. The listed fatty acids were, to the best of our knowledge, not reported in the literature prior to the submission of this work in November 2022. Common names are assigned herein for each fatty acid. Complete data including estimates of quantities and tentative identifications of di-unsaturated fatty acids are included in Supplementary Data 1.

Fatty acid (n-x)	Systematic name	Common Name
FA 12:1n-6	6Z-dodecenoic acid	Brownic acid
FA 14:1n-6	8Z-tetradecenoic acid	Goldsteinic acid
FA 14:1n-4	10Z-tetradecenoic acid	Yageranic acid
FA 16:1n-4	12Z-hexadecenoic acid	Criegeenic acid
FA 16:1n-2	14Z-hexadecenoic acid	Ozoneic acid
FA 18:1n-2	16Z-octadecenoic acid	Ohsuminic acid
FA 19:1n-11	8Z-nonadecenoic acid	Benfieldic acid
FA 20:1n-8	12Z-eicosenoic acid	Turrbaleic acid
FA 21:1n-8	13Z-heneicosenoic acid	Henriqueic acid
FA 22:1n-6	16Z-docosenoic acid	Quandamookaic acid
FA 22:1n-8	14Z-docosenoic acid	Scottic acid
FA 23:1n-8	15Z-tricosenoic acid	Yugambehic acid
FA 24:1n-8	16Z-tetracosenoic acid	Butleric acid
FA 24:1n-11	13Z-tetracosenoic acid	Wakkawakkanic acid
FA 24:1n-6	18Z-tetracosenoic acid	Gubbigubbic acid
FA 19:2n-8,11	8Z,11Z-nonadecadienoic acid	Deisenhoferic acid
FA 20:2n-7,9	11,13-eicosadienoic acid	Hubereic acid
FA 24:2n-7,9	15,17-tetracosadienoic acid	Micheleic acid
FA 24:2n-5,9	15,19-tetracosadienoic acid	Burreic acid
FA 18:3n-3,7,9	9,11,15-octadecatrienoic acid	Kaurnaic acid
FA 18:3n-4,7,9	9,11,14-octadecatrienoic acid	Kgaric acid
FA 20:3n-7,10,12	8,10,13-eicosatrienoic acid	Blocheic acid
FA 22:4n-3,6,9,12	10Z,13Z,16Z,19Z-docosatetraenoic acid	Lynenic acid

Supplementary table 5: Discoveries of *trans* unsaturated fatty acids in human plasma NIST 1950 SRM. The listed fatty acids were, to the best of our knowledge, not reported in the literature prior to the submission of this work in November 2022. Complete data including estimates of quantities and tentative identifications of di-unsaturated fatty acids are included in Supplementary Data 1.

**Fatty acid (n-x)**	**Systematic name**
FA 12:1n-6_(E)	6E-dodecenoic acid
FA 14:1n-2_(E)	12E-tetradecenoic acid
FA 14:1n-4_(E)	10E-tetradecenoic acid
FA 14:1n-6_(E)	8E-tetradecenoic acid
FA 14:1n-8_(E)	6E-tetradecenoic acid
FA 14:1n-9_(E)	5E-tetradecenoic acid
FA 15:1n-10_(E)	5E-pentadecenoic acid
FA 16:1n-2_(E)	14E-hexadecenoic acid
FA 16:1n-3_(E)	13E-hexadecenoic acid
FA 16:1n-4_(E)	12E-hexadecenoic acid
FA 16:1n-11_(E)	5E-hexadecenoic acid
FA 16:1n-12_(E)	4E-hexadecenoic acid
FA 17:1n-5_(E)	12E-heptadecenoic acid
FA 17:1n-6_(E)	11E-heptadecenoic acid
FA 17:1n-8_(E)	9E-heptadecenoic acid
FA 17:1n-9_(E)	8E-heptadecenoic acid
FA 18:1n-11_(E)	7E-octadecenoic acid
FA 19:1n-8_(E)	11E-nonadecenoic acid
FA 20:1n-6_(E)	14E-eicosenoic acid
FA 20:1n-8_(E)	12E-eicosenoic acid
FA 20:1n-10_(E)	10E-eicosenoic acid
FA 20:1n-4_(E)	16E-eicosenoic acid
FA 20:1n-5_(E)	15E-eicosenoic acid
FA 20:1n-7_(E)	13E-eicosenoic acid
FA 22:1n-7_(E)	15E-docosenoic acid
FA 22:1n-8_(E)	14E-docosenoic acid
FA 22:1n-6_(E)	16E-docosenoic acid
FA 22:1n-10_(E)	12E-docosenoic acid
FA 22:1n-5_(E)	17E-docosenoic acid
FA 24:1n-10_(E)	14E-tetracosenoic acid
FA 24:1n-4_(E)	20E-tetracosenoic acid
FA 24:1n-8_(E)	16E-tetracosenoic acid
C18:3n-3,6,9_(E)	9,12,15-octadecatrienoic acid
FA 18:3n-6,9,12_(E)	6,9,12-octadecatrienoic acid
FA 18:3n-3,6,9_(E)	9,12,15-octadecatrienoic acid

3.3.2 Orthogonal validation by GC-CACI-MS/MS

For the purpose of validation with an orthogonal analytical method, we describe here the detection of sciadonic acid (FA 20:3*n*-6,9,15) by GC-CACI-MS/MS (gas chromatography solvent-mediated covalent adduct chemical ionization tandem mass spectrometry[17]) in the hydrolyzed lipid extract of pooled human plasma NIST 1950 SRM.

Following the extraction of lipids based on the Matyash protocol as described previously, 200 µL of the MTBE extract, derived from an equivalent of 40 µL human plasma NIST 1950 SRM, were subjected to evaporation and subsequently dissolved in 32 µL methanol and 16 µL chloroform. 30 µL of a 0.25 M solution of trimethylsulfonium hydroxide in methanol were added and the mixture was vortex-mixed for 2 minutes to yield fatty acid methyl esters (FAMEs) of the fatty acids and lipids in the extract.

A Shimadzu GCMS-8050 triple quadrupole mass spectrometer, equipped with a BPX-70 column (70% cyanopropyl polysilphenylene-siloxane, 25 m × 0.22 mm i.d. × 0.25 *µ*m film), was modified for solvent mediated (SM) covalent-adduct chemical ionization (CACI) mass spectrometry.[17,23] The temperature of the GC oven was initially held at 80 °C for 2 min. Subsequently, the temperature was increased at a rate of 15 °C min^{-1} to 170 °C (6 min), held for 4 min before being increased at a rate of 7 °C min^{-1} to 240 °C (10 min) and held for 3 min. The 26 min GC-MS run concludes with 1 min equilibration to bring the oven temperature to 80 °C before the next injection. The injector was operated in splitless injection mode at a temperature of 250 °C, while the ion source and interface were kept at 240 °C. The detector voltage was 1.48 kV and the scanning time 0.2 s. The carrier gas was helium, and the column flow was set to a linear velocity of 46.2 cm s^{-1}. The gas pressure was 123.5 kPa with a total flow of 17.4 mL min^{-1}, column flow of 1.31 mL min^{-1} and purge flow of 3.0 mL min^{-1}. For chemical ionization, acetonitrile was kept in the reservoir (reagent gas) to maintain a flow (acetonitrile vapour in argon gas) of 20 kPa using an automatic pressure control unit (APC). Collision energy for CACI MS/MS experiments (product ion scan) was optimized for sciadonic acid as 8 eV.

Comparison of LC-OzID-MS/MS and GC-CACI-MS/MS reveals that the latter requires optimization of collision energies for each fatty acid, while any (known or unknown) fatty acid can be immediately detected extremely reproducibly with universal parameters by LC-OzID-MS/MS. Further, to yield good spectra of sciadonic acid in human plasma (Supplementary Figure 26), a high concentration was required to be injected, estimated as 0.024 mM sciadonic acid in the injected solution. In contrast, good spectra of sciadonic acid (S/N_{OzID} = 1085) were obtained using an estimated concentration of 38 nM sciadonic acid in the injected solution, respectively (Fig. 2). Despite this estimation not being a quantitatively reliable sensitivity analysis, the results show an advantage in sensitivity of LC-OzID-MS/MS over GC-CACI-MS/MS of two to three orders of magnitude in this case. Reasons include derivatization as a fixed-charge and the highly chemically selective reaction of ozonolysis leading only to two diagnostic product ions per double bond, rather than a range of fragment ions arising from collision-induced dissociation.

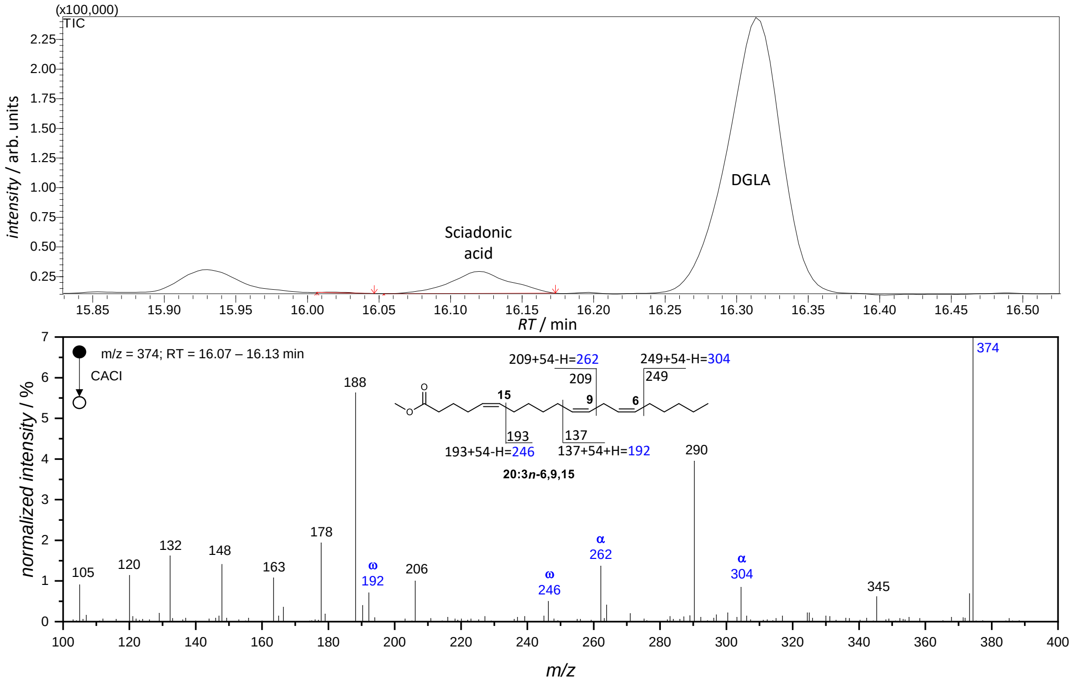

Supplementary Figure 26: Extracted ion chromatogram of FAME 20:3 of GC-CACI-MS/MS acquisition of methylated total fatty acids in pooled human plasma NIST 1950 SRM (top) and CACI-MS/MS spectrum of mass-selected FAME 20:3 (averaged MS/MS spectra over the range of 16.07 – 16.13 min). The diagnostic ions for sciadonic acid are highlighted in blue and are in excellent agreement with the previous detection of sciadonic acid in sea urchins *via* GC-CACI-MS/MS by Brenna and co-workers.[23]

3.4 Supplementary Note 4

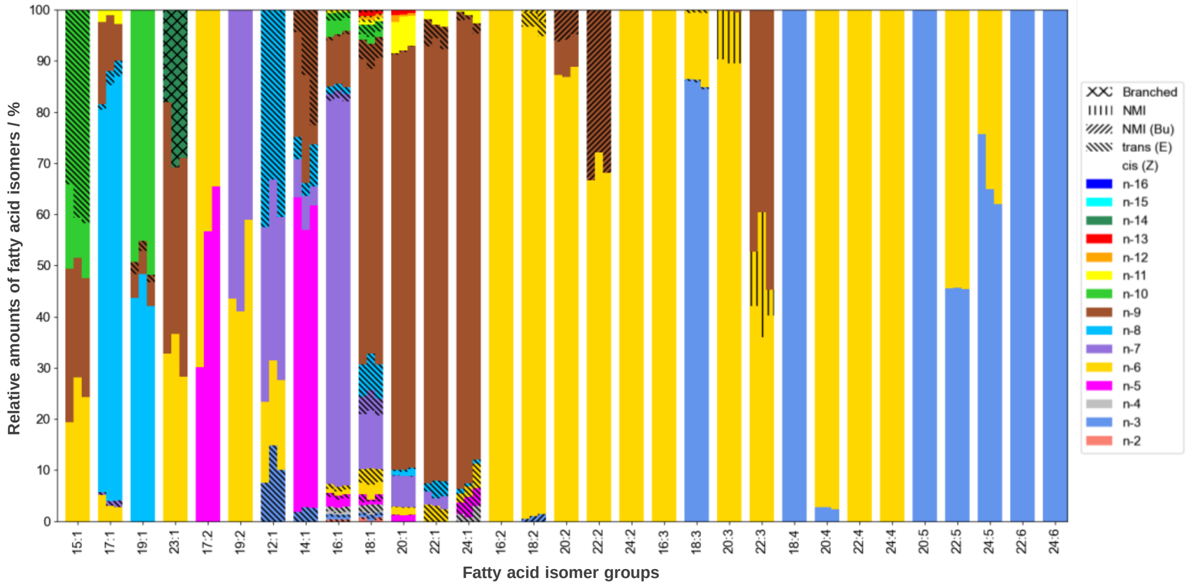

Supplementary Figure 27: Relative abundance of fatty acid isomers by isomer group in non-esterified fatty acids (NEFA) in NIST 1950 SRM.

Individual values and descriptors (analogous to Supplementary Table 4) for the fatty acids shown in Supplementary Figure 27 are shown in Supplementary Dataset 2.

3.5 Supplementary Note 5

Sample collection, lipid extraction, hydrolysis, and fixed charge derivatization (4-I-AMPP) were described previously. The derivatized fatty acids in Methanol were kept at -18°C prior to analysis with the methods described in this study. For each direct infusion ESI-MS measurement and LC-OzID-MS analyses (both untargeted and targeted analysis) 10 µL were injected.

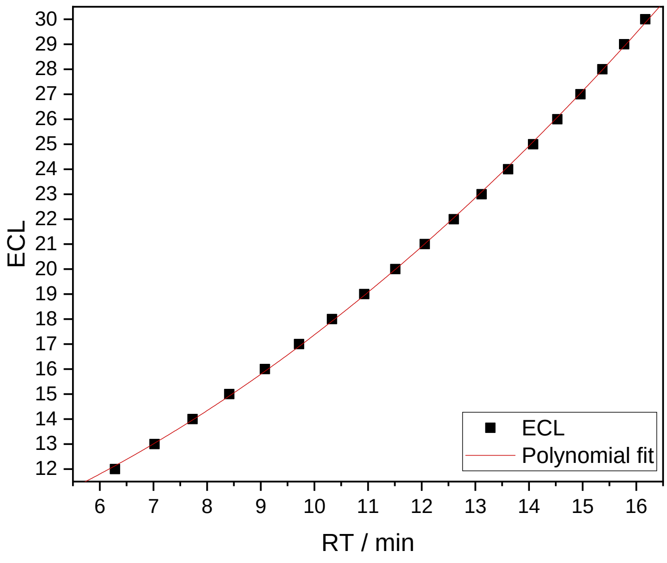

Supplementary Figure 28: Polynomial fit of equivalent chain lengths vs. retention times of saturated fatty acids (straight chain) in Vernix caseosa (ECL = 0.0621 * (RT)^2 + 0.39907 * RT + 7.1711).

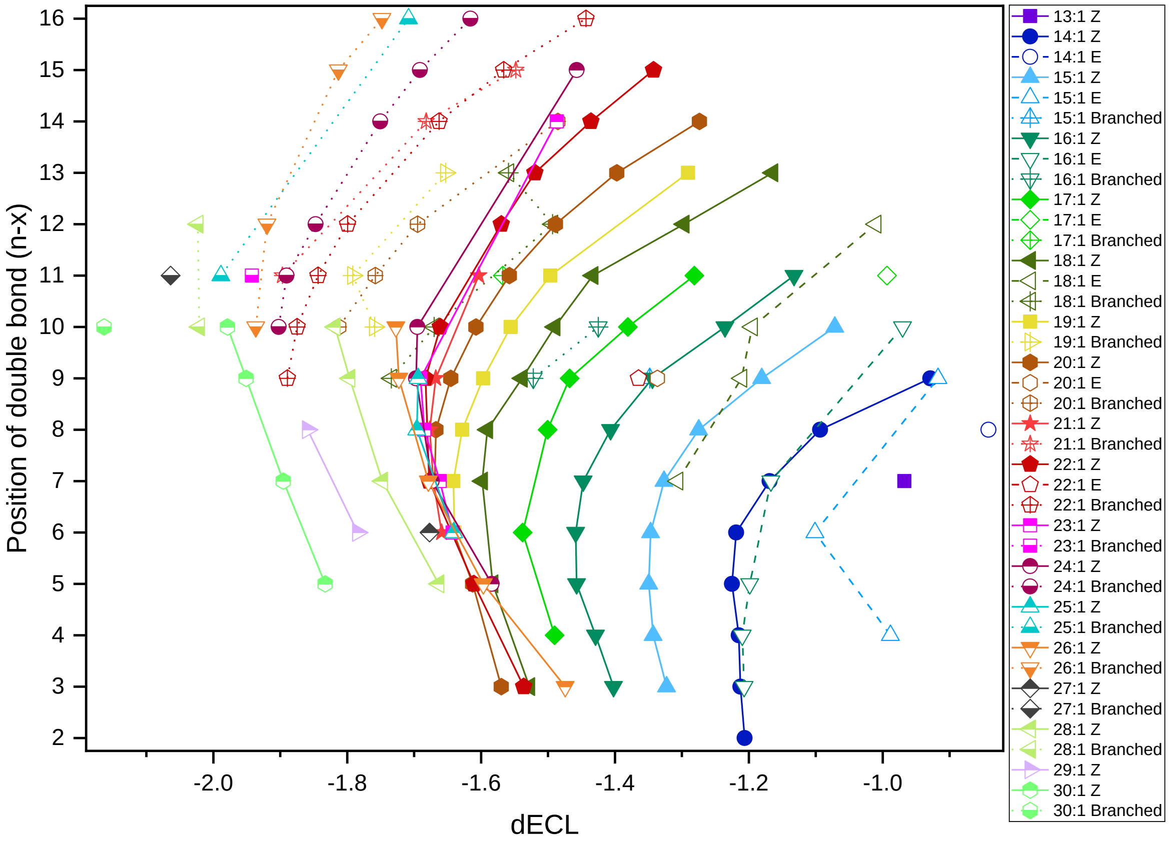

Supplementary Figure 29: Double bond positions of 4-I-AMPP derivatized fatty acid isomers found in vernix caseosa plotted against their dECL values as determined with the polynomial fit shown in Supplementary Figure 26.

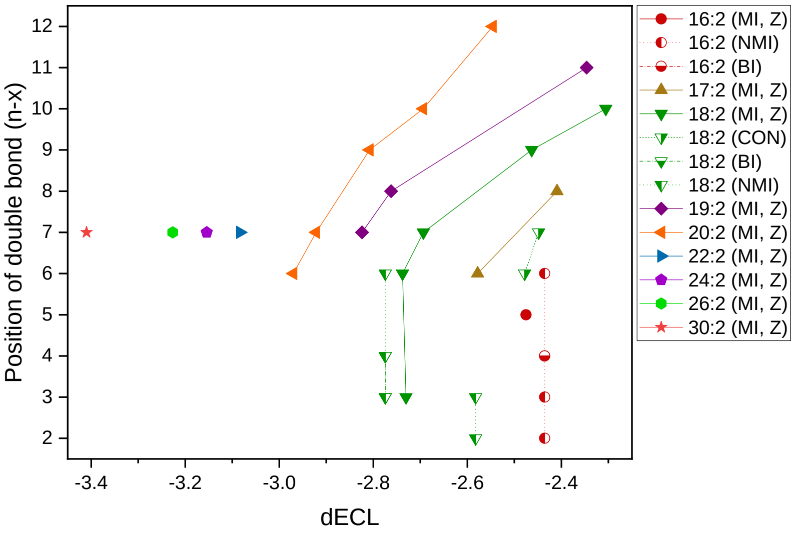

Supplementary Figure 30: Analysis of differential equivalent chain lengths (dECL) for bisunsaturated fatty acids in vernix caseosa extracts. Non-methylene-interrupted fatty acids are assigned as conjugated (CON), butylene-interrupted (BI) or other non-methylene-interrupted fatty acids (NMI). Double bond configuration cannot be established unequivocally for all fatty acid species, but the analysis allows a tentative assignment as *cis* for the majority of fatty acids that are observed. For determination of ECL and dECL values the equation (ECL = 0.0621 * $(RT)^2$ + 0.39907 * RT + 7.1711) was determined from retention times of saturated fatty acids in the same sample.

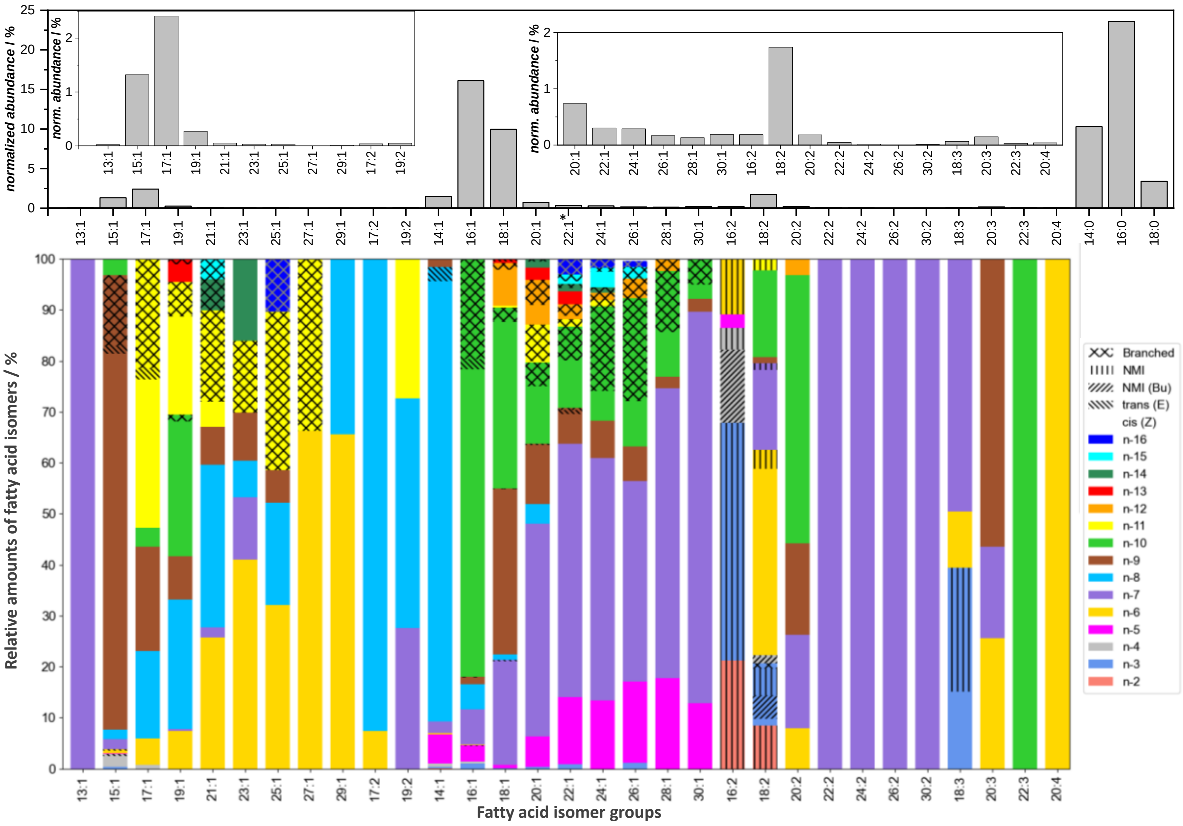

Supplementary Figure 31: Unsaturated fatty acids and their relative abundance in vernix caseosa. A lipid extract of vernix caseosa was hydrolyzed and derivatized with 4-I-AMPP prior to UPLC-OzID-MS analysis. Positions of branch points cannot be determined by Ozone-induced dissociation and are not reported here. Above: Relative quantification of fatty acid isomer groups without discrimination of isomers. Below: Relative quantification of fatty acids for each isomer group based on LC-OzID-MS and LC-OzID-MS/MS. *The plastics additive erucamide and erucic acid derived from it are detected both in the vernix caseosa extract as well as the associated Process Blank. The data shown here is subtracted with the amounts detected in the Process Blank. Data as shown here are shown in Supplementary Data 3.

Supplementary table 6: Discoveries of unsaturated fatty acids in vernix caseosa. Fatty acids that have been identified in vernix caseosa, but also in human plasma, see Supplementary table 4 and 5, are excluded here. The listed fatty acids were, to the best of our knowledge, not reported in the literature prior to the submission of this work in November 2022. Common names are assigned herein. Complete data including estimates of quantities are included in Supplementary Data 3.

Fatty acid (n-x)	Systematic name	Common Name
FA 13:1n-7	6Z-tridecenoic acid	Chevreulic acid
FA 14:1n-2	12Z-tetradecenoic acid	Seylereic acid
FA 15:1n-3	12Z-pentadecenoic acid	Fertilitatic acid
FA 15:1n-4	11Z-pentadecenoic acid	Liebigeic acid
FA 15:1n-7	8Z-pentadecenoic acid	Berthelotic acid
FA 15:1n-4_(E)	11E-pentadecenoic acid	
FA 15:1n-6_(E)	9E-pentadecenoic acid	
FA 15:1n-9_(E)	6E-pentadecenoic acid	
FA 17:1n-4	13Z-heptadecenoic acid	Thudichumic acid
FA 17:1n-11_(E)	6E-heptadecenoic acid	
FA 20:1n-3	17Z-eicosenoic acid	Freiburgeic acid
FA 21:1n-6	15Z-heneicosenoic acid	Verkadeic acid
FA 21:1n-7	14Z-heneicosenoic acid	Dyerbergeic acid
FA 21:1n-11	10Z-heneicosenoic acid	Vrkoslavic acid
FA 22:1n-10	12Z-docosenoic acid	Bangeic acid
FA 22:1n-14	8Z-docosenoic acid	Fredricksonic acid
FA 22:1n-12	10Z-docosenoic acid	Superficienic acid
FA 24:1n-15	9Z-tetracosenoic acid	Cutineic acid
FA 26:1n-10	16Z-hexacosenoic acid	Neonatic acid
FA 26:1n-3	23Z-hexacosenoic acid	Matereic acid
FA 27:1n-6	21Z-heptacosenoic acid	Cudlmanic acid
FA 28:1n-10	18Z-octacosenoic acid	Cvackaic acid
FA 29:1n-6	23Z-nonacosenoic acid	Urbanic acid
FA 29:1n-8	21Z-nonacosenoic acid	Vojiroic acid
FA 30:1n-10	20Z-triacontenoic acid	Patroneic acid
FA 30:1n-5	25Z-triacontenoic acid	Lavoisieric acid
FA 16:2n-2,10	6,14-hexadecadienoic acid	Scutanic acid
FA 16:2n-3,10	6,13-hexadecadienoic acid	Menzeleic acid
FA 17:2n-8,11	6Z,9Z-heptadecadienoic acid	Nascentianic acid
FA 18:2n-3,10	8,15-octadecadienoic acid	Foigeic acid
FA 19:2n-11,14	5Z,8Z-nonadecadienoic acid	Orbonaic acid
FA 20:2n-7,10	10Z,13Z-eicosadienoic acid	Verniceic acid
FA 20:2n-10,13	7Z,10Z-eicosadienoic acid	Dermateic acid
FA 20:2n-12,15	5Z,8Z-eicosadienoic acid	Meanjinic acid
FA 22:2n-7,10	12Z,15Z-docosadienoic acid	Feteic acid
FA 24:2n-7,10	14Z,17Z-tetracosadienoic acid	Prenateic acid
FA 26:2n-7,10	16Z,19Z-hexacosadienoic acid	Nativitatic acid
FA 30:2n-7,10	20Z,23Z-triacontadienoic acid	Progigneic acid
FA 18:3n-3,10,13	5,8,15-octadecatrienoic acid	Dinheic acid
FA 22:3n-10,13,16	6Z,9Z,12Z-docosatrienoic acid	Buekeic acid

3.5.2 Orthogonal validation by LC-UVPD-MS/MS

Selected discoveries (16:2*n*-3,10; 18:2*n*-3,10 and 18:3*n*-3,10,13) made in vernix caseosa *via* LC-OzID-MS/MS were additionally validated by the orthogonal method LC-UVPD-MS/MS (liquid chromatography ultraviolet photodissociation tandem mass spectrometry). These experiments were carried out on an Orbitrap Fusion Lumos, equipped with a 213 nm laser (CryLaS, max. radiant energy 3.0 μJ/pulse at 2.5 kHz). As described previously, lipids were extracted based on the Matyash protocol, lipids were hydrolyzed and derivatized with 4-I-AMPP.

Chromatographic separation was performed using an Acclaim™ C30 reversed phase column (2.1 x 150 mm, 3.0 μm particle size, Thermo). The 4-I-AMPP derivatized sample was diluted in methanol and 10 μL were injected for each acquisition. A gradient elution over 180 min at a flow rate of 0.3 mL / min at 45 °C was carried out using 50% : 30% : 20% water/acetonitrile/2-propanol with 10 mM ammonium formate as mobile phase A and 1% : 9% : 90% water/acetonitrile/2-propanol with 10 mM ammonium formate as mobile phase B. Initially, mobile phase B is linearly increased from 0% to 63% for 160 minutes, followed by an increase to 100% B, held until 170 min with subsequent reduction of B to 0% for the last 10 minutes of the chromatographic run.

Electrospray ionization is performed in positive mode with a spray voltage of 4 kV and dimensionless sheath gas parameters set to 10, auxiliary gas set to 5 and sweep gas set to 1. The ion transfer tube temperature is kept at 260°C and the vaporizer temperature at 250°C. A capillary voltage of 11 V is applied and the RF lens is set to 50%. Mass spectra were obtained at a resolution of R = 120000. An intensity threshold of 20000 is applied.

For MS^2 acquisition, an isolation width of 1.7 *m/z* is set on the quadrupole for mass selection. The activation time (photodissociation PD_{213}) is 150 ms. Mass spectra are acquired with a resolution of R = 15000.

MS^2 acquisition was performed in a scheduled analysis, allowing mass-selection of the 4-I-AMPP derivatized precursor fatty acid of interest before laser irradiation induces photodissociation. Initially, the carbon-iodine bond is cleaved, with subsequent fragmentation occurring along the fatty acyl chain. Fragment *m/z* values are indicative of the position of double bonds. Example UVPD-MS/MS spectra of FA 18:3*n*-3,6,9 present in both vernix caseosa and in a commercial standard (37 mix) are shown alongside the respective extracted ion chromatograms for the 4-I-AMPP derivatized FA 18:3 isomers (Supplementary Figure 32).

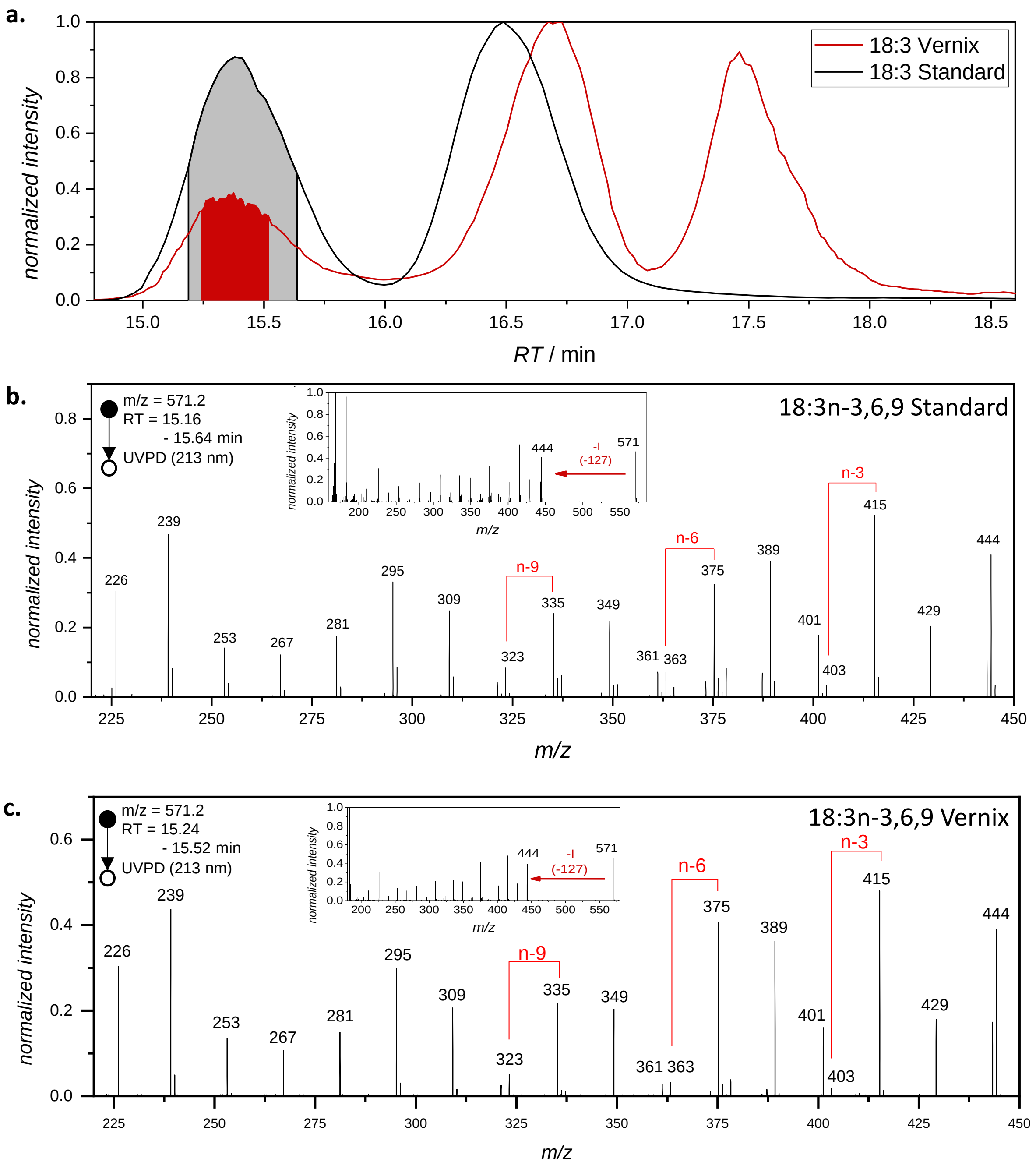

Supplementary Figure 32: a. Extracted ion chromatograms for FA 18:3 from the 37 mix standard sample and from vernix caseosa. Both samples underwent derivatization with 4-I-AMPP prior to acquisition via LC-UVPD-MS/MS. The 37 mix standard mixture contains both FA 18:3*n*-3,6,9 and FA 18:3*n*-6,9,12. Vernix caseosa contains FA 18:3*n*-3,6,9 and the newly discovered FA 18:3*n*-3,10,13 as well as FA 18:3*n*-7,10,13. The filled areas under the curve indicate the retention time range over which the UVPD-MS/MS spectra were averaged (shown below). **b.** UVPD-MS/MS spectrum of FA 18:3*n*-3,6,9 from the 37 mix standard. **c.** UVPD-MS/MS spectrum of FA 18:3*n*-3,6,9 from vernix caseosa. The UVPD-MS/MS spectra are highly similar and thus, enable assignment of the double bond positions of this polyunsaturated fatty acid.

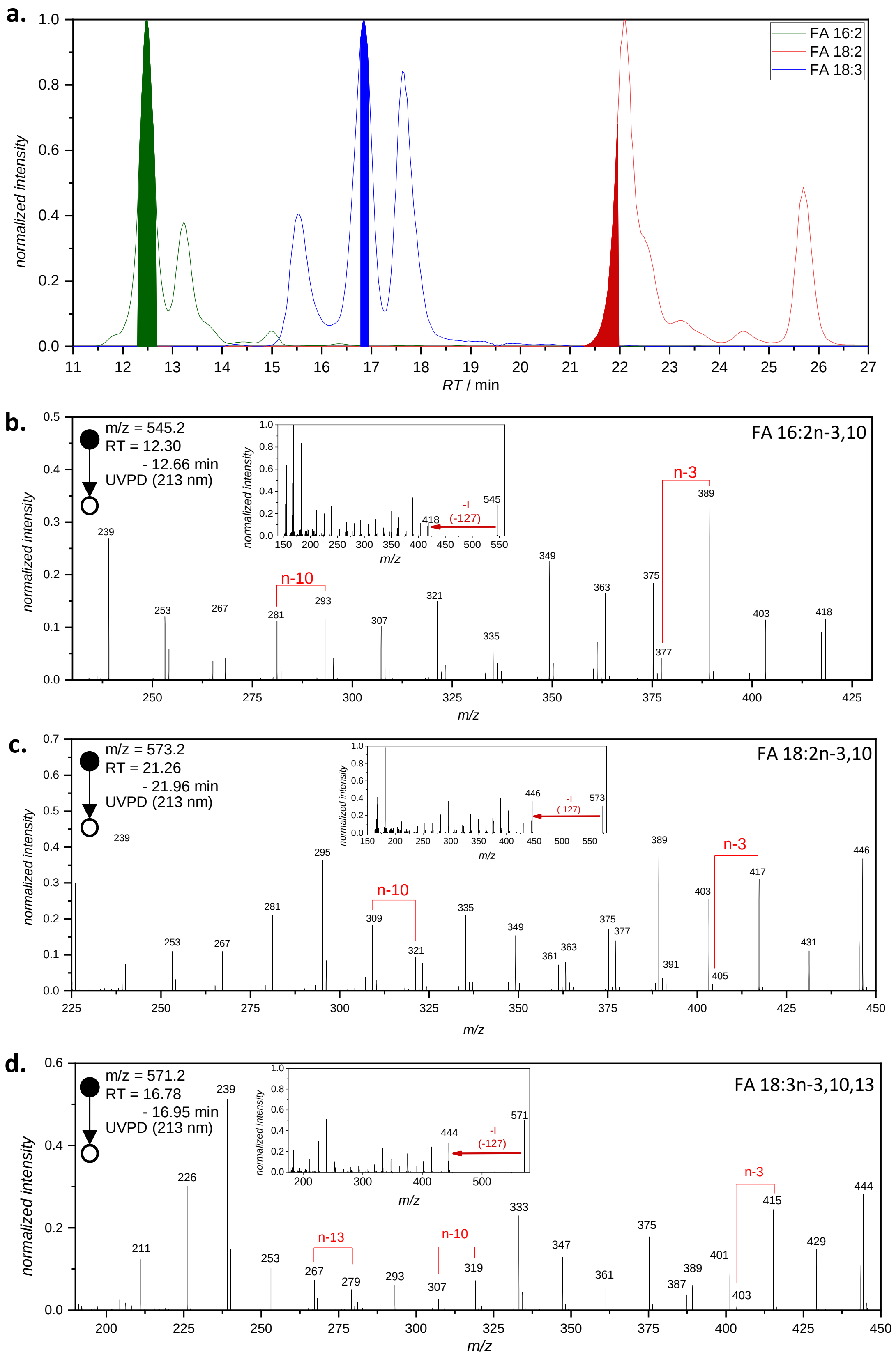

Supplementary Figure 33: Orthogonal validation of selected fatty acids that were discovered by the OzFAD workflow. **a.** Extracted ion chromatograms of unfragmented derivatized fatty acids FA 16:2, FA 18:2 and FA 18:3. The filled areas under the curve indicate the retention time ranges, over which the UVPD-MS/MS spectra were averaged, respectively, to yield the spectra displayed below. **b.** UVPD-MS/MS spectrum of FA 16:2*n*-3,10 (Menzeleic acid). **c.** UVPD-MS/MS spectrum of FA 18:2*n*-3,10 (Foigeic acid). **d.** UVPD-MS/MS spectrum of FA 18:2*n*-3,10,13 (Dinheic acid).

3.6 Supplementary Note 6

Fetal bovine serum analyzed here was heat inactivated prior to lipid extraction. Lipids from 0.1 mL fetal bovine serum were extracted and hydrolyzed as per the procedures described in the methods section of this publication. Fatty acids were derivatized with AMPP and analyzed according to the methods described within this work. Twelve fatty acid species were discovered in fetal bovine serum that have to the best of our knowledge not been reported previously (including discoveries reported in human plasma or vernix caseosa herein). Additionally, 42 fatty acids that were discovered for the first time in either human plasma or vernix caseosa within this work, were also identified in fetal bovine serum.

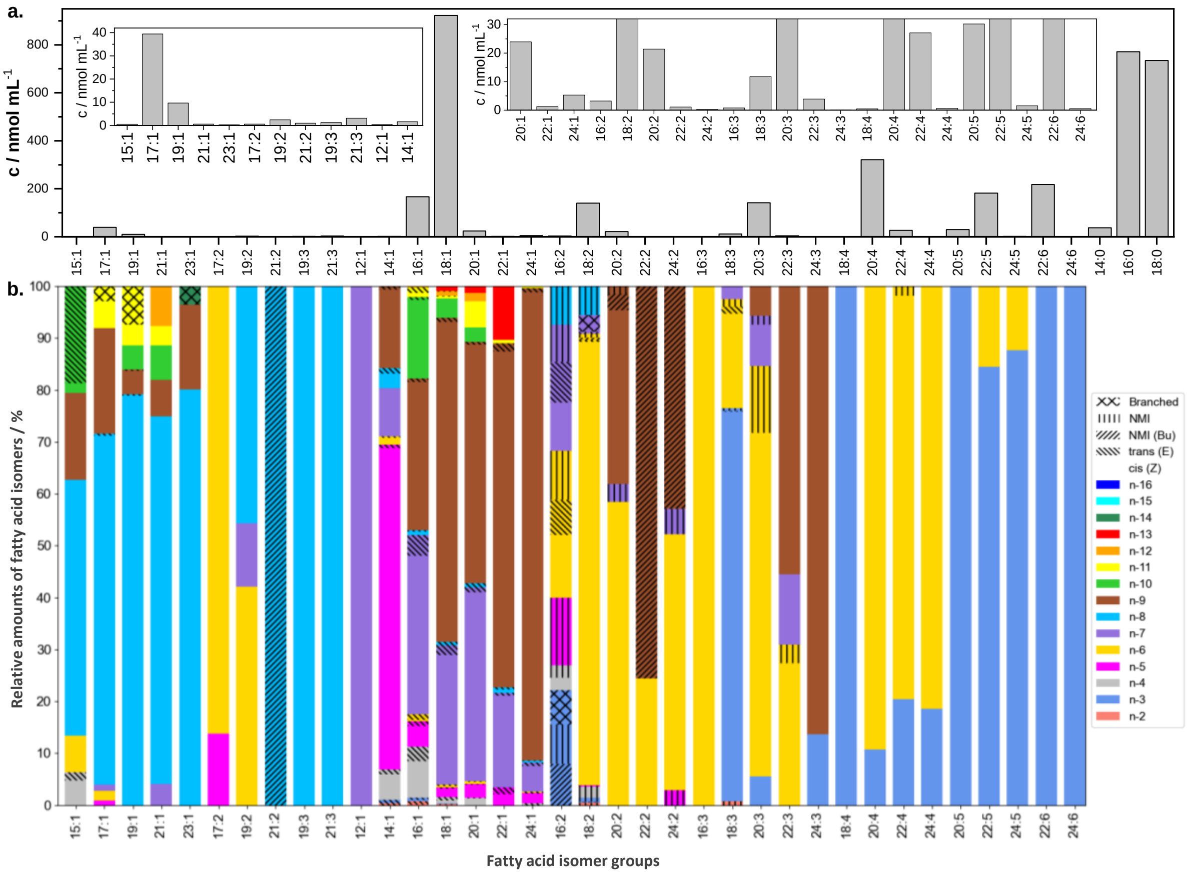

Supplementary Figure 34: Fatty acids in fetal bovine serum. **a.** Estimation of absolute quantities of non-isomeric fatty acids by direct infusion mass spectrometry. **b.** Relative amounts of fatty acid isomers by LC-OzID-MS and LC-OzID-MS/MS. Data associated to this Supplementary Figure are shown in Supplementary Data 4.

Supplementary table 7: Discoveries of unsaturated fatty acids in fetal bovine serum. Fatty acids that have been identified in fetal bovine serum, but also in vernix caseosa or human plasma, see Supplementary table 4, 5 and 6, are excluded here. The listed fatty acids were, to the best of our knowledge, not reported in the literature prior to the submission of this work in November 2022. Common names are assigned herein. Complete data including estimates of quantities are included in Supplementary Data 4.

Fatty acid (n-x)	Systematic name	Common Name
FA 21:1n-10	11Z-heneicosenoic acid	Capecchic acid
FA 21:2n-8,14	7,13-heneicosadienoic acid	Evanseic acid
FA 24:2n-9,15	9,15-tetracosadienoic acid	Smithiesic acid
FA 19:3n-8,11,14	5Z,8Z,11Z-nonadecatrienoic acid	Coreynic acid
FA 20:3n-6,8,12	8,12,14-eicosatrienoic acid	Gurdonic acid
FA 21:3n-8,11,14	7Z,10Z,13Z-heneicosatrienoic acid	Yamanakanic acid
FA 22:3n-7,10,13	9Z,12Z,15Z-docosatrienoic acid	Vaneic acid
FA 24:3n-9,12,15	9Z,12Z,15Z-tetracosatrienoic acid	Samuelssonic acid
FA 24:4n-3,6,9,12	12Z,15Z,18Z,21Z-tetracosatetraenoic acid	Bergstroemic acid

3.7 Supplementary Note 7

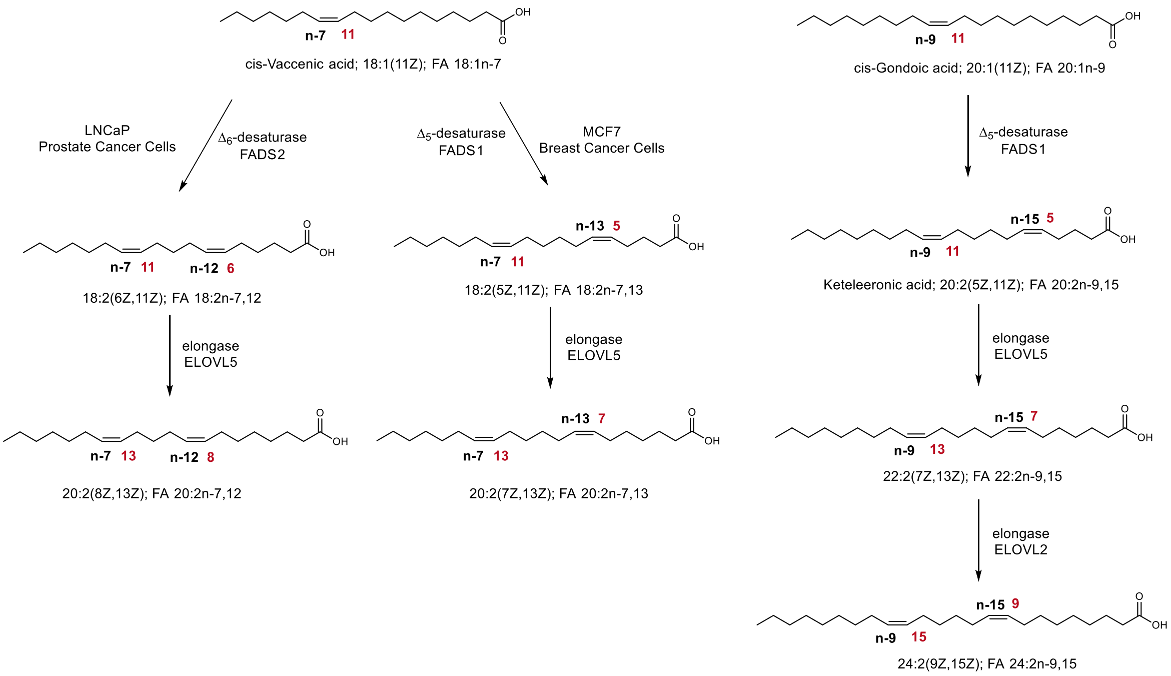

Supplementary Figure 35: Similar to a proposed biosynthetic pathway to sebaleic acid by Ferreri et al.[46] we propose pathways to (non-methylene-interrupted) fatty acids in cancer cell lines.

3.7.1 MCF7 Breast Cancer - Human breast adenocarcinoma, metastatic

The MCF7 cell line, named after the Michigan Cancer Foundation, consists of epithelial cells from a metastatic adenocarcinoma (breast tissue) of a 69-year-old female patient of Caucasian ethnic background.

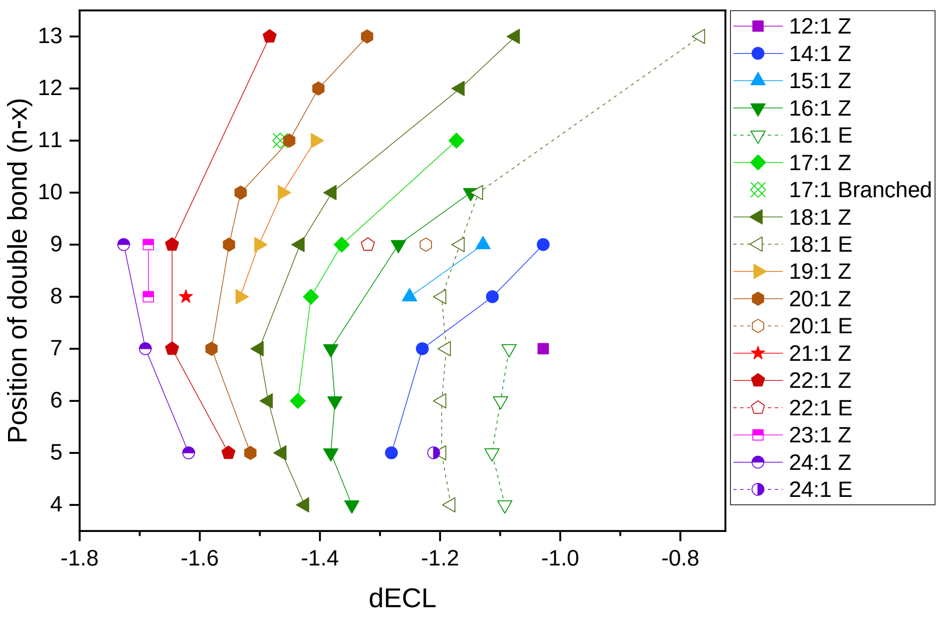

Supplementary Figure 36: Analysis of differential equivalent chain lengths (dECL) of monounsaturated fatty acids in MCF7 cells. ECL and dECL values were determined with the equation ECL= 0.04115 * $(RT)^2$ + 0.79914 * RT + 6.50734, based on the retention times of saturated fatty acids in the same sample.

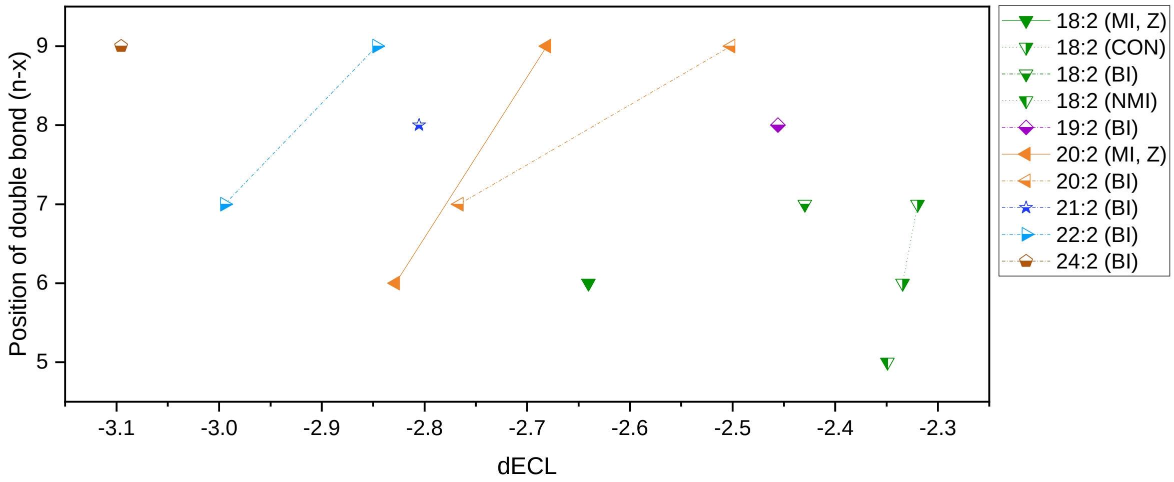

Supplementary Figure 37: Analysis of differential equivalent chain lengths (dECL) of bisunsaturated fatty acids in MCF7 cells. Non-methylene-interrupted fatty acids are assigned as conjugated (CON), butylene-interrupted (BI) or other non-methylene-interrupted fatty acids (NMI). Double bond configurations cannot be established unequivocally for all fatty acid species. ECL and dECL values were determined with the equation ECL= 0.04115 * $(RT)^2$ + 0.79914 * RT + 6.50734, based on the retention times of saturated fatty acids in the same sample.

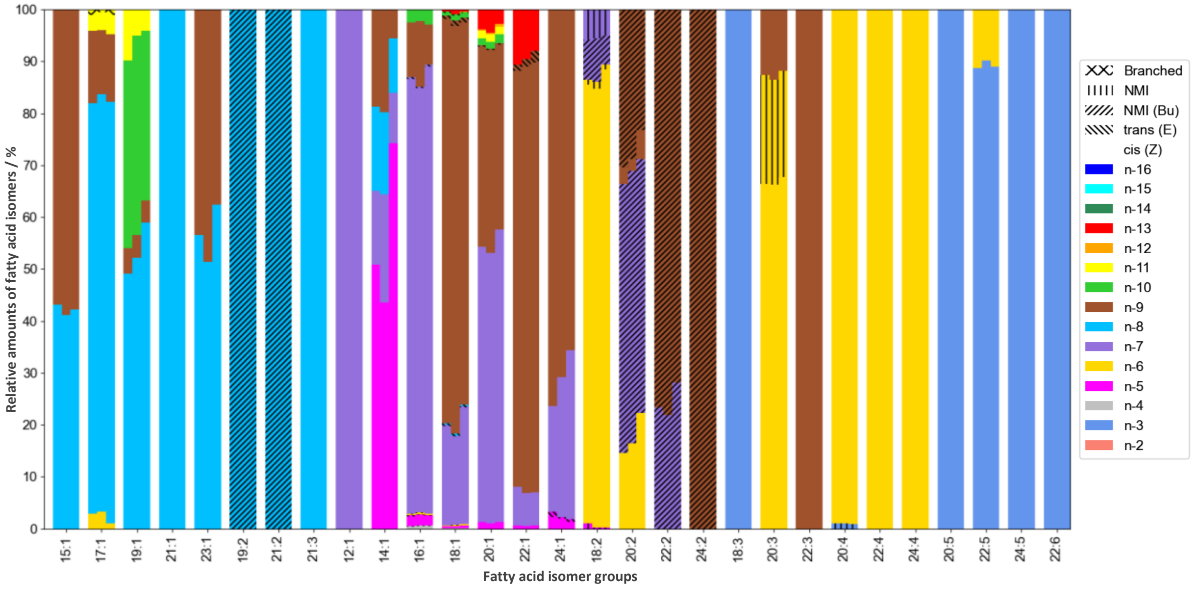

Supplementary Figure 38: Relative amounts of fatty acid isomers identified in lipid extracts of MCF7 cancer cells (total fatty acid content of hydrolyzed lipids). Shown are the relative amounts of three replicates (three instances of MCF7 cells undergoing cell culture). Note that the deviation between the replicates is the largest for tetradecenoic acids. For example, the third replicate shows a significantly larger relative amount of 9Z-tetradecenoic acid (n-5) compared to the other replicates. Yet, relative amounts of other fatty acid isomers are not deviating substantially between the replicates. Detailed data are shown in Supplementary Data 5.

3.7.2 LNCaP Lymph node carcinoma of the Prostate - Human Prostatic Cancer Cells

LNCaP cells are derived from the left supraclavicular lymph node (metastasis of prostate cancer) of a 50-year-old male patient.

Cell culture, lipid extraction, hydrolysis and fixed charge derivatization (AMPP) was described previously.[47] The derivatized fatty acids in Methanol were kept at -18°C prior to analysis with the methods described in this study.

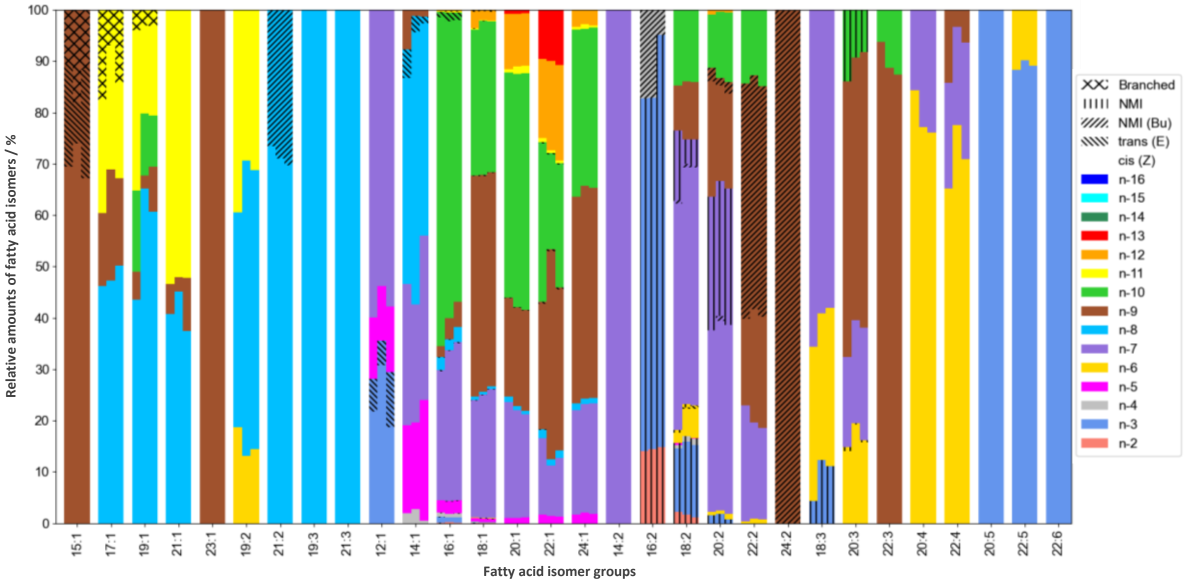

Supplementary Figure 39: Relative amounts of fatty acid isomers identified in lipid extracts of LNCaP cancer cells (total fatty acid content of hydrolyzed lipids). Shown are the relative amounts of three replicates (three instances of LNCaP cells undergoing cell culture).

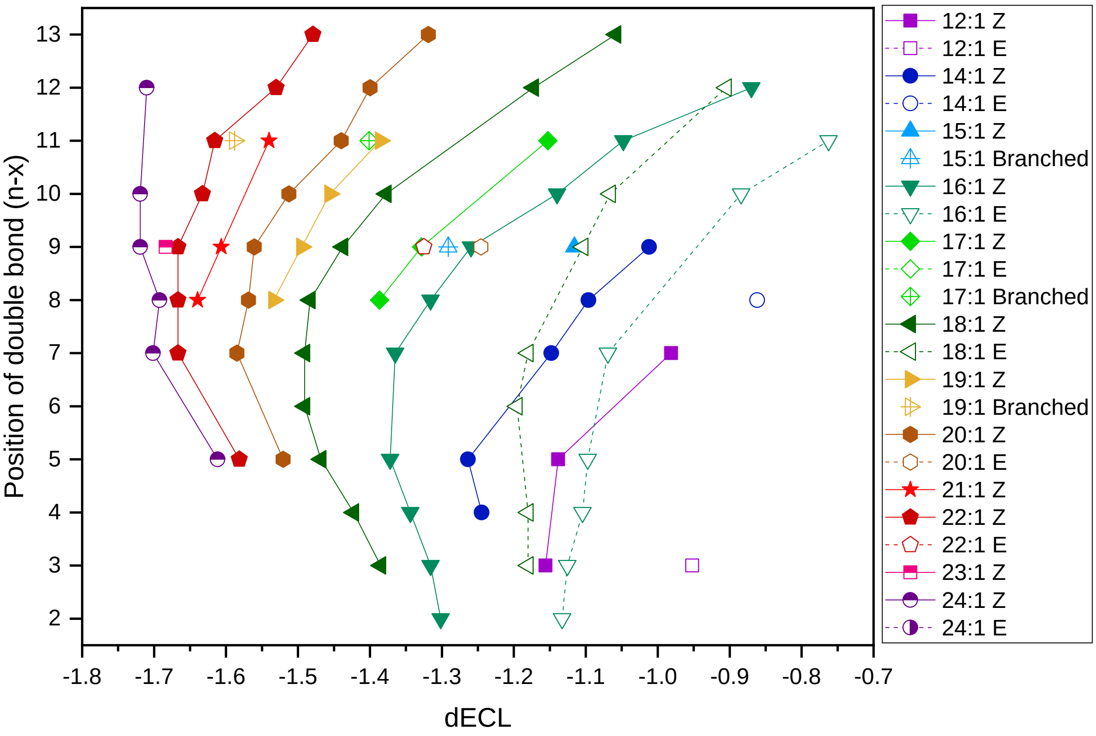

Supplementary Figure 40: Analysis of differential equivalent chain lengths (dECL) for monounsaturated fatty acids in LNCaP cell extracts. For determination of ECL and dECL values, the equation (ECL = 0.04095 * (RT)^2 + 0.79599 * RT + 6.53741) was determined from retention times of saturated fatty acids in the same sample.

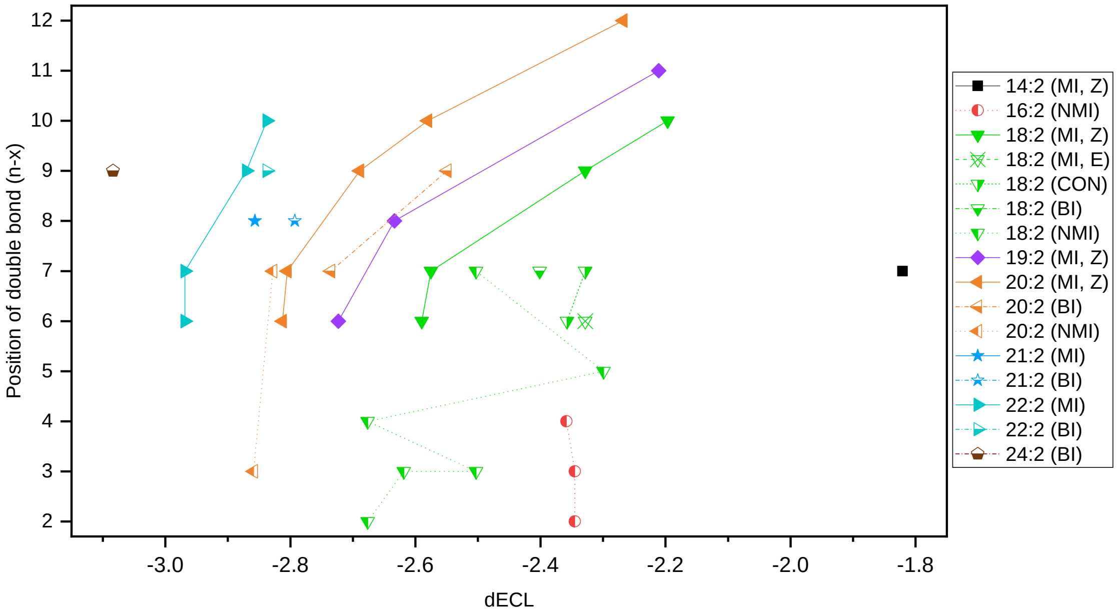

Supplementary Figure 41: Analysis of differential equivalent chain lengths (dECL) for bisunsaturated fatty acids in LNCaP cell extracts. Non-methylene-interrupted fatty acids are assigned as conjugated (CON), butylene-interrupted (BI) or other non-methylene-interrupted fatty acids (NMI). Double bond configurations cannot be established unequivocally for all fatty acid species. For determination of ECL and dECL values, the equation (ECL = 0.04095 * $(RT)^2$ + 0.79599 * RT + 6.53741) was determined from retention times of saturated fatty acids in the same sample.

3.7.3 LNCaP_SCD-1i Cancer of the Prostate - Human Prostatic Cancer Cells, SCD-1 inhibited

Cell culture, lipid extraction, hydrolysis and fixed charge derivatization (AMPP) was described previously.[47] The derivatized fatty acids in Methanol were kept at -18°C prior to analysis with the methods described in this study. Detailed results are provided in Supplementary Data 7.

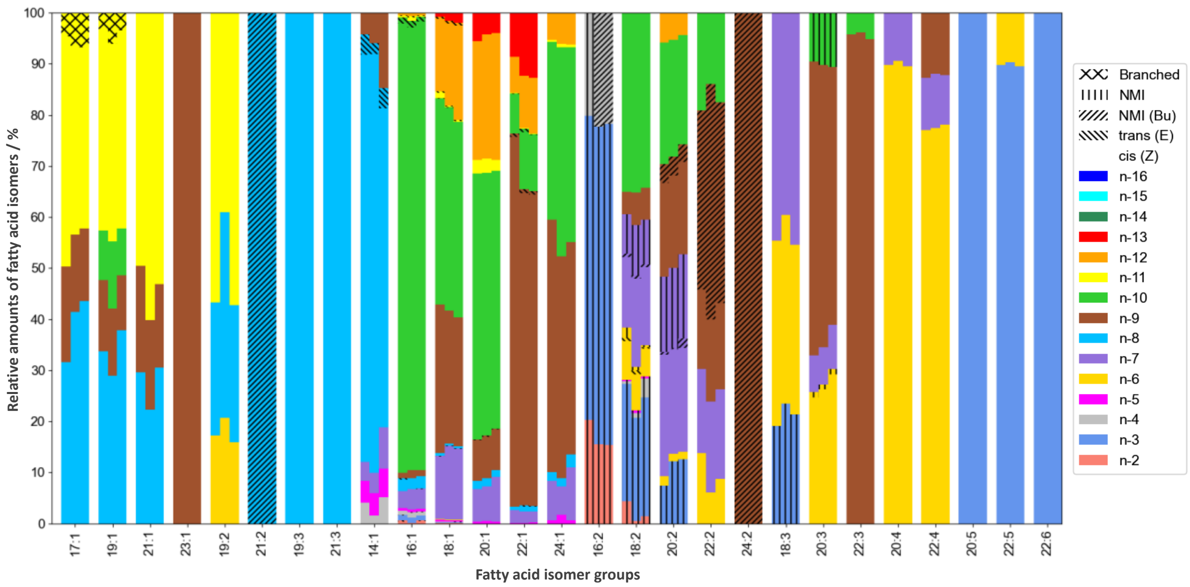

Supplementary Figure 42: Relative amounts of fatty acid isomers identified in lipid extracts of LNCaP_SCD-1i cancer cells (total fatty acid content of hydrolyzed lipids). Shown are the relative amounts of three replicates (three instances of SCD-1 inhibited LNCaP cells undergoing cell culture).

3.7.4 Comparison of relative abundance of fatty acid isomers between cancer cell lines and associated P values

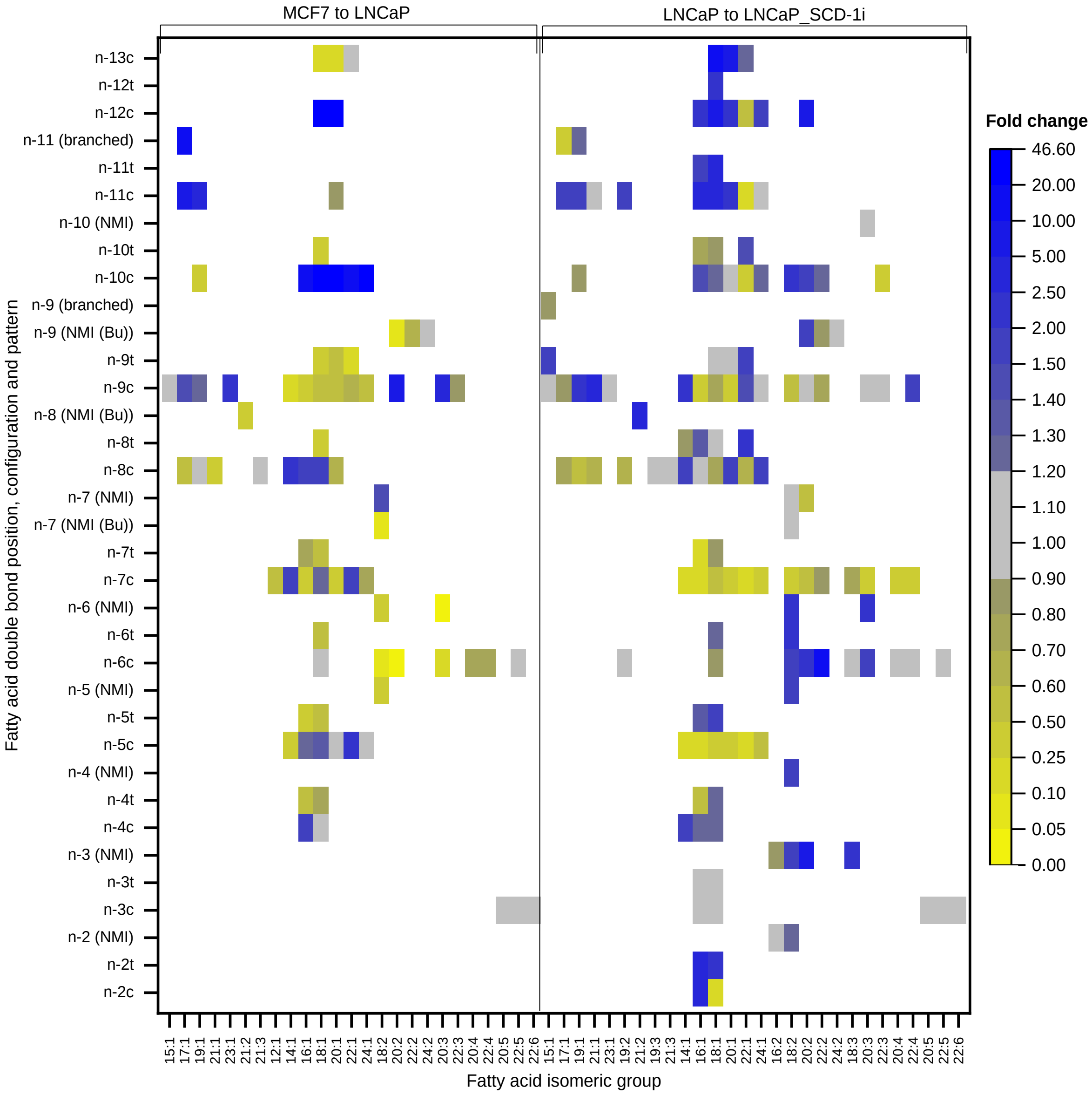

Supplementary Figure 43: Mean fold-change of three biological replicates (n=3) between relative isomer abundances of fatty acid isomers in cancer cell lines MCF7 and LNCaP (left side of the heatmap) as well as between LNCaP and LNCaP_SCD-1i (right side of the heatmap). White colour indicates that the respective fatty acid isomer was not detected in either some or all of the six samples. The colour scale indicates the mean of the fold changes of relative isomer abundance, with a change of less than ± 10% of the value represented by the grey colour and yellow colours indicating a decrease from MCF7 to LNCaP or LNCaP to LNCaP_SCD-1i. Conversely, blue colours indicate an increase in relative abundance.

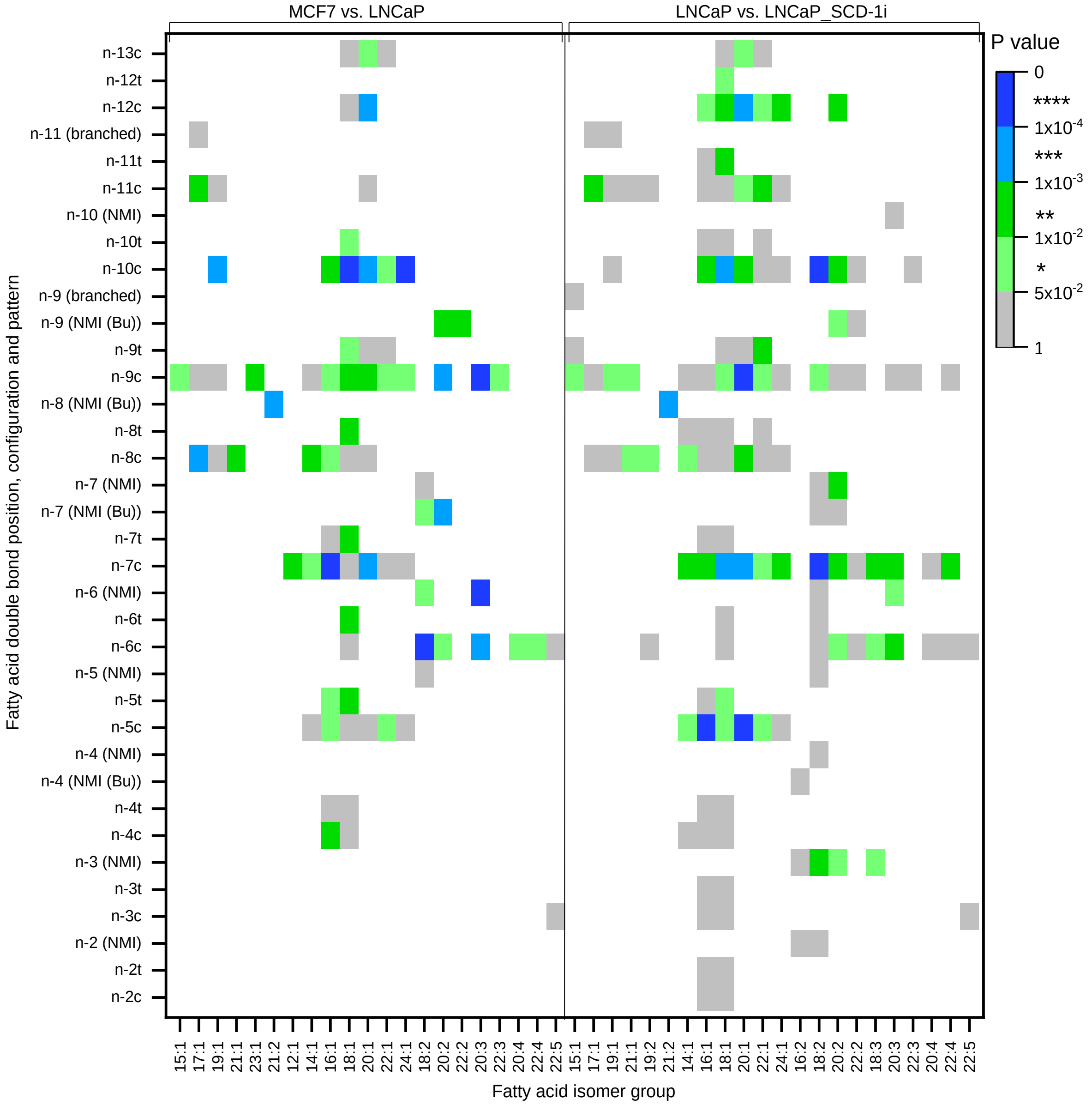

Supplementary Figure 44: P values of fatty acid isomers according to a two-sided Welsh's t-test applied each to the three replicate values (n=3) of the cancer cell lines MCF7 and LNCaP (left side of the heatmap) and LNCaP and LNCaP_SCD-1i (right side of the heatmap). White colour represents either that the respective fatty acid isomer was not detected in any of the six samples or that only one isomer within the fatty acid isomer group was detected, such as FA 22:6*n*-3. The colour scale represents statistical significance of the rejection of the null hypothesis that the respective cell lines exhibit no different relative abundances of fatty acid isomers. P values were calculated with a custom python script employing the python package scipy.

Supplementary Table 8: Fold-changes in relative abundance of selected fatty acid isomers between cell lines MCF7 and LNCaP as well as between LNCaP and LNCaP_SCD-1*i* as well as P values based on two-sided Welsh's t-tests. Statistical details including degrees of freedom, t-test statistic and confidence interval are included in Supplementary Data 8 and 9.

Correlated desaturase (Proposed)	FA isomer desaturation product	MCF7 to LNCaP		LNCaP to LNCaP_SCD-1*i*	
		Fold change	P value	Fold change	P value
SCD-1	FA 14:1*n*-5 *cis*	0.35	0.061	0.21	0.018
SCD-1	FA 16:1*n*-7 *cis*	0.34	0.00004	0.13	0.0043
SCD-1	FA 18:1*n*-9 *cis*	0.56	0.0018	0.77	0.014
SCD-1	FA 17:1*n*-8 *cis*	0.56	0.00019	0.79	0.11
SCD-1	FA 19:1*n*-10 *cis*	0.35	0.0006	0.85	0.41
FADS2	FA 14:1*n*-8 *cis*	2.1	0.0088	1.6	0.01
FADS2	FA 16:1*n*-10 *cis*	19	0.0022	1.5	0.008
FADS2	FA 17:1*n*-11 *cis*	6.1	0.0065	1.8	0.0087
FADS2	FA 18:1*n*-12 *cis*	27	0.051	6.8	0.0035
FADS1	FA 20:3*n*-6,9,15	0.023	0.0000008	2.2	0.048
FADS1	FA 20:2*n*-9,15	0.078	0.0061	1.7	0.041
FADS1	FA 18:2*n*-7,13	0.074	0.014	1.2	0.51
FADS1	FA 18:2*n*-10,13	n.a.	n.a.	2.4	0.0000008
FADS1	FA 18:1*n*-13 *cis*	0.24	0.14	11.2	0.064

3.7.5 Comparison of relative abundance of eicosa- docosa- and tetracosadienoic acids

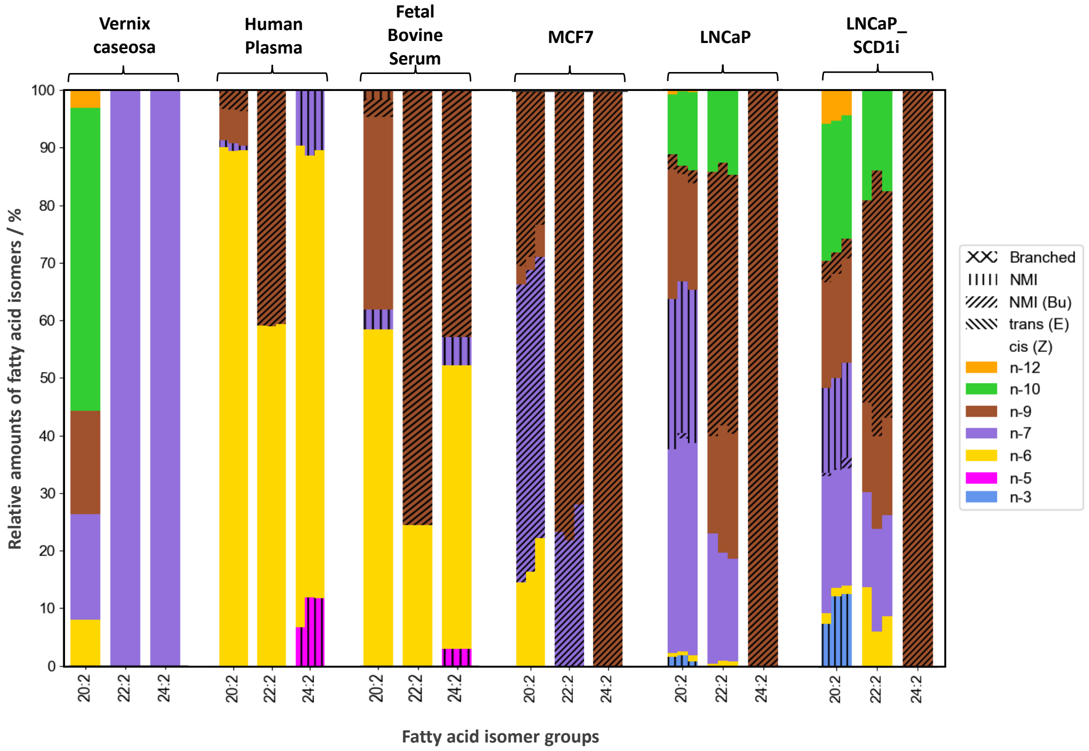

Supplementary Figure 45: Relative abundance of selected fatty acid isomers as observed in the biological contexts studied herein. Not only changes in relative abundance of fatty acid isomers between the samples but importantly the presence of distinct desaturation patterns characterize each aspect of the human or bovine lipidome. This comparative visualization of data that is already shown above in the respective sections supports the claim that analysis of full fatty acid profiles with the OzFAD workflow has the potential to reveal previously unknown molecular markers for unique metabolism including disease specific aberrations.

4 Supplementary references

1 Young, R. S. E. *et al.* Identification of Carbon-Carbon Double Bond Stereochemistry in Unsaturated Fatty Acids by Charge-Remote Fragmentation of Fixed-Charge Derivatives. *Anal. Chem.* **94**, 16180-16188, (2022).

2 Narreddula, V. R. *et al.* Introduction of a Fixed-Charge, Photolabile Derivative for Enhanced Structural Elucidation of Fatty Acids. *Anal. Chem.* **91**, 9901-9909, (2019).

3 Narayana, Vinod K., Tomatis, V. M., Wang, T., Kvaskoff, D. & Meunier, Frederic A. Profiling of Free Fatty Acids Using Stable Isotope Tagging Uncovers a Role for Saturated Fatty Acids in Neuroexocytosis. *Chemistry & Biology* **22**, 1552-1561, (2015).

4 Bollinger, J. G. *et al.* Improved Sensitivity Mass Spectrometric Detection of Eicosanoids by Charge Reversal Derivatization. *Anal. Chem.* **82**, 6790-6796, (2010).

5 Cebo, M. *et al.* Untargeted UHPLC-ESI-QTOF-MS/MS analysis with targeted feature extraction at precursor and fragment level for profiling of the platelet lipidome with ex vivo thrombin-activation. *J. Pharm. Biomed. Anal.* **205**, 114301, (2021).

6 Vickery, J. R. The fatty acid composition of the seed oils of proteaceae: A chemotaxonomic study. *Phytochemistry* **10**, 123-130, (1971).

7 Zhao, J., Fang, M. & Xia, Y. A liquid chromatography-mass spectrometry workflow for in-depth quantitation of fatty acid double bond location isomers. *J. Lipid Res.* **62**, 100110, (2021).

8 Wang, P., Xiong, X., Zhang, X., Wu, G. & Liu, F. A Review of Erucic Acid Production in Brassicaceae Oilseeds: Progress and Prospects for the Genetic Engineering of High and Low-Erucic Acid Rapeseeds (Brassica napus). *Front. Plant Sci.* **13**, (2022).

9 Carballeira, N. M. *et al.* Two Novel Iso-Branched Octadecenoic Acids from a Micrococcus Species. *J. Nat. Prod.* **63**, 1573-1575, (2000).

10 Schantz, M. M., Powers, C. D. & Schleicher, R. L. *Interlaboratory analytical comparison study of total fatty acid concentrations in human serum: results for Exercise 01: QA12FASER01*. (US Department of Commerce, National Institute of Standards and Technology, 2013).

11 Quehenberger, O. & Dennis, E. A. The Human Plasma Lipidome. *N. Engl. J. Med.* **365**, 1812-1823, (2011).

12 Christinat, N., Morin-Rivron, D. & Masoodi, M. High-Throughput Quantitative Lipidomics Analysis of Nonesterified Fatty Acids in Human Plasma. *J. Proteome Res.* **15**, 2228-2235, (2016).

13 Phillips, G. B. & Dodge, J. T. Composition of phospholipids and of phospholipid fatty acids of human plasma. *J. Lipid Res.* **8**, 676-681, (1967).

14 Legrand, P., Catheline, D., Rioux, V. & Durand, G. Lauric acid is desaturated to 12:1n−3 by hepatocytes and rat liver homogenates. *Lipids* **37**, 569-572, (2002).

15 Komoda, M., Inomata, S.-i., Ono, A., Watanabe, H. & Ando, T. Regulation of Sex Pheromone Biosynthesis in Three Plusiinae Moths: Macdunnoughia confusa, Anadevidia peponis, and Chrysodeixis eriosoma. *Biosci. Biotechnol. Biochem.* **64**, 2145-2151, (2000).

16 Onkenhout, W., Venizelos, V., van der Poel, P. F., van den Heuvel, M. P. & Poorthuis, B. J. Identification and quantification of intermediates of unsaturated fatty acid metabolism in plasma of patients with fatty acid oxidation disorders. *Clin. Chem.* **41**, 1467-1474, (1995).

17 Wang, D. H., Wang, Z., Chen, R. & Brenna, J. T. Characterization and Semiquantitative Analysis of Novel Ultratrace C10–24 Monounsaturated Fatty Acid in Bovine Milkfat by Solvent-Mediated Covalent Adduct Chemical Ionization (CACI) MS/MS. *J. Agric. Food. Chem.* **68**, 7482-7489, (2020).

18 Hopkins, C. Y., Chisholm, M. J. & Prince, L. Fatty acids ofLindera umbellata and other Lauraceae seed oils. *Lipids* **1**, 118-122, (1966).

19 Psychogios, N. *et al.* The Human Serum Metabolome. *PLoS One* **6**, e16957, (2011).

20 Navarro, I., Fabriàs, G. & Camps, F. Possible fatty acyl pheromone precursors in Spodoptera littoralis. Search for 11- and 12-hydroxytetradecanoic acids in the pheromone gland. *Lipids* **32**, 407-412, (1997).

21 Saito, H. Identification of novel n-4 series polyunsaturated fatty acids in a deep-sea clam, Calyptogena phaseoliformis. *J. Chromatogr. A* **1163**, 247-259, (2007).

22 Sansone, A., Melchiorre, M., Chatgilialoglu, C. & Ferreri, C. Hexadecenoic Fatty Acid Isomers: A Chemical Biology Approach for Human Plasma Biomarker Development. *Chem. Res. Toxicol.* **26**, 1703-1709, (2013).

23 Wang, D. H. *et al.* Unusual polymethylene-interrupted, Δ5 monounsaturated and omega-3 fatty acids in sea urchin (Arbacia punctulata) from the Gulf of Mexico identified by solvent mediated covalent adduct chemical ionization mass spectrometry. *Food Chem.* **371**, 131131, (2022).

24 Lemaitre, R. N. *et al.* Assessment of frans-Fatty Acid Intake with a Food Frequency Questionnaire and Validation with Adipose Tissue Levels of frans-Fatty Acids. *Am. J. Epidemiol.* **148**, 1085-1093, (1998).
25 Cahoon, E. B., Shanklin, J. & Ohlrogge, J. B. Expression of a coriander desaturase results in petroselinic acid production in transgenic tobacco. *Proc. Natl. Acad. Sci.* **89**, 11184-11188, (1992).
26 Carballeira, N. M., Cruz, H., Hill, C. A., De Voss, J. J. & Garson, M. Identification and Total Synthesis of Novel Fatty Acids from the Siphonarid Limpet Siphonaria denticulata. *J. Nat. Prod.* **64**, 1426-1429, (2001).
27 Feng, G. *et al.* Dual-resolving of positional and geometric isomers of C=C bonds via bifunctional photocycloaddition-photoisomerization reaction system. *Nat. Commun.* **13**, 2652, (2022).
28 Abdelmagid, S. A. *et al.* Comprehensive Profiling of Plasma Fatty Acid Concentrations in Young Healthy Canadian Adults. *PLoS One* **10**, e0116195, (2015).
29 Firl, N., Kienberger, H., Hauser, T. & Rychlik, M. Determination of the fatty acid profile of neutral lipids, free fatty acids and phospholipids in human plasma. *Clin. Chem. Lab. Med.* **51**, 799-810, (2013).
30 Fritsche, J. & Steinhart, H. Contents of trans fatty acids (TFA) in German foods and estimation of daily intake. *Lipid / Fett* **99**, 314-318, (1997).
31 Sun, J.-Y., Wang, X.-K. & Smith, M. A. Identification of n-6 Monounsaturated Fatty Acids in Acer Seed Oils. *J. Am. Oil Chem.' Soc.* **95**, 21-27, (2018).
32 Zheng, W. *et al.* Modular Characteristics and Mechanism of Action of Herbs for Endometriosis Treatment in Chinese Medicine: A Data Mining and Network Pharmacology–Based Identification. *Front. Pharmacol.* **11**, (2020).
33 Wang, M., Han, R. H. & Han, X. Fatty Acidomics: Global Analysis of Lipid Species Containing a Carboxyl Group with a Charge-Remote Fragmentation-Assisted Approach. *Anal. Chem.* **85**, 9312-9320, (2013).
34 Aitzetmüller, K. & Vosmann, K. Cyclopropenoic fatty acids in gymnosperms: The seed oil of Welwitschia. *J. Am. Oil Chem.' Soc.* **75**, 1761-1765, (1998).
35 Candela, M., Astiasarán, I. & Bello, J. Effects of frying and warmholding on fatty acids and cholesterol of sole (Solea solea), codfish (Gadus morrhua) and hake (Merluccius merluccius). *Food Chem.* **58**, 227-231, (1997).
36 Biedermann, W. *et al.* Structural characterisation of some fatty acids from the brain as biomarkers of BSE risk material. *Anal. Bioanal. Chem.* **379**, 1031-1038, (2004).
37 Johns, R. B., Nichols, P. D. & Perry, G. J. Fatty acid composition of ten marine algae from australian waters. *Phytochemistry* **18**, 799-802, (1979).
38 Schlenk, H. & Sand, D. M. A new group of essential fatty acids and their comparison with other polyenoic fatty acids. *Biochim. Biophys. Acta Lipids Lipid Metab.* **144**, 305-320, (1967).
39 Liu, D. *et al.* Fatty acids as natural specific inhibitors of the proto-oncogenic protein Shp2. *Bioorg. Med. Chem. Lett.* **21**, 6833-6837, (2011).
40 Khanh Tran, H. N. *et al.* Investigation of chemical compounds from Chlamydomonas sp. KSF108 (Chlamydomonadaceae). *Biochem. Syst. Ecol.* **83**, 4-6, (2019).
41 Destaillats, F., Wolff, R. L. & Angers, P. A new Δ7-polyunsaturated fatty acid in taxus spp. Seed lipids, dihomotaxoleic (7,11–20:2) acid. *Lipids* **36**, 319-321, (2001).
42 Kendel, M., Barnathan, G., Fleurence, J., Rabesaotra, V. & Wielgosz-Collin, G. Non-methylene Interrupted and Hydroxy Fatty Acids in Polar Lipids of the Alga Grateloupia turuturu Over the Four Seasons. *Lipids* **48**, 535-545, (2013).
43 P. B. Jeppesen, J. S. C. E. H. P. B. M. Effect of High-Dose Growth Hormone and Glutamine on Body Composition, Urine Creatinine Excretion, Fatty Acid Absorption, and Essential Fatty Acids Status in Short Bowel Patients. A Randomized, Double-blind, Crossover, Placebo-controlled Study. *Scand. J. Gastroenterol.* **36**, 48-54, (2001).
44 Hornstra, G., van Houwelingen, A. C., Simonis, M. & Gerrard, J. M. Fatty acid composition of umbilical arteries and veins: Possible implications for the fetal EFA-status. *Lipids* **24**, 511-517, (1989).
45 Montañés, F., Tallon, S. & Catchpole, O. Isolation of Non-methylene Interrupted or Acetylenic Fatty Acids from Seed Oils Using Semi-preparative Supercritical Chromatography. *J. Am. Oil Chem.' Soc.* **94**, 981-991, (2017).
46 Ferreri, C. *et al.* The n-10 Fatty Acids Family in the Lipidome of Human Prostatic Adenocarcinoma Cell Membranes and Extracellular Vesicles. *Cancers* **12**, (2020).
47 Young, R. S. E. *et al.* Apocryphal FADS2 activity promotes fatty acid diversification in cancer. *Cell Rep.* **34**, 108738, (2021).
