# Supplementary material for: Ozone-enabled fatty acid discovery reveals unexpected diversity in the human lipidome
Source: Nat Commun. 2023 Jul 4;14:3940. doi: 10.1038/s41467-023-39617-9 (PMC10319862; doi:10.1038/s41467-023-39617-9)
Supplement: Supplementary file 3 — Description of Additional Supplementary Files [file 41467_2023_39617_MOESM3_ESM.docx]

**Supplementary Data 1:**

Overview of fatty acids that are identified here in NIST1950 Standard Reference Material “Frozen Metabolites in Pooled Human Blood Plasma”. Shown are fatty acids detected in the total fatty acid fraction of the human plasma after lipid extraction, hydrolysis, fixed-charge derivatization and UPLC-OzID-MS (& MS/MS) analysis. Each fatty acid isomer is described according to the n nomenclature (column 1), where the double bond position is indicated as counted from the methyl end, according to the fatty acid shorthand and the systematic name, where the double bond position is indicated as counted from the carboxy end of the fatty acid. For the purpose of the automated database search, systematic names of polyunsaturated fatty acids are generated with *cis* double bonds only. If available at time of analysis (February 2022), the LIPID MAPS ID with a link to the LIPID MAPS webpage is included in column 4 as well as a common name (column 5). Retention times are rounded to three significant digits, and the relative isomer abundance is shown as a percentage of all fatty acid isomers with the same precursor mass. Signal-to-noise ratios are calculated from multiple selected and combined MS/MS spectra of each fatty acid isomer. The confidence of identification is high (H), when the double bond position can be unequivocally determined and low (L), when it can’t be determined, which exact isomer is present. For fatty acid isomers with low confidence of identification, only the presence of certain double bond positions can be ascertained, but not the identity (association of two double bond positions to one specific isomer) of the many coeluting bisunsaturated fatty acid isomers. The absolute quantification is shown in μmol L^-1^ of pooled human plasma. If the fatty acid isomer was identified in the literature (to the best of our knowledge), one reference is given in column 11. If the fatty acid was not identified in human plasma previously, but elsewhere, then a reference is given in column 12. The references are not exhaustive indications as to where the fatty acids have been reported, nor do the references necessarily show the first mention in the literature of the respective fatty acid isomer. If a fatty acid was not found in the literature at all, no reference is shown here, and the column is highlighted with a yellow background. For a few of these fatty acids, we propose common names, indicated in column 5 (italic font). Fatty acids shorter than 12 carbon atoms were not included in the analysis, as these may not be accurately observed due limitations of the extraction and derivatization protocol, see also Supplementary Fig. 11.

**Supplementary Data 2:**

Identification and relative quantification of non-esterified fatty acids (NEFA) in NIST 1950 human plasma standard reference material. The same lipid extract as for the analysis of the total fatty acid content was used here but hydrolysis was omitted for this analysis. Found species and their relative abundance are summarized in Supplementary Figure 26.

**Supplementary Data 3:**

Identified fatty acid species in a vernix caseosa lipid extract (after hydrolysis and fixed-charge derivatization with 4-I-AMPP). Fatty acids that, to the best of our knowledge, have not been reported before, are highlighted with a yellow background, while those that we also discovered in pooled human plasma are highlighted with a grey background.

**Supplementary Data 4:**

Overview of fatty acids that are identified here in fetal bovine serum. Fatty acid isomers that, to the best of our knowledge, have not been reported before, are highlighted with a yellow background, while those that we also discovered in pooled human plasma or vernix caseosa are highlighted with a grey background.

**Supplementary Data 5:**

Overview of fatty acids that are identified here in lipid extracts of MCF7 cells. Column seven shows mean values of relative isomer abundances and their associated standard deviations of three replicates of cell cultures of MCF7 cells. Fatty acids that, to the best of our knowledge, have not been reported before, are highlighted with a yellow background, while those that we also discovered in pooled human plasma or vernix caseosa are highlighted with a grey background. Fatty acid abundance is reported here in nmol per million cells (nmol M-Cells-1) as an estimate of the fatty acid content of each isomer in the cell culture.

**Supplementary Data 6:**

Overview of fatty acids that are identified here in lipid extracts of LNCaP cells. Column seven shows mean values of relative isomer abundances and their associated standard deviations of three replicates of cell cultures of LNCaP cells. Fatty acid abundance is reported here in nmol per million cells (nmol M-Cells-1) as an estimate of the fatty acid content of each isomer in the cell culture.

**Supplementary Data 7:**

Overview of fatty acids that are identified here in lipid extracts of LNCaP_SCD-1i cells. Column seven shows mean values of relative isomer abundances and their associated standard deviations of three replicates of cell cultures of LNCaP_SCD-1i cells. Fatty acid abundance is reported here in nmol per million cells (nmol M-Cells-1) as an estimate of the fatty acid content of each isomer in the cell culture.

**Supplementary Data 8:**

Data for heatmaps showing P values and fold changes regarding the comparison of relative fatty acid quantities in MCF7 cells and LNCaP cells (sheet 1). Sheet 2 shows relevant mean values and standard deviations of the respective relative quantities as well as P values and associated t-test statistics, including degrees of freedom and confidence intervals. The file further contains fold change values and P values that can be readily plotted as volcano plots (these data are source data for Figure 5d).

**Supplementary Data 9:**

Data for heatmaps showing P values and fold changes regarding the comparison of relative fatty acid quantities in LNCaP cells and LNCaP_SCD1i cells (sheet 1). Sheet 2 shows relevant mean values and standard deviations of the respective relative quantities as well as P values and associated t-test statistics, including degrees of freedom and confidence intervals. The file further contains fold change values and P values that can be readily plotted as volcano plots (these data are source data for Figure 5d).
