# Supplementary material for: Ozone-enabled fatty acid discovery reveals unexpected diversity in the human lipidome
Source: Nat Commun. 2023 Jul 4;14:3940. doi: 10.1038/s41467-023-39617-9 (PMC10319862; doi:10.1038/s41467-023-39617-9)
Supplement: Supplementary file 2 — Peer Review File [file 41467_2023_39617_MOESM2_ESM.pdf]

nature portfolio

Peer Review File

Ozone-enabled fatty acid discovery reveals unexpected diversity in the human lipidome

Draft Only

Open Access This file is licensed under a Creative Commons Attribution 4.0 International License, which permits use, sharing, adaptation, distribution and reproduction in any medium or format, as long as you give appropriate credit to the original author(s) and the source, provide a link to the Creative Commons license, and indicate if changes were made. In the cases where the authors are anonymous, such as is the case for the reports of anonymous peer reviewers, author attribution should be to 'Anonymous Referee' followed by a clear attribution to the source work. The images or other third party material in this file are included in the article's Creative Commons license, unless indicated otherwise in a credit line to the material. If material is not included in the article's Creative Commons license and your intended use is not permitted by statutory regulation or exceeds the permitted use, you will need to obtain permission directly from the copyright holder. To view a copy of this license, visit http://creativecommons.org/licenses/by/4.0/.

Editorial Note: This manuscript has been previously reviewed at another journal that is not operating a transparent peer review scheme. This document only contains reviewer comments and rebuttal letters for versions considered at *Nature Communications.*

REVIEWERS' COMMENTS

Reviewer #2 (Remarks to the Author):

With this resubmission and manuscript transfer to Nature Communication journal, authors answered all the questions i posted in my original review. As mentioned before, i think this manuscript is an important step forward in exploration of natural lipidomes and would of interest to many researchers in the field. I would like to acknowledge specifically the efforts authors made to provide GUI version of OzFAD tool.

Draft Only

Reviewer #4 (Remarks to the Author):

Menzel et al. describe a workflow to analyze double-bond (DB) position in fatty acyls (FA) and a semiautomated workflow to deal with information in complex samples. They are identifying an increased number of fatty acyls and determining their double bond position, of which some were not identified so far in human plasma. This is now a very nice experimental line-up and the story reveals new insides into fatty acid diversity. The authors addressed all my comments and concerns. I have no further requests
